# Decrypting Molecular Mechanisms Involved in Counteracting Copper and Nickel Toxicity in Jack Pine (*Pinus banksiana*) Based on Transcriptomic Analysis

**DOI:** 10.3390/plants13071042

**Published:** 2024-04-08

**Authors:** Alistar Moy, Kabwe Nkongolo

**Affiliations:** Biomolecular Sciences Program, Department of Biology, School of Natural Sciences, Laurentian University, Sudbury, ON P3E 2C6, Canada; amoy@laurentian.ca

**Keywords:** biological process, cellular compartment, functional genomic, molecular function, nickel and copper toxicity, *Pinus banksiana*, resistance mechanisms

## Abstract

The remediation of copper and nickel-afflicted sites is challenged by the different physiological effects imposed by each metal on a given plant system. *Pinus banksiana* is resilient against copper and nickel, providing an opportunity to build a valuable resource to investigate the responding gene expression toward each metal. The objectives of this study were to (1) extend the analysis of the *Pinus banksiana* transcriptome exposed to nickel and copper, (2) assess the differential gene expression in nickel-resistant compared to copper-resistant genotypes, and (3) identify mechanisms specific to each metal. The Illumina platform was used to sequence RNA that was extracted from seedlings treated with each of the metals. There were 449 differentially expressed genes (DEGs) between copper-resistant genotypes (RGs) and nickel-resistant genotypes (RGs) at a high stringency cut-off, indicating a distinct pattern of gene expression toward each metal. For biological processes, 19.8% of DEGs were associated with the DNA metabolic process, followed by the response to stress (13.15%) and the response to chemicals (8.59%). For metabolic function, 27.9% of DEGs were associated with nuclease activity, followed by nucleotide binding (27.64%) and kinase activity (10.16%). Overall, 21.49% of DEGs were localized to the plasma membrane, followed by the cytosol (16.26%) and chloroplast (12.43%). Annotation of the top upregulated genes in copper RG compared to nickel RG identified genes and mechanisms that were specific to copper and not to nickel. NtPDR, AtHIPP10, and YSL1 were identified as genes associated with copper resistance. Various genes related to cell wall metabolism were identified, and they included genes encoding for HCT, CslE6, MPG, and polygalacturonase. Annotation of the top downregulated genes in copper RG compared to nickel RG revealed genes and mechanisms that were specific to nickel and not copper. Various regulatory and signaling-related genes associated with the stress response were identified. They included UGT, TIFY, ACC, dirigent protein, peroxidase, and glyoxyalase I. Additional research is needed to determine the specific functions of signaling and stress response mechanisms in nickel-resistant plants.

## 1. Introduction

Nickel and copper are micronutrients that become toxic to plants at high concentrations. The mining and processing of these heavy metals can contaminate soils and the atmosphere, which negatively impacts human health and the environment [1,2,3]. The Greater Sudbury region has an extensive history of copper and nickel pollution and will continue to export these metals [4,5,6,7]. Comparing the responses of plants to copper and nickel is a crucial step in streamlining the remediation strategy and ensuring efficiency and long-term sustainability. As redox heavy metals, copper and nickel both play important roles in plant physiology [8,9]. Copper is a cofactor for electron carriers such as the cytochrome B_6_F complex, plastocyanin, and cytochrome oxidase [10,11,12]. It is, therefore, integral to photosynthesis and cellular respiration. Nickel plays an important role in nitrogen cycling as a cofactor for urease, which is essential for the proper functioning of the ornithine–urea cycle [13,14].

Copper toxicity has many downstream effects and shares symptoms with nickel toxicity. An overabundance of either metal can decrease the concentration of iron, manganese, and zinc, which poses risks to processes that correspond to these metals [15,16,17,18,19,20]. Copper or nickel toxicity can inhibit photosynthesis and cause chlorosis by different means. Excess copper reduces electron transfer in photosystem II by displacing the iron cofactor in plastoquinone QA, inhibiting plastoquinone QB, and preventing the transfer of electrons from tyrosine to P680+ [21,22]. Excess nickel hinders electron transfer by altering the protein conformation of plastoquinone QB and replacing calcium in the oxygen-evolving complex [23,24]. Both excess copper and nickel have been shown to displace magnesium in chlorophyll, leading to decreased chlorophyll content, reduced function, and a decreased capacity to transfer energy to the reaction center [25,26,27,28]. Excess copper also facilitates the Haber–Weiss reaction and Fenton-like reactions, generating superoxide radicals, hydroxyl radicals, and hydrogen peroxide [29,30,31]. Comparatively, excess nickel increases ROS generation by decreasing the activity of antioxidative enzymes such as SOD, CAT, APX, POD, and GSH-Px [32,33]. An abundance of both metals can also cause severe water loss by altering stomata morphology and altering the rate of transpiration on the surface of leaves [34,35,36,37]. Studies on various plants have shown that an overabundance of either metal can lead to necrosis and overall decreased plant growth [38].

Genes associated with copper or nickel resistance have been reported in various species. Membrane-bound transporters such as ZIP and COPT can export copper from the cytosol to the outside of the cell, reducing the initial uptake of copper into the root area [39,40,41,42]. Upregulation of the IREG2 transporter can increase the transport of nickel into the vacuoles of root cells, contributing to vacuolar sequestration [43]. NRAMP transporters may be involved in unloading excess copper or nickel into the xylem prior to root-to-shoot translocation [44,45]. Reports of elevated HMA transporter expression were found to enhance the translocation of excess copper to aerial tissue in some plants, increasing copper resistance [41,46,47]. HMA9, in particular, may be involved in the loading of excess copper in both the xylem and phloem [48]. HMA1 and HMA6/PAA1 can transport excess copper to the stroma of the chloroplasts, while HMA8/PAA2 could transport copper to the thylakoid lumen and plastocyanin [49,50,51,52,53,54]. The delivery of copper to components in the chloroplasts suggests an increase in photosynthesis activity to counteract stress and ensure the homeostasis of metabolites [54,55]. HMA7 may also counteract stress by transporting excess copper to ethylene receptors in the ER, which are involved in the modulation of growth and development [41,56]. Excess copper may also elicit increased CCH expression to reduce the transport of copper to younger leaves, thereby safeguarding newly developed tissue [41,46,57]. Chelators such as MT2a, MT2b, nicotianamine, and histidine have been reported to be present in xylem sap and were correlated with the improved translocation of copper and nickel [58,59,60,61]. In particular, nicotianamine-metal complexes are directly translocated to the aerial component of the plant and may coordinate with YSL transporters to facilitate the loading of copper and nickel into the xylem [45,58,62,63,64]. Glutathione-S-transferases play several roles in copper and nickel resistance, which include increasing antioxidative activity, hormone signal transduction, and the production of the chelator glutathione [65,66].

The discovery of genes in metal-treated plants is crucial to understanding the mechanisms of metal resistance and planning an effective remediation strategy. Many pine species have been reported as having different responses and strategies toward various heavy metals [67,68,69]. The successful utilization of *Pinus banksiana* in the regreening program in the Greater Sudbury Region prompts the investigation of the genetic response to copper toxicity compared to nickel toxicity [70]. Transcriptome analysis will evaluate the genetic similarities or differences in gene expression between nickel-resistant and copper-resistant plants. The survival of this species in metal-contaminated soil also provides a unique opportunity to discover genes that are exclusive to each metal, which is especially important when considering the limitations of the candidate gene identification process. The objectives of this study were to (1) extend the analysis of nickel-resistant and copper-resistant *P. banksiana*, (2) assess the differential gene expression in nickel-resistant genotypes (RGs) compared to copper-resistant genotypes (RGs), and (3) identify mechanisms specific to each metal.

## 2. Results

### 2.1. Nickel and Copper Toxicity

Significant differences were observed in the damage ratings among plants for the groups treated with 1600 mg/kg Ni. In fact, 25% were considered resistant, 40% moderately susceptible, and 35% susceptible. Less damage was observed in the seedlings treated with potassium sulfate. In fact, no significant differences were observed in the seedlings treated with the salt used as a control compared to the water control. For copper, segregation in response to Cu was observed in a group treated with 1300 mg/kg of Cu. Overall, 20% were classified as resistant, 35% as moderately susceptible, and 45% as susceptible. Only resistant and susceptible genotypes, along with samples treated with potassium sulfate and water, were selected in triplicate for the transcriptome analysis.

### 2.2. Transcriptome Analysis

**Figure 1:**
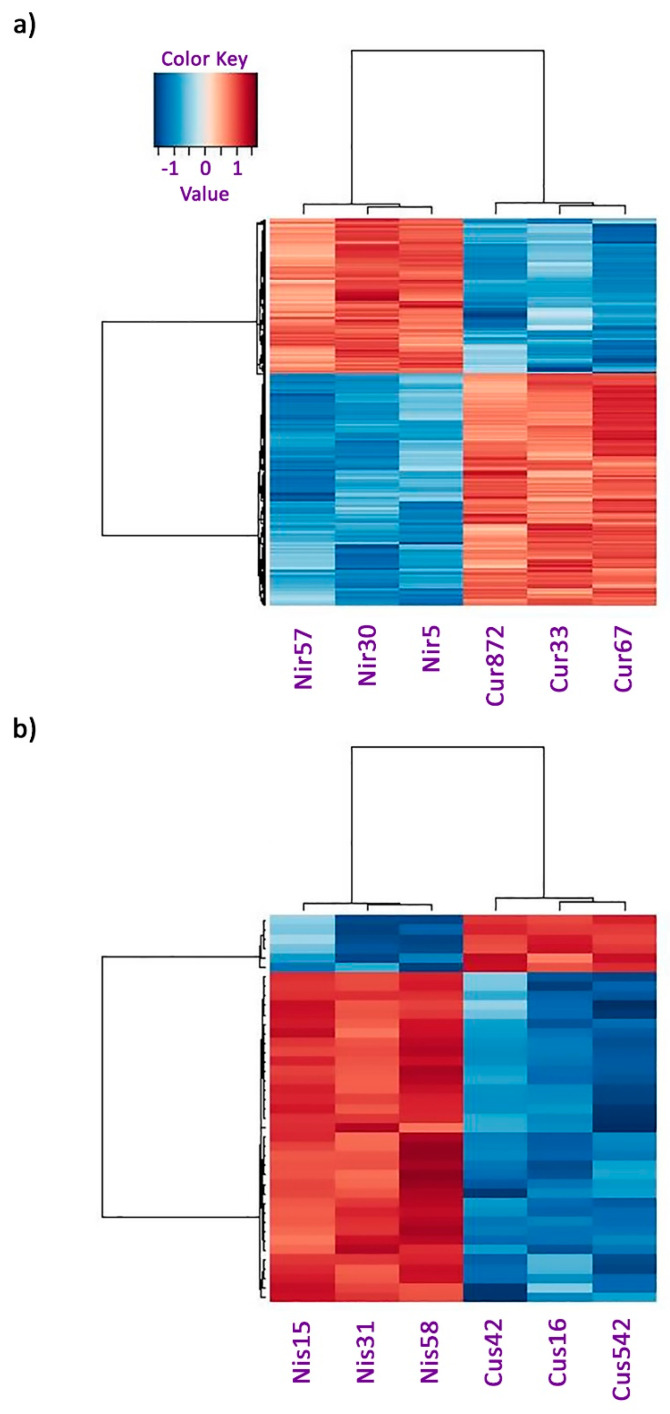
Heatmap of differentially expressed genes in *Pinus banksiana* seedlings for (**a**) the copper resistant compared to nickel resistant genotypes and (**b**) the copper susceptible compared to nickel susceptible genotypes. Differentially expressed gene values for each pairwise comparison were based on the Log2 normalized fold change. Red data represent different levels of upregulation, whereas blue data represent different levels of downregulation. The labels Nir57, Nir30, and Nir5 represent seedlings from the nickel-resistant genotypes. The labels Cur872, Cur33, and Cur67 represent seedlings from the copper-resistant genotypes. The labels Nis15, Nis31, and Nis58 represent seedlings from the nickel-susceptible genotypes. The labels Cus42, Cus16, and Cus542 represent seedlings from the copper-susceptible genotype.

The transcriptome shotgun assembly project has been deposited in the NCBI BioProject database with the accession number PRJNA962116. Differentially expressed genes between copper and nickel-resistant genotypes are described in Table 1. The DEG between copper SG and nickel SG is presented in Table 2. Heatmaps of each pairwise comparison included every DEG between each group, providing an encompassing representation of dynamic gene expression patterns (Figure 1). Both heatmaps showed a high degree of uniformity in gene expression for individuals within a genotype (Figure 1). A heatmap of the DEGs between copper RG and nickel RG revealed a contrast in gene expression for each genotype (Figure 1). Copper RG had a slightly higher number of upregulated genes in comparison to nickel RG, indicating an upregulation of protein production and corresponding processes (Figure 1). A slightly higher number of downregulated genes in nickel RG may suggest the modulation or negative regulation of proteins and associated processes (Figure 1). For copper SG in comparison to nickel SG, the opposite pattern of gene expression was observed, as the majority of genes were upregulated in nickel SG and downregulated in copper SG (Figure 1).

**Figure 2:**
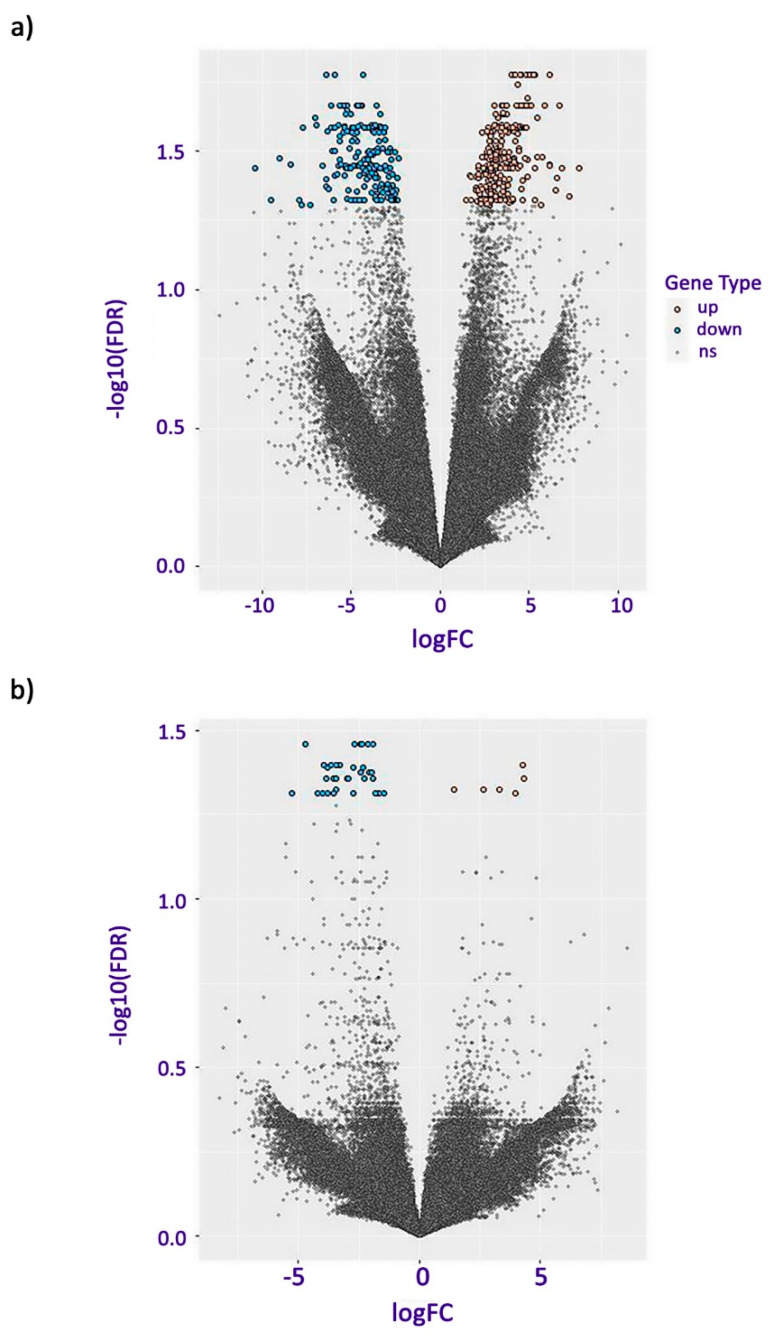
Volcano plot of differentially expressed genes in *Pinus banksiana* seedlings for (**a**) the copper resistance genotype compared to nickel resistance genotypes and (**b**) the copper susceptible genotypes compared to nickel susceptible genotypes. Brown data points represent upregulated gene expression, whereas blue data points represent downregulated gene expression relative to the susceptible genotypes. Grey points indicate no significant difference from the nickel-susceptible genotypes. LogFC is the log-fold change of the copper-susceptible genotypes relative to the nickel-susceptible genotypes. Log10(FDR) is the log10 of the false discovery rate. The barrier between the nonsignificant data points (grey) and the differentially regulated genes (orange or blue) signifies a false discovery rate of 0.05 (two-fold).

The volcano plots showed the degree of spread between the upregulated and downregulated genes in each pairwise comparison (Figure 2). For copper RG in comparison to nickel RG, the majority of DEGs had a fold change of less than 5 and a lower false discovery rate (FDR) in comparison to genes with a higher gene expression fold change or FDR (Figure 2). DEGs with the highest fold change had a lower FDR in comparison to other DEGs (Figure 2). For copper SG, in comparison to nickel SG, there was a relatively small number of DEGs that surpassed the FDR threshold (Figure 2). In addition, there was a larger number of downregulated genes with a relatively even distribution (Figure 2).

**Figure 3:**
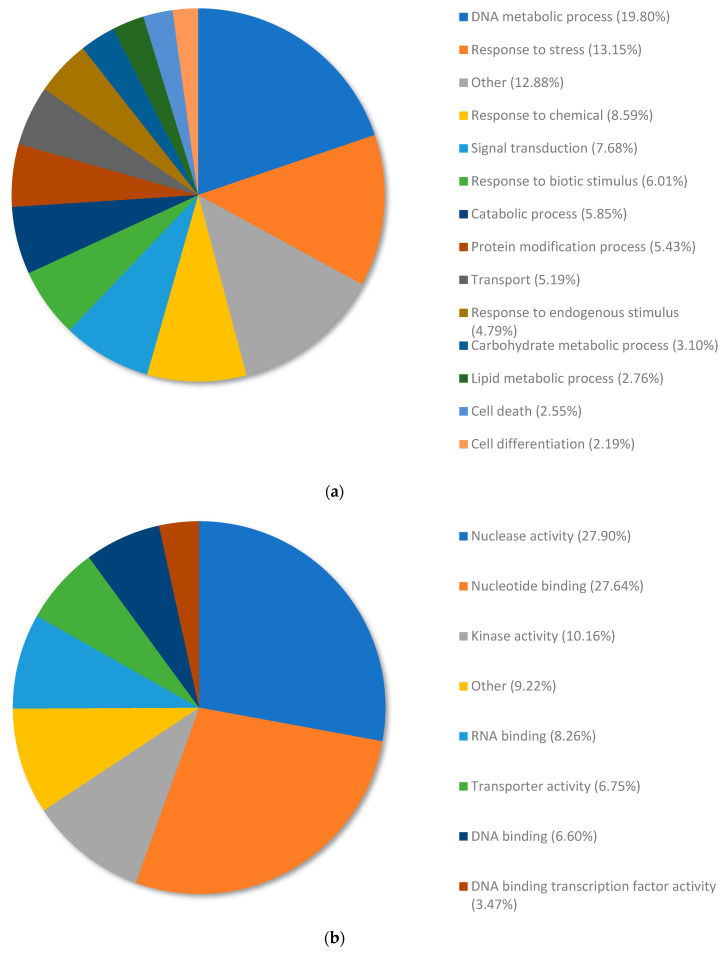
Percent distribution of differentially expressed genes (DEGs) for (**a**) biological processes for copper resistant (RG) compared to nickel resistant (RG) and (**b**) molecular functions for copper RG compared to nickel RG. DEGs from copper RG compared to nickel RG were annotated and allocated to terms within the biological processes category and molecular function category using Omicsbox/Blast2GO. Terms that had a total percentage of expressed genes lower than 2% were collectively categorized under the term “other.”

DEGs from the transcriptomes of copper RG compared to nickel RG were annotated and allocated to terms within the biological processes category using Omicsbox/Blast2GO (Figure 3). Table 3 and Table S2 illustrate top upregulated genes when copper RG was compared to nickel RG. Table 4 and Table S3 illustrate the top downregulated genes for copper RG compared to nickel RG. Table 5 shows the top upregulated genes when copper SG was compared to nickel SG, and Table 6 and Table S4 show the downregulated genes for copper SG vs. nickel SG.

Overall, 449 DEGs were annotated and organized into the following subcategories of biological processes: DNA metabolic process (19.80%), response to stress (13.15%), response to chemicals (8.59%), signal transduction (7.68%), response to biotic stimulus (6.01%), catabolic process (5.85%), protein modification process (5.43%), transport (5.19%), response to endogenous stimulus (4.79%), carbohydrate metabolic process (3.10%), lipid metabolic process (2.76%), cell death (2.55%), and cell differentiation (2.19%) (Figure 3a) Terms that had a total percentage of expressed genes lower than 2% were collectively categorized under the term “other” (12.88%).

DEGs from copper RG compared to nickel RG were annotated and allocated to terms within the metabolic function category using Omicsbox/Blast2GO (Figure 3b). Overall, 449 DEGs were annotated and organized into the following subcategories of metabolic functions: nuclease activity (27.90%), nucleotide binding (27.64%), kinase activity (10.16%), RNA binding (8.26%), transporter activity (6.75%), DNA binding (6.60%), and DNA binding transcription factor (3.47%). Terms that had a total percentage of expressed genes lower than 2% were collectively categorized under the term “other” (9.22%).

DEGs from copper RG compared to nickel RG were annotated and allocated to terms within the cellular compartment category using Omicsbox/Blast2GO (Figure S1). The 449 DEGs were annotated and organized into the following cellular compartment locations: plasma membrane (21.49%), cytosol (16.26%), chloroplast (12.43%), extracellular region (11.31%), mitochondrion (9.48%), Golgi apparatus (5.90%), endoplasmic reticulum (5.47%), and nucleoplasm (3.37%). Terms that had a total percentage of expressed genes lower than 2% were collectively categorized under the term “other” (14.28%).

**Figure 4:**
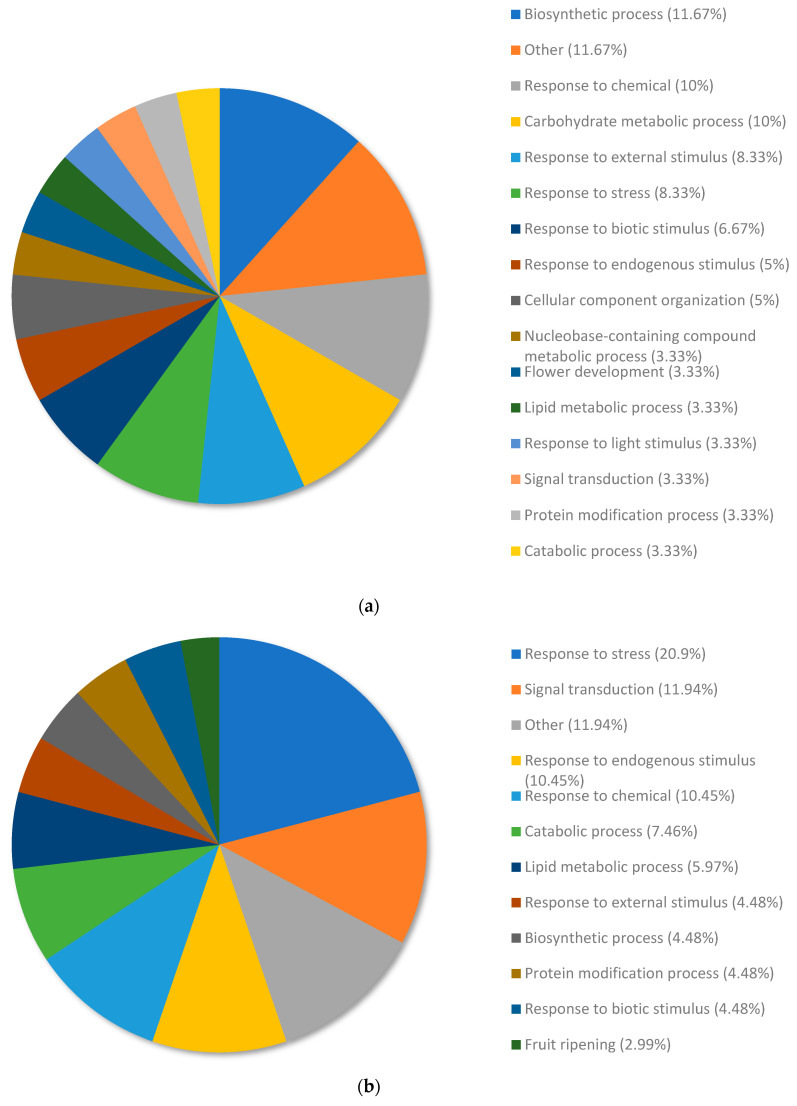
Percent distribution of the (**a**) top 100 upregulated genes in biological processes for copper resistant (RG) compared to nickel resistant (RG) and the (**b**) top 100 downregulated genes in biological processes for copper RG compared to nickel RG. The top upregulated and downregulated genes from copper RG compared to nickel RG were annotated and allocated to terms within the biological process category using Omicsbox/Blast2GO. Terms that had a total percentage of expressed genes lower than 2% were collectively categorized under the term “other.”

The top upregulated genes from copper RG compared to nickel RG were annotated and allocated to terms within the biological process category using Omicsbox/Blast2GO (Figure 4a). The upregulated genes were annotated and organized into the following subcategories of biological processes: biosynthetic process (11.67%) and response to chemicals (10%), carbohydrate metabolic process (10%), response to external stimulus (8.33%), response to stress (8.33%), response to biotic stimulus (6.67%), response to endogenous stimulus (5%), cellular component organization (5%), nucleobase-containing compound metabolic process (3.33%), flower development (3.33%), lipid metabolic process (3.33%), response to light stimulus (3.33%), signal transduction (3.33%), protein modification process (3.33%), and catabolic process (3.33%). Terms that had a total percentage of expressed genes lower than 2% were collectively categorized under the term “other” (11.67%).

The top downregulated genes from copper RG compared to nickel RG were annotated and allocated to terms within the biological process category using Omicsbox/Blast2GO (Figure 4b). The downregulated genes were annotated and organized into the following subcategories of biological processes: response to stress (20.9%), signal transduction (11.94%), response to endogenous stimulus (10.45%), response to chemicals (10.45%), catabolic process (7.46%), lipid metabolic process (5.97%), response to external stimulus (4.48%), biosynthetic process (4.48%), protein modification process (4.48%), response to biotic stimulus (4.48%), and fruit ripening (2.99%). Terms that had a total percentage of expressed genes lower than 2% were collectively categorized under the term “other” (11.94%).

**Figure 5:**
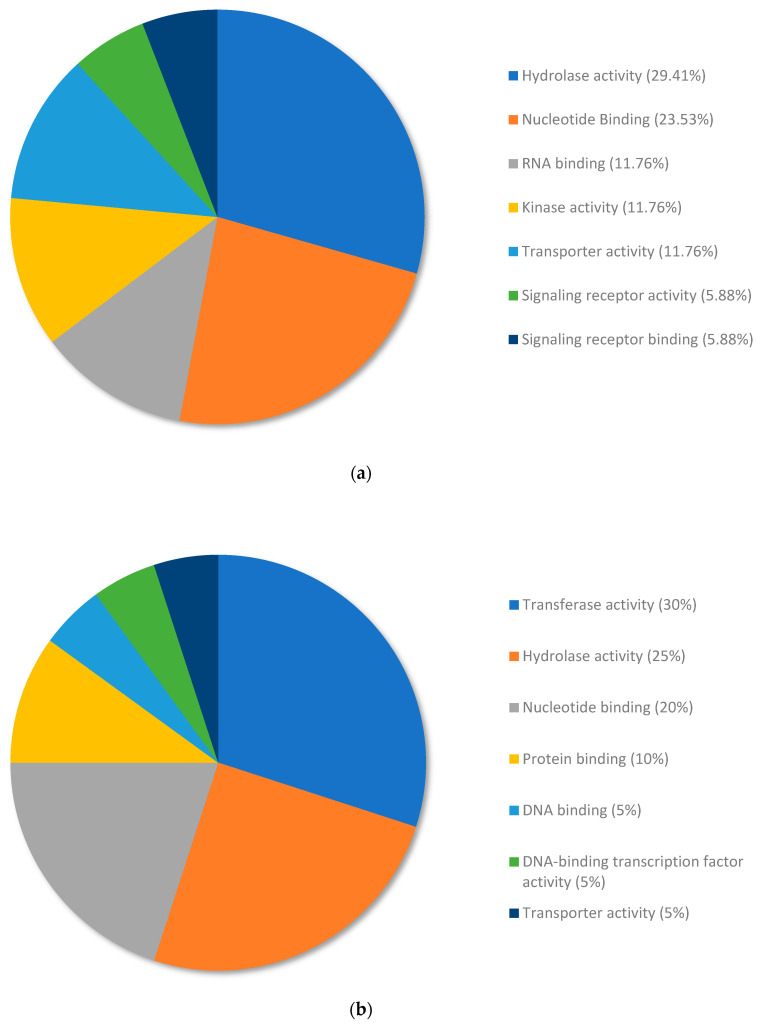
Percent distribution of the (**a**) top 100 upregulated genes in molecular function for copper resistant RG compared to nickel resistant (RG) and the (**b**) top 100 downregulated genes in molecular function for copper RG compared to nickel RG. The top upregulated and downregulated genes from copper RG compared to nickel RG were annotated and allocated to terms within the molecular function category using Omicsbox/Blast2GO. Terms that had a total percentage of expressed genes lower than 2% were collectively categorized under the term “other.”

The top upregulated genes from copper RG compared to nickel RG were annotated and allocated to terms within the molecular function category using Omicsbox/Blast2GO (Figure 5a). The upregulated genes were annotated and organized into the following subcategories of molecular function: hydrolase activity (29.41%), nucleotide binding (23.53%), RNA binding (11.76%), kinase activity (11.76%), transporter activity (11.76%), signal receptor activity (5.88%), and signaling receptor binding (5.88%).

The top downregulated genes from copper RG compared to nickel RG were annotated and allocated to terms within the molecular function category using Omicsbox/Blast2GO (Figure 5b). The downregulated genes were annotated and organized according to the following subcategories of molecular function: transferase activity (30%), hydrolase activity (25%), nucleotide binding (20%), protein binding (10%), DNA binding (5%), DNA-binding transcription factor activity (5%), and transporter activity (5%).

**Figure 6:**
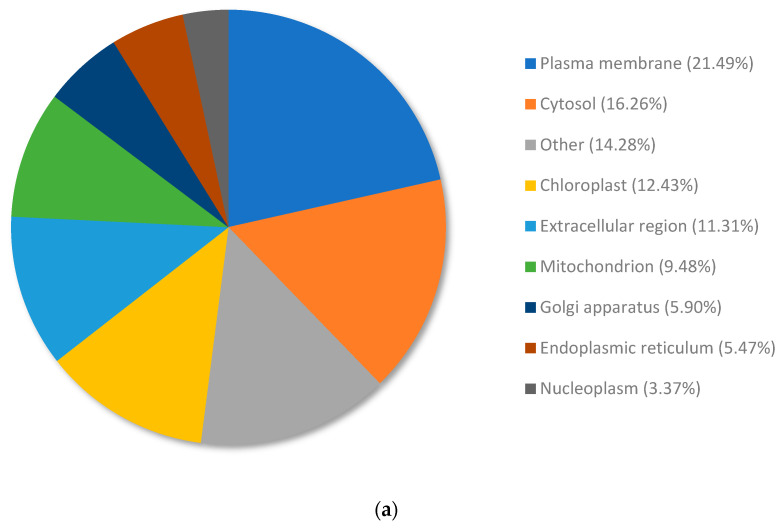

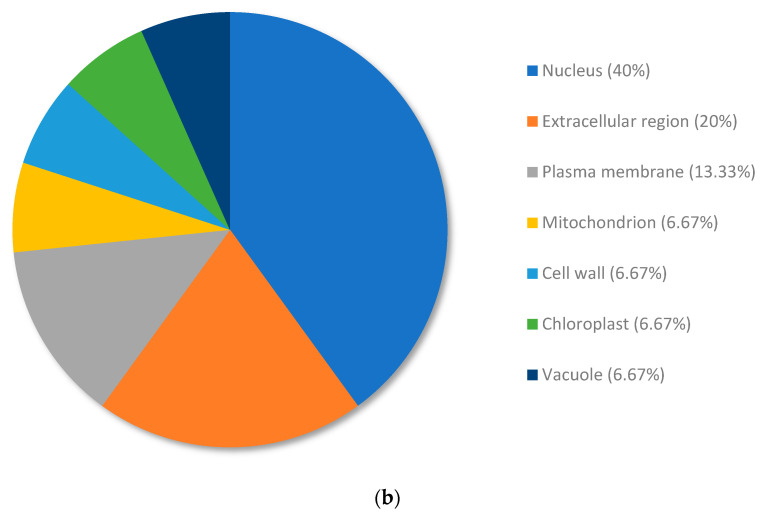
Percent distribution of the (**a**) top 100 upregulated genes in cellular compartment location for copper resistant (RG) in comparison to nickel resistant (RG) and the (**b**) top 100 downregulated genes in cellular compartment location for copper RG in comparison to nickel RG. The top upregulated and downregulated genes from copper RG compared to nickel RG were annotated and allocated to terms within the cellular compartment category using Omicsbox/Blast2GO. Terms that had a total percentage of expressed genes lower than 2% were collectively categorized under the term “other.”

The top upregulated genes from copper RG compared to nickel RG were annotated and allocated to terms within the cellular compartment category using Omicsbox/Blast2GO (Figure 6a). The upregulated genes were annotated and organized into the following cellular compartment locations: plasma membrane (21.49%), cytosol (16.26%), chloroplast (12.43%), extracellular region (11.31%), mitochondrion (9.48%), Golgi apparatus (5.90%), endoplasmic reticulum (5.47%), and nucleoplasm (3.37%). Terms that had a total percentage of expressed genes lower than 2% were collectively categorized under the term “other” (14.28%).

The top downregulated genes from copper RG compared to nickel RG were annotated and allocated to terms within the cellular compartment category using Omicsbox/Blast2GO (Figure 6b). The downregulated genes were annotated and organized into the following cellular compartment locations: nucleus (40%), extracellular region (20%), plasma membrane (13.33%), mitochondrion (6.67%), cell wall (6.67%), chloroplast (6.67%), and vacuole (6.67%).

## 3. Discussion

### 3.1. Physiological Mechanisms in P. banksiana Dealing with Soil Nickel and Copper Contamination

Plants growing on metal-contaminated soils are classified as resistant and have adapted to this stressed environment. The physiological copying mechanism of *P. banksiana* has been described by Moarefi and Nkongolo [71]. In general, plants cope with metal contamination by using either avoidance and/or tolerance strategies. Metal-avoider plants use a strategy that prevents the entry of metal ions into their cells. Tolerant plants detoxify metal ions that have entered their cells by crossing the plasma membrane or the biomembranes of internal organelles. Three categories have been identified among tolerant plants. Excluders maintain a low level of metal in their aerial tissues when growing on metal-contaminated soils. Metals enter root cells, but they prevent their movement from the root to aerial tissues. Indicators accumulate metals in their above-ground tissues, but the levels found in these areal tissues are reflective of the metal concentration in the soil (the metal concentration in their above-ground tissues is similar to the levels found in the soil). In accumulators/hyperaccumulators, metals that have entered the roots are translocated and accumulated in their areal tissues [72,73]. Based on this classification, *P. bnkasiana* was classified as a metal indicator [71].

### 3.2. DEG Analysis Reveals Different Patterns of Gene Expression between Copper RG and Nickel RG

The number of DEGs between genotypes can provide information on the overall response of the copper-resistant genotype in comparison to the nickel-resistant genotype. The hundreds of DEGs between copper RG and nickel RG suggest that some mechanisms are differentially regulated in response to metal stress. Although the majority of the transcriptome was similar, a small number of genes can often orchestrate significant processes and changes pertaining to heavy metal resistance [74,75]. The presence of 449 DEGs at high stringency most likely contributed to differences between the response to copper and the response to nickel. The different number of upregulated and downregulated genes between genotypes infers the use of different physiological mechanisms to achieve a similar endpoint phenotype. The presence of more upregulated genes in copper RG suggests more direct mechanisms that confront copper toxicity with the increased production of proteins and associated processes. In contrast, the presence of more downregulated genes in nickel RG indicates the negative regulation of certain processes or signaling pathways. For example, some phytohormones and pathways may require a controlled response with limitations on protein production as a means to optimize the heavy metal stress response and prevent cellular toxicity [76]. For copper SG compared to nickel SG, there were only 41 DEGs at high stringency, indicating a high similarity in gene expression between susceptible plants. This finding suggests that susceptible plants have less targeted specificity when responding to copper or nickel stress. Susceptible plants often lack the expressed genes needed to ameliorate heavy metal toxicity [76,77,78]. The similarity between the genotypes suggests that gene expression was not directly involved with the detoxification of heavy metals but was instead managing downstream stress mechanisms and tissue damage. After a certain point, the cascading effect of heavy metals can manifest itself as a blanket stress effect, causing nonspecific tissue damage and cytotoxicity [37,79]. The higher number of upregulated genes in nickel SG may indicate attempts to increase processes that mitigate nickel stress, counteract cascading downstream effects, or restrict the accumulation of toxic secondary metabolites induced by excess nickel. In contrast, the higher number of downregulated genes in copper SG may suggest the negative regulation of proteins and metabolites that would be otherwise toxic at higher concentrations [66]. Susceptible plants may utilize shared stress mechanisms associated with reducing tissue damage, managing the turnover of metabolites, and responding to necrosis [80,81,82,83]. No biomarkers specific to copper or nickel susceptibility were identified in susceptible genotypes, although general stress response mechanisms were identified.

The DNA metabolic process had the highest proportion of gene expression, which suggests different levels of DNA regulation between copper and nickel. The DNA metabolic process encompasses processes such as DNA methylation, modification, repair, processing, maintenance of repetitive sequences, and telomere maintenance [84]. Excess copper causes double-stranded breaks, DNA oxidation, and an overall reduction in DNA content [85,86,87]. Excess nickel has also been shown to elicit DNA damage and reduce DNA content, although the specific mechanisms are not well documented in plants [88,89]. The high proportion of genes associated with nuclease activity indicates different levels of DNA repair mechanisms, which may be used to mitigate the oxidative stress on DNA induced by heavy metals [90]. It implies a possible function related to DNA methylation and the epigenetic modulation of gene expression. Multiple plants differ in global methylation patterns elicited by excess copper and nickel [88,91,92,93]. Different methylation patterns may also persist in later developmental stages to facilitate long-term adaptation to metal-contaminated sites [91]. DNA methylation can orchestrate significant changes to plant physiology, especially at earlier stages of plant development [94]. Genes were ranked based on Log2 FC from each pairwise comparison.

Considering that the samples were replicated three times and only the DEG detected at high stringency cut-offs, based on the false discovery rate (FDR) analysis, were selected, it is highly unlikely that the reported gene expression variations were due to chance.

The response to stress and other related terms had a high proportion of gene expression that indicated physiological differences in stress mitigation toward copper and nickel. Both excess copper and nickel can alter the homeostasis of other heavy metal ions, decrease photosystem II functionality, reduce chlorophyll function, and alter phytohormone distribution during early development [15,16,18,21,23,95,96,97,98]. The response to stress term is also highly expressed in the top upregulated and downregulated genes for copper RG and nickel RG, with nickel RG having a comparatively higher allocation of gene expression. Differences in the physiological impact of copper and nickel are likely responsible for this significant difference.

### 3.3. Analysis of the Top Upregulated Genes in Copper-Resistant Plants Compared to Nickel-Resistant Genotypes Reveals Mechanisms That Are Associated with Copper Resistance in Pinus banksiana

Describing the top upregulated genes of copper-resistant plants compared to nickel-resistant plants allowed for the identification of mechanisms associated with the response to copper. Analysis of the top upregulated genes also corroborated previous findings on copper resistance, establishing mechanisms that are specific to copper and are potentially involved in copper detoxification and tolerance [99]. Additionally, another transporter associated with copper resistance was identified, demonstrating the efficacy of transcriptome analyses on multiple pairwise comparisons for describing gene expression.

Two heavy metal transporters were identified as exclusive to copper in comparison to nickel. One of the most expressed genes with several isoforms encodes the pleiotropic drug resistance protein 1 (NtPDR1), which is an ATP-binding cassette (ABC) transporter belonging to the ABC G subfamily [100,101]. ABC transporters are membrane-bound and use ATP hydrolysis to facilitate the active transport of entities [102,103]. The conserved protein regions of ABC transporters include two nucleotide-binding folds associated with ATP hydrolysis and two hydrophobic transmembrane domains involved in the determination of substrate specificity [102,104]. Upregulation of AtPDR family transporters in *Arabidopsis thaliana* increased cadmium resistance and lead resistance by exporting these metals from the cytosol to the apoplast [105,106]. It has been proposed that PDR may serve a similar function in *Pinus banksiana* by exporting excess copper from the cytosol and reducing the overall concentration of copper in the cell. PDR may also be involved in the general stress response, as demonstrated by the upregulation of Ospdr9 in response to hormones such as jasmonic acid and ABA [107,108].

One of the top upregulated genes encoded for the Yellow Stripe 1 (YS1) transporter is a transmembrane transporter that facilitates the movement of heavy metals between intercellular and intracellular compartments (Table 3a) [64,109,110]. YSLs have a conserved oligopeptide transporter domain that binds to metal complexes and oligopeptides [111]. YS1 has been shown to import various heavy metals complexed with nicotianamine (NA) from the rhizosphere into the root cells [109,112]. YSL3 can initiate the loading of metal-NA complexes from the apoplast into the xylem and mediate the root-to-shoot translocation of iron and cadmium [64,113,114]. In *Brachypodium distachyon*, BdYSL3 can load copper-NA into the phloem and redistribute copper-NA complexes to the flowers and reproductive organs [110]. Similarly, OsYSL16 in *Orzya sativa* redistributes copper-NA complexes from the phloem to the seeds and younger leaves [115]. The dynamic response of YSL family proteins to alterations in heavy metal content may differ between species, highlighting the different strategies that may be utilized in response to excess heavy metals. In *Oryza sativa*, YSL6 conferred manganese resistance by increasing the import of manganese into the symplast, which reduced manganese accumulation and subsequent damage in the apoplast [116]. The upregulation of YSL7 in transgenic tobacco conferred resistance to excess iron, cadmium, and nickel by increasing the translocation of these metals to the shoots and seeds, which may have allowed for effective sequestration [117]. The role of YSL in mediating xylem loading and translocation has yet to be described in copper-treated plants. In response to excess copper, AtYSL2 transports copper-NA complexes laterally within the xylem and phloem [118]. Upregulation of YSL3.1 in transgenic tobacco and *Oryza sativa* led to copper accumulation in younger leaves and conferred copper resistance [119]. Nicotianamine is the most commonly reported chelator for copper due to having a higher binding affinity in comparison to other metals, although it is also possible for copper to form a complex with phytosiderophore or be transported as an ion in rare circumstances [109,111]. It is, therefore, possible for YSL upregulation to be associated with lateral transport in the vasculature or the redistribution of copper from the phloem to younger leaves in *Pinus banksiana* [110,118]. Redistribution of copper to the younger needles may serve as a sync for copper sequestration, especially if older needles are weaker or more prone to potential toxicity. It is unlikely for YSL to redistribute excess copper to the seeds, as this area is commonly targeted by heavy metals to hinder development and germination [120,121].

Another highly expressed gene was the heavy metal-associated isoprenylated plant (HIPP), which is a metallochaperone that captures and transports heavy metal ions to proteins located in other cellular compartments (Table 3a) [122]. HIPPs comprise a heavy metal-associated (HMA) domain associated with directly binding to free metal ions and a cysteine-rich isoprenylation site that interacts with other proteins, enzymes, and membranes [122,123]. Variation in the isoprenylation site may modify cellular compartment localization, the site of protein targeting, and overall signal transduction [122,124,125,126]. As a divalent ion, copper can bind to other metallochaperones, such as ATFP3 and ATFP6 in *Arabidopsis thaliana*, supporting the potential role of HIPP in copper chelation and homeostasis [122,126,127]. HIPP may be involved in copper resistance, as it has been shown to confer heavy metal resistance in yeast and *Arabidopsis thaliana* [128,129]. HIPP conferred resistance to excess copper and cadmium in *Camellia sinensis* exposed to excess copper or cadmium [130]. The in vitro binding of HIPP to copper, cadmium, and zinc also corroborates this proposed function [126,127]. Knockout of OsHIPP17 in *Oryza sativa* reduced the expression of copper transporters and cytokinin-related genes, demonstrating crosstalk with other transporters and signaling proteins [131]. In *Pinus banksiana*, HIPP may potentially bind to and trap copper, reducing the effects of copper toxicity on plant physiology [132].

Among the top DEGs were two genes associated with photosystem II, encompassing the GO terms response to light stimulus and response to stress. One of the top upregulated genes encodes the blue copper protein (BCP), which catalyzes the reduction and oxidation of substrates, subsequently facilitating the transport of electrons from an electron donor to an electron acceptor [133,134]. Excess copper can reduce chloroplast function and inhibit photosynthesis by replacing ions in enzymes involved in the electron transport chain [21,23,95]. Increased plastocyanin expression can recover the frequency of electron transfer reactions and improve photosynthesis, thereby counteracting copper-induced photodamage and maintaining a usable pool of carbohydrates for other metabolic needs. In *Glycine max*, excess copper upregulated plastocyanin expression as part of a concerted effort to increase the rate of photosynthesis [135]. Alternatively, the BCP gene may also encode proteins in the stellacyanin subfamily, which could be involved in facilitating redox reactions with substrates in the cell wall [136,137]. In addition to having a low redox potential, stellacyanin has a redox-active copper-binding domain, which can aid in lignin synthesis or mediate the stress response [136,137,138]. The upregulated expression of BCP in some plants resulted in higher lignin production, which could act as a counteractive response to heavy metal stress by strengthening the cell wall and enhancing its defense properties [139,140]. The lignin production function of the BCP gene is supported by the expression of other top regulated genes in *Pinus banksiana,* which are involved in enhancing the structural integrity of the cell wall. Further research is needed to reveal the molecular structure, function, and subfamily classification of the blue-collar protein.

Another top upregulated gene that was associated with photosynthesis function is a gene encoding the 22 kDa Photosystem II protein, which facilitates the non-photochemical quenching of energy released by pigment-mediated fluorescence (Table 3a) [141,142]. Light absorption causes chlorophyll a, chlorophyll b, and xanthophyll to fluoresce, releasing energy that can be utilized in photosynthesis reactions and O_2_ radical production [143,144,145,146]. Non-photochemical quenching captures and dissipates the released energy as heat, preventing the production of O_2_ radicals, which damage components of the photosynthetic process [141,142,143]. Excess copper has been shown to decrease chlorophyll concentration, alter chlorophyll morphology, and reduce enzyme activity associated with photosystem II [95]. The copper-mediated generation of ROS can further exasperate these symptoms by targeting thylakoid membranes and chloroplast membranes [29,30,31]. The upregulation of the 22 kDa photosystem II protein could increase non-photochemical quenching, thereby reducing ROS-mediated damage and subsequent photodamage [146,147]. The 22 kDa Photosystem II protein and the blue copper protein may be upregulated to prevent photodamage caused by copper and protect photosynthesis machinery. Both proteins have a similar function to the light-inducible protein, which was identified in a transcriptome analysis between copper-resistant and susceptible plants [99].

The high proportion of genes associated with the biosynthetic process and the carbohydrate metabolic process play a crucial role in cell wall metabolism (Table 3a). Many of the top upregulated genes have annotated functions that correspond to the protection and reinforcement of the cell wall. One of the top upregulated genes encoded for Shikimate O-hydroxycinnamoyltransferase (HCT), which is involved in lignin synthesis [148,149]. HCT catalyzes the conversion of p-coumaroyl-CoA, shikimate, or quinic acid to 4-coumaroylshikimate [148,149,150]. HCT also catalyzes the conversion of 5-o-caffeoylshikimic to caffeoyl-CoA and the subsequent conversion of caffeoyl-CoA to shikimate [151]. The initial set of reactions in the phenylpropanoid pathway directs substrates and intermediates through a reaction cycle that eventually yields the lignin monomers S-lignin, H-lignin, and G-lignin [152]. HCT can thus act as a crucial control point for lignin synthesis [153,154]. In addition to altering the proportions of monolignin subunits, silencing HCT can lead to an overall reduction in lignin content, an increase in cellulose, a decrease in cell wall thickness, and an increase in lumen diameter [154,155,156,157,158]. In *Zoysia japonica*, upregulation of HCT increased the ratio of the S-lignin monomer and elevated the activity of lignin-associated enzymes via phytohormone signaling [151]. In transgenic tobacco, overexpression of HCT increased lignification and the number of secondary cell layers, which provided increased stem strength and rigidity [159]. Lignin is an important contributor to the formation of secondary cell walls in fibers and vasculature [160]. The hydrophobic domains in lignin provide waterproofing properties that are essential for separating anatomical structures [161]. Lignin also crosslinks with xylan and cellulose, providing additional mechanical strength, rigidity, and thickness to the cell wall [162,163]. Some researchers have proposed that lignin may be able to block and reduce the transport of heavy metals to other areas of the plant [164]. Polysaccharides, or functional groups, in the cell wall can bind to copper and act as a sync for copper sequestration, which may prevent the intracellular transport of copper to other organelles, such as the chloroplast and vacuole [165]. The identification of top upregulated genes involved in protecting the photosynthesis machinery and the chloroplast corroborates this proposed function.

A gene encoding the cellulose synthase-like E6 (CslE6) protein was also among the top upregulated genes related to cell wall metabolism (Table 3a). Csl family proteins catalyze the addition of UDP-glucose to the beta-1,4 glycan polymer within cellulose [166]. Csl family proteins have also been shown to catalyze the synthesis of hemicelluloses such as mannans, glucomannans, xylans, xyloglucans, and homogalacturonan [167,168,169,170,171]. Cellulose comprises the most abundant portion of the primary and secondary cell walls, whereas hemicellulose comprises a considerable portion of the secondary cell wall [172]. Cellulose functions as a load-bearing microfibril that imparts tensile strength and turgor pressure to the cell wall, thereby maintaining the structure and morphology of the cell wall [173,174,175]. Hemicellulose crosslinks with cellulose and lignin, providing structural support and reinforcement for the cell wall [176]. In particular, the presence of hemicellulose lignin matrices provides resilience against shearing forces [177]. In cadmium-tolerant *Arabidopsis thaliana*, cadmium has been found to bind to cellulose and hemicellulose [178]. In some plants, Csl can maintain cell division for normal development and provide thickness and density to the cell wall [171,179]. Overexpression of CslD3 in defective *Arabidopsis* has been shown to increase cell elongation and rescue the integrity of the cell wall [180]. The upregulation of Csl genes can lead to higher cellulose production, mechanical strength, and overall mass in the primary and secondary cell walls [181].

Another top upregulated gene that was associated with cell wall metabolism encoded mannose-1-phosphate guanylyltransferase (MPG). MPG catalyzes the production of GDP-mannose, which is involved in the N-glycosylation of proteins embedded in the cell wall [182]. N-glycosylation is a post-translational modification that attaches a sugar residue to the free amine group of asparagine within the sequence asparagine-X-Serine/Threonine [183]. N-glycosylation can alter various properties such as polarity, solubility, reactivity, and cell compartment localization, which can alter protein conformation and overall function [183,184,185,186]. GDP mannose is also a precursor for GDP-fucose, which is a component of the cell wall that is involved in adhesion, polysaccharide crosslinking, and lignification [187,188,189,190]. Additionally, GDP mannose is a precursor for ascorbic acid, which is a multifunctional compound involved in stress signaling, defense processes, and crosstalk with phytohormones [188,191,192]. In *Arabidopsis thaliana*, knockout of the MPG gene leads to callose deposition in the primary cell wall, incomplete construction of the cell wall, and a considerable decrease in cellulose content [182]. Upregulation of MPG in *Pinus banksiana* could potentially mitigate copper toxicity by producing GDP-mannose, which contributes to the reinforcement of the cell wall.

A top upregulated gene associated with cell wall metabolism is encoded for polygalacturonase (pectinase). Polylgalacturonase catalyzes the hydrolysis of alpha-1,4 glycosidic bonds in polygalacturonan (pectin) into monosaccharide subunits [193]. Pectin comprises a large portion of the primary cell wall in plants, although some studies suggest a minimal presence in wood tissue [194,195]. Pectin plays various roles within the cell wall, which include mediating cell-to-cell adhesion, facilitating ion transport, and contributing to porosity [196,197,198]. The variable sidechains of pectin can link to cellulose and hemicellulose, contributing to the overall mechanical strength of the cell wall [199]. The modification of pectin and the reorganization of cell wall components is a mechanism that can be utilized against heavy metal-induced stress [200,201]. Changes in the proportion of cell wall constituents may impart certain strategic advantages, depending on the structure of the cell wall and metabolic requirements [202]. Downregulation of pectinase in various plants also improved tolerance to multiple stressors by decreasing cell expansion and cell separation and increasing cell density [193,203,204]. Moy et al. found that polygalacturonate was among the most downregulated genes in copper-resistant trees in comparison to copper-susceptible trees, indicating an association with copper tolerance. In a previous study on *Pinus banksiana*, other cell wall synthesis-related genes were found to be among the top upregulated genes in copper RG compared to copper SG, indicating an association with copper resistance [99].

Information from the top upregulated genes and a previous study comparing copper RG with copper SG can be used to propose a model of copper resistance [99]. In comparison to nickel-treated plants, a lower proportion of genes were associated with the response to stress and signal transduction, indicating that *Pinus banksiana* may prioritize the direct detoxification of copper ions as opposed to counteracting symptoms caused by copper ions. NtPDR could export copper from the cytosol to the apoplast, at which point copper ions may interact with the cell wall [105,106]. HCT, CslE6, and MPG are involved with the reinforcement of the cell wall. In addition, the downregulation of beta-glucosidase and polygalacturonase in copper RG further supports this function [99]. Copper may then be sequestered in the cell wall and bind to ligands to decrease mobility and accumulation. Alternatively, cell wall synthesis could be driven by a higher amount of ROS mitigation in copper-resistant plants, as ROS may induce oxidative damage. Copper also seems to target photosynthesis machinery and inhibit photosynthesis. In copper-resistant plants, the early light-induced protein (ELIP) was upregulated, supporting the function of photosynthesis protection for the blue copper protein and the 22 KDA protein. Metabolic and protein-based assays should be performed to validate this proposed model.

### 3.4. Analysis of the Top Downregulated Genes for Copper-Resistant Plants Compared to Nickel-Resistant Genotypes Reveal Mechanisms That Are Associated with the Response to Excess Nickel

In nickel-resistant trees, the highest proportion of the top regulated gene expression was associated with the response to stress and signal transduction, indicating the implementation of distinct strategies in response to elevated levels of nickel. Stress response mechanisms may be used to alleviate symptoms of nickel toxicity, such as tissue damage, decreased photosynthesis, reduced plant biomass, and water loss [18,23,97]. Mechanisms that were associated with photosystem II and mitigating photodamage were more exclusive to copper than nickel. The differences in stress mechanisms could be explained by nickel and copper targeting different components of photosystem II. Excess copper inhibits plastoquinone QA and QB function, whereas excess nickel inhibits plastoquinone QB and the functionality of the oxygen-evolving complex [21,23,24]. Differences in ROS production may explain a higher proportion of genes allocated to cell wall metabolism in copper RG relative to nickel RG. Excess copper can increase the production of hydrogen peroxide and hydroxyl radicals from the Haber–Weiss reaction and Fenton-like reactions [29,30,31,205]. Comparatively, excess nickel may indirectly cause ROS stress by regulating the activity of antioxidative enzymes such as SOD, catalase, and peroxidase [32,33]. Due to nickel being less involved in plant physiology and being a cofactor for crucial enzymes, it is possible that excess nickel negatively impacts fewer areas of plant physiology and is thus relegated to fewer categories of biological responses. There is also a lack of identified transporters specific to nickel, which severely limits the physiological strategies that *Pinus banksiana* can utilize to detoxify nickel ions.

Among the top downregulated genes was a gene that encoded a UDP-glycosyltransferase (UGT), which catalyzes the transfer of glucose to abscisic acid [76]. The conversion of ABA to ABA-GE is a negative feedback mechanism that negatively regulates ABA synthesis and signaling [206,207]. As a phytohormone, ABA performs crosstalk with other stress-related hormones, modulating various aspects of stress mitigation such as transpiration rate, cell death, stomatal closure, photosynthesis, germination, and growth [208,209,210,211,212,213,214,215]. However, multiple instances of ABA application were found to decrease growth and cell replication in lieu of mediating physiological changes [215,216,217]. Application of ABA to cadmium-treated plants reduced root-to-shoot translocation and decreased IRT1, which is a nonspecific transporter that also mediates nickel uptake [218,219]. Reduction in ABA can thus be seen as a means to recover growth from nickel-mediated stress or indirectly influence nickel transport. In aluminum-stressed *Glycine max*, high UGT expression leads to the alteration of cell wall components, reducing callose, hemicellulose, glucose, and xylose [220]. In transgenic *Arabidopsis thaliana,* UGT interacted with metallothionein, facilitated hormone crosstalk, and alleviated oxidative stress in response to heat stress [221]. Overexpression of UGT in various species subjected to different abiotic stressors leads to decreases in auxin signaling, increases in seed germination, higher growth rates, and elevated flavonoid content [222,223,224,225]. The impact of UGT upregulation in nickel-treated *Pinus banksiana* may depend on various physiological parameters, necessitating future measurement and correlation.

A gene encoding TIFY was one of the most downregulated genes in copper RG in comparison to nickel RG. TIFY are transcription factors that constitutively suppress jasmonate-mediated signaling and are modulated by the ubiquitin-proteasome pathway [226,227]. Among the other jasmonate-related proteins identified in nickel RG in comparison to water, TIFY was the only protein to be highly upregulated in the nickel-resistant genotype compared to the copper-resistant genotype. TIFY responds to a variety of stressors and can be induced by the jasmonate and ABA signaling pathways [228,229]. In *Glycine max*, upregulated TIFY led to increases in peroxidase and catalase activity to counteract salt-induced stress [230]. In several plants subjected to various abiotic stressors, upregulated TIFY led to an increase in proline content and a reduction in MDA content, which is a toxic byproduct of lipid peroxidation [231,232,233,234]. Upregulation of TIFY in nickel could be a counteractive response to jasmonate production, as jasmonate is a phytohormone that reduces cell replication, photosynthesis, and overall growth in exchange for an elevated stress response [235,236,237]. Jasmonate inhibitors have been found to increase heavy metal tolerance by decreasing the impact of heavy metals on photosystem II and preventing chlorophyll loss [234,238,239]. Depending on the species, increased methyl jasmonate or jasmonic acid production may forego growth and development in exchange for increased heavy metal tolerance [240,241,242,243].

Among the top downregulated genes was a gene encoding for 1-aminocyclopropane-1-carboxylate synthase 7 (ACC synthase 7), which catalyzes the synthesis of 1-aminocyclopropane-1-carboxylate (ACC) [244]. ACC undergoes a rate-limiting oxidation reaction to produce ethylene, which is a phytohormone involved in stress processes, growth processes, and overall plant development [245,246]. Ethylene is a multifaceted phytohormone that may induce large alterations in stress tolerance when overexpressed or underexpressed. In *Arabidopsis thaliana*, ethylene can transcriptionally regulate the reduction in root growth and is also involved in photosynthesis recovery [247,248,249]. Ethylene mediates crosstalk with several hormones and signaling molecules, which include jasmonates, ABA, and nitrogen oxide [249,250,251]. In some instances, ethylene can indirectly alter antioxidative enzyme activity or influence glutathione-S-transferase synthesis [249,252,253,254]. In *Nelumbo nucifera*, the upregulation of ACC synthase increased both ACC and ethylene content, which led to higher sensitivity to cadmium-induced stress [254]. The increase in ethylene also caused the inhibition of catalase, ascorbate peroxidase, and glutathione reductase. In cadmium-treated *Nicotiana,* ethylene was decreased in the roots and increased in the shoots, resulting in higher shoot growth and photosynthesis recovery [255]. The endpoint phenotype for ethylene regulation seems to depend on the specific phytohormone crosstalk and the growth or photosynthetic requirements of the plant responding to stress [249,252]. Ethylene may also induce different outcomes based on the type of stressor involved [256]. Being the precursor to ethylene, the upregulation of ACC in nickel-resistant plants may indicate prioritization of photosynthesis recovery, ROS detoxification, and the stress response in exchange for tissue growth and development.

A top downregulated gene encodes for the dirigent protein, which is involved in the stress response, defense, and reorganization of the cell wall [257]. Dirigent proteins are involved in the production of lignins and lignans, which alter cell wall composition to counteract nickel-induced stress [258]. Dirigent proteins specifically mediate the phenoxy radical coupling of coniferyl alcohol to stereoselective pinoresinol [259]. Other genes involved in the synthesis of other cell wall components are not specific to nickel-resistant plants in comparison to copper-resistant plants. The upregulation of dirigent proteins and lignin may increase the mechanical strength and rigidity of the secondary cell wall [159,162,163]. The upregulation of lignin has also been correlated with increased cadmium deposition within the cell wall [260]. In *Arabidopsis thaliana,* DIR is involved in Casparian strip formation, which reinforces the impermeability of the cell wall [261,262]. Casparian strip formation may increase the blocking or reduction in heavy metal transport. In addition to being a reserve precursor for lignin formation, lignan is also a secondary metabolite that may be involved in the regulation of oxidative stress and flavonoid production [263,264,265]. Reduced lignan has been shown to strongly mediate ROS scavenging in the xylem, serving a strong protective function for the vasculature [264,266].

Another top downregulated gene is encoded for peroxidase, which is an enzyme involved in ROS scavenging [267]. Although not specific to any given stressor, peroxidase expression is a strong biomarker for oxidative stress, most notably in response to peroxide [268]. Peroxidase facilitates the electron transfer from hydrogen peroxide to a given substrate [267]. Peroxidase can quench radical peroxide and oxygen species, which leads to decreased levels of ROS [269]. In some trees, the upregulation of peroxidase is associated with increased lignin production, as peroxidase can oxidize monolignol radicals while simultaneously reducing peroxide [160]. The dual functionality of targeting oxidative stress and cell wall reinforcement could significantly contribute to heavy metal tolerance [270,271,272,273]. Peroxidase and peroxide levels may also negatively regulate auxin levels, which can impact plant growth and development [274]. Decreased auxin levels can impede apical meristem cell division, apical meristem differentiation, shoot branching, root branching, and overall plant growth [274,275,276,277].

A gene encoding for glyoxalase I was among the top downregulated genes. Glyoxalase I catalyzes the conversion of methylglyoxal (MG) to the inert compound S-D-lactoylgluathion, which is subsequently converted to D-lactate by glyoxalase II [278,279]. The conversion of MG to an inert compound serves dual roles by diminishing MG toxicity in plant tissue and generating D-lactate, which is a substrate involved in glucose metabolism [280]. As a byproduct of glycolysis, MG is involved in signaling pathways that modulate various areas of plant physiology. At low concentrations, MG can regulate ROS content, control stomatal closing, and activate potassium channels [281,282]. Excess nickel induces the overproduction of MG [283]. At higher concentrations, MG can reduce photosynthesis activity, impede root growth, decrease seed germination, cause chlorosis, and prevent ethylene synthesis [284,285,286,287,288,289]. Excess MG can also be detrimental to the function of critical enzymes and proteins, as MG facilitates the glycation of arginine and lysine [290]. The high expression of glyoxalase I in nickel RG could suggest a counteractive measure to reduce MG and the corresponding side effects causing cytotoxicity.

### 3.5. Analysis of the Top Upregulated Genes for Copper-Susceptible Plants Compared to Nickel-Susceptible Plants

Comparing the top regulated genes between copper-susceptible plants and nickel-susceptible plants may show tolerance mechanisms that are specific to either metal or reveal biomarkers indicative of copper or nickel vulnerability. Alternatively, DEGs in the susceptible genotype may not be associated with the response to heavy metals but could instead be associated with the management of cell death and tissue necrosis.

Among the top upregulated genes was a gene encoding inositol oxygenase, which catalyzes the oxidation of myto-inositol to D-glucuronic acid [291]. D-glucuronic acid is a critical component for hemicelluloses such as xylan and xyloses and can increase the level of binding and stability between components of the cell wall [292,293,294]. Upregulating the synthesis of UDP glucuronic acid can establish a pool of precursors to be utilized for building cell wall carbohydrates [295]. In *Arabidopsis thaliana*, mutants that did not produce D-glucuronic acid had altered hemicellulose, which resulted in a lower binding capacity and the accumulation of aluminum in the cell wall [296]. Many studies have shown the upregulation of inositol oxygenase to various stresses, with some studies demonstrating an improved stress response correlated to increased ROS mitigation activity [297,298].

A top upregulated gene encodes for a monosaccharide transporter, which facilitates the transport of glucose, xylose, and 3-O-methylglucose across different compartments [299,300]. In *Oryza sativa,* the expression of monosaccharide transporters in leaf cells, xylem cells, and sclerenchyma cells suggests a function that may pertain to cell wall reinforcement [299]. In response to stress, the upregulation of monosaccharide transporters may imply the accumulation of constituents in a pool to be utilized for either cell wall development or fulfilling metabolic requirements [299,300,301]. In many plants, increased expression of monosaccharide transporters led to increased production of protein, lipids, and overall development [302]. Fulfilling metabolic requirements could induce further growth and reproduction to counteract stress-induced damage [301,302].

A top downregulated gene encodes for a calmodulin-related protein, which binds calcium and is a critical component of the calmodulin signaling pathway [303]. As a calcium-binding sensor, the activity of calmodulin is dependent on cytosolic calcium levels, which are subject to alteration by a variety of different stressors [304]. In response to various stressors, calmodulin can facilitate crosstalk with signaling proteins, interact with secondary metabolites, interact with ion channels, and modulate enzymes to alter various aspects of physiology [305,306,307,308,309]. Many ion channels, phosphatases, and kinases are calmodulin-dependent, although the corresponding functions are too broad to attribute a specific function toward heavy metals [310,311,312,313,314]. Similar to other general stress responses, increased calmodulin is correlated with increased ROS scavenging [308]. In transgenic *Nicotiana tabacum*, overexpression of calmodulin conferred nickel tolerance, possibly by providing binding sites for nickel, which obstructs the channel or alters functionality [315]. Calmodulin has also been shown to nonspecifically facilitate the transport of other heavy metals, such as lead.

It should be pointed out that the validation of gene expression was performed by analyzing each treatment in triplicate and by applying a stringent pairwise comparison test. Additional validation by RT-qPCR can be considered for further study. Considering the scope of this study, such analysis will be time-consuming and not cost-effective.

## 4. Materials and Methods

### 4.1. Plant Growth and Treatment

The protocols for *Pinus banksiana* seedling growth, incubation, storage conditions, and metal treatment were similar to previous studies [316]. The College Boreal Plant Center in Sudbury, Ontario, provided six-month-old *Pinus banksiana* seedlings. These seedlings were transplanted into square plantar pots containing a 1:1 composition of sand with a mixture of 79% Sphagnum moss, 17% perlite, and 5% composted peat moss. The seedlings were incubated in a growth chamber for a month, receiving consistent water and fertilizer to ensure adequate growth. Plants were watered as needed and fertilized twice a week with equal amounts of nitrogen, phosphorus, and potassium (20–20–20). All treatments were applied in a randomized block design to account for variability among individual seedlings. Fifteen seedlings were given 1300 mg of copper sulfate per 1 kg of soil, administered through an aqueous solution in the soil. Another fifteen seedlings were treated in the same manner with 1600 mg of nickel sulfate per 1 kg of soil. The concentration of each treatment was representative of metal-contaminated sites in the Greater Sudbury Region [6]. Fifteen seedlings were given deionized water, which represented the negative control. Fifteen seedlings were treated with 1300 mg/kg of potassium sulfate as a salt control for the copper sulfate treatment. Fifteen seedlings were given 1600 mg/kg of potassium sulfate to represent the salt control for the nickel sulfate treatment.

After two weeks of incubation with continued monitoring, damages were recorded to identify the most resistant and susceptible seedlings using the 1 to 9 scale, where 1 to 3 represent resistant plants, 4 to 6 represent moderately resistant/susceptible plants, and 7 to 9 represent susceptible plants. Details of the rating scale are described in Table S1. Needles from resistant genotypes (RGs) with no symptoms and green needles from susceptible genotypes (SG) were harvested and wrapped in individual aluminum foils for RNA extraction. For longer-term storage, the needles were flash-frozen using liquid nitrogen and stored in a freezer at −80 °C.

### 4.2. Transcriptome Analysis of Pinus banksiana

Total RNA extraction, sequencing and transcriptome assembly, annotation of *Pinus banksiana* using BLAT matching, quantification of gene expression, and quality control (QC) analysis were described in Moy et al., 2023 [316]. Likewise, differential gene expression (DGE) analysis of pairwise comparisons and the identification of top upregulated and downregulated genes followed the procedure detailed in Moy et al., 2023 [316]. Three biological samples of each group, which include copper-resistant genotypes (RGs), Ni RGs, copper susceptible (SG), and Ni SG, along with water-treated samples and potassium sulfate-treated plants, were used for transcriptome analysis.

### 4.3. RNA Extraction

RNA extraction was conducted in accordance with the protocol outlined in the NORGEN BIOTEK Plant/Fungi Total RNA Purification Kit (https://norgenbiotek.com/product/plantfungi-total-rna-purification-kit, accessed on 1 September 2022). RNA quality was examined using agarose gel electrophoresis, and RNA quantification was performed using the Qubit™ RNA BR assay kit (Qubit RNA BR Assay Kit by Invitrogen Life technologies Corp., Eugene, OR, USA). The extracted RNA was stored at −80 °C in a freezer until use.

### 4.4. RNA Sequencing and De Novo Transcriptome Assembly

For every sample, messenger RNA was purified from the total RNA by using poly(dt) oligomers to target and bind to poly(A) tails. RNA fragmentation was performed to account for the size limitation of the sequencing platform. Reverse transcriptase was added to catalyze the reverse transcription of mRNA into cDNA. RNAse was added to prevent the unnecessary ligation or bonding of certain nucleotide pairs and strands. Second-strand synthesis was performed, followed by 3′ end ligation with adaptors and adenosine caps. Polymerase chain reaction (PCR) was used to synthesize and amplify the cDNA libraries of every sample. The cDNA libraries were sequenced using the Illumina sequencing platform at Seqmatic in Fremont, CA, USA. FASTQC files were generated from the cDNA libraries for each sample. The FASTQC program was used to assess the quality of the raw data from the files and generate characteristics for each sequence, which included average sequence length, percentage of guanine/cytosine content, the total percentage of deduplicated sequences, and sequences flagged as having poor quality. The Cutadapt program was used to delete adaptor sequences and low-quality bases from the raw sequence read data. The Bowtie2 algorithm in Trinity was used to map the RNA sequence raw reads to the Trinity transcript assembly, generating sequence alignment map (SAM) files, which were then converted to BAM (binary form of SAM) files. Transcript assembly was performed by inputting RNA sequence data from every sample into the TRINITY program, which quantified the number of genes based on the number of detected genes and corresponding isoforms.

### 4.5. BLAT Matching and Annotation of Pinus banksiana Genes

A two-way BLAST-like alignment tool (BLAT) was used to match and align the transcripts from the assembly to the reference genome of *Pinus taeda*. The characteristics of each transcript were provided, and they included the transcript ID, gene ID, and the corresponding log (E-value) for sequence similarity in relation to the reference genome. Other attributes that were acquired from the BLAT alignment included transcript sequence size, query sequence size, and the percentage of net match for each attribute. Every transcript was mapped to protein sequences in the UniProt database, generating corresponding UniProt IDs. Protein matches with the highest level of similarity were used to annotate genes and assign gene ontology information, such as the functions and cellular localization of the gene product.

### 4.6. Quantification of Gene Expression and Quality Control (QC) Analysis

The RNA-Seq by expectation–maximization (RSEM) abundance estimation method was used to quantify the expression level of each gene/transcript, as well as related isoforms. A quality control assessment was conducted for each read count to validate and assess the number of counts expressed for each gene. Raw reads were filtered and selected for counts of at least 1, 2, 10, 50, or 100. Genes with one read were considered noise. Genes with two or more counts were used as an estimate for the number of genes expressed. Genes with 10 or more counts were considered an adequate indication of the number of genes that had enough reads for downstream statistical analysis. For each treatment group, genes with counts per million (CPM) values of one or higher in at least two samples were included in the downstream analysis. Genes with a CPM value of less than one in at least two samples were considered unexpressed and removed. Normalization factors for raw counts were generated using a trimmed mean of M-values (TMM) from the edge R package (version 4.2) to normalize sample sizes and remove variations between samples.

The normalized read counts underwent log scale transformation using the voom method (log2 scale) from the R limma package. Boxplots of the transformed expression values were generated to show the mean distribution of every sample. Deviation from the mean distribution in a particular sample may indicate variations among experimental conditions, sample contamination, or the batch effect. Samples that deviated significantly from the mean distribution within the same objective group were excluded.

Multidimensional scaling plots were generated to show the amount of clustering for sample groups based on the leading log fold change (logFC) of normalized data. Groups of samples that deviated significantly from other groups of samples were considered differentially regulated. Samples that deviated significantly from the other samples within the same group were considered outliers and were excluded from the downstream analysis.

A heatmap was generated from the logFC of 5000 genes to show the visual relationship of differential gene expression between the samples. Samples that did not have a similar logFC pattern of gene expression as other samples within the same group were considered outliers and were excluded from the downstream analysis. The proportion of raw reads expressed by the top 100 upregulated and downregulated genes was also assessed in every sample to identify potential bottlenecking issues.

### 4.7. Differential Gene Expression (DGE) Analysis of Pairwise Comparisons

The cut-off for pairwise comparisons was calculated to be equivalent to 10 raw counts. A CPM of 0.413 was determined as the minimum threshold required for each pairwise comparison as calculated from the average of the total counts in all of the included samples. Genes that had a CPM higher than the cut-off in at least three samples were included in downstream analysis, whereas genes that did not fulfill these parameters were excluded. Out of 435,293 total genes, 150,739 genes were included in the differential gene expression analysis. The pairwise comparisons of transcript expression were performed at high-stringency and low-stringency cut-offs based on the false discovery rate and on *p* values (0.01) analyses, respectively. The pairwise comparisons were comprised of copper-resistant samples in comparison to nickel-resistant samples and copper-susceptible samples in comparison to nickel-susceptible samples. Differential gene expression expressed as logFC values was evaluated using the R limma package. To assess the interference of sulfate ions on the treatment regimen, pairwise comparisons of expressed genes were also conducted between each sample and the potassium sulfate control. Every gene for each pairwise comparison was annotated using Trinotate and Trinity. Gene ontology was performed by assigning GO terms and gene IDs from available databases to the set of genes for a given pairwise comparison. Genes that could not be annotated were filtered out of the set of annotated genes. Each gene set was run through a plant slim function using the Omicsbox (formally known as BLAST2GO) program. For each pairwise comparison, gene ontology charts were generated to functionally categorize biological processes, metabolic functions, and cellular components. For each functional category, sequences were distributed and organized by the NodeScore of each assigned GO term.

### 4.8. Analysis of Top Differentially Regulated Genes

The top 100 upregulated genes and downregulated genes were ranked for copper RG in comparison to nickel RG. Genes were ranked based on LogFC and the fulfillment of high-stringency parameters. This process was repeated for copper SG in comparison to nickel SG. UniProt annotation and a review of the current literature were conducted to characterize genes associated with copper detoxification or tolerance mechanisms. Genes associated with copper or nickel resistance were considered candidate genes for metal resistance. Gene ontology charts were generated to functionally categorize biological processes, metabolic functions, and cellular component localization for the top 100 regulated genes using the aforementioned process in DGE analysis. Charts comprised of the top 50 genes were provided for each pairwise comparison group.

## 5. Conclusions

The transcriptome analysis of *Pinus banksiana* treated with copper and nickel provided a deeper insight into the distinct genetic response of *Pinus banksiana* to excess Cu and Ni ions. At high stringency, there were 449 DEGs in copper RG in comparison to nickel RG, indicating a similar level of gene expression for the majority of the transcriptome. Out of the 449 genes, various terms in all three main categories were identified as differentially expressed. The decreased proportion of terms associated with DNA processes in the top DEGs suggests the presence of DNA methylation and regulation in the modulation of gene expression. There were not enough DEGs between copper SG and nickel SG to facilitate a GO categorical comparison. Annotation of the top upregulated genes in copper RG compared to nickel RG identified genes and mechanisms that were specific to copper and not to nickel. For biological processes, the biosynthetic process, response to chemicals, and carbohydrate metabolic process comprised the highest proportion of genes. NtPDR, AtHIPP10, and YSL1 were identified as genes associated with copper resistance. Genes encoding the blue copper protein and the 22 kDa Photosystem II protein were associated with the mitigation of photodamage and photosynthesis protection. Various genes related to cell wall metabolism were identified and included genes encoding for HCT, CslE6, MPG, and polygalacturonase. Annotation of the top downregulated genes in copper RG compared to nickel RG identified genes and mechanisms that were specific to nickel and not copper. For biological processes, the majority of gene expression was associated with the response to stress and signal transduction. Various regulatory and signaling-related genes associated with the stress response were identified, and they included UGT, TIFY, ACC, dirigent protein, peroxidase, and glyoxyalase I. Transcriptome analysis revealed the different strategies that are used by *Pinus banksiana* toward copper and nickel. The identified genes associated with copper resistance can be further researched and implemented in various industries. Additional research is needed to determine the specific functions of various signaling and stress response mechanisms in nickel-resistant plants.

## Figures and Tables

**Table 1:** Differentially expressed genes between copper and nickel-resistant genotypes.

Cut-Off	Standard (Two Fold and FDR 0.05)	Low Stringency (Two Fold and *p* Value 0.01)
Upregulated genes	269	3387
Downregulated genes	180	3815
Total genes	449	7202

FDR = False discovery rate.

**Table 2:** Differentially expressed genes between copper and nickel-susceptible genotypes.

Cut-Off	Standard (Two Fold and FDR 0.05)	Low Stringency (Two Fold and *p* Value 0.01)
Upregulated genes	6	2494
Downregulated genes	35	2626
Total genes	41	5120

FDR = False discovery rate.

**Table 3:** Top upregulated genes in copper resistant (RG) in comparison to nickel resistant (RG) in *Pinus banksiana*.

Rank	Gene ID	Cu Res1	Cu Res2	Cu Res3	Ni Res1	Ni Res2	Ni Res3	logFC	Adj. *p* Value	UniProt Description
0	TRINITY_DN2085_c0_g1	29.21	82.28	46.17	0.34	0.57	0.14	7.793	7.10E-05	Predicted Protein
1	TRINITY_DN1891_c0_g2	51.01	52.45	17.61	0.04	2.06	0.7	7.247	0.000114	Predicted Protein
2	TRINITY_DN8469_c0_g1	43.02	275.4	201.9	1.33	8.3	0.46	6.863	6.98E-05	Acid phosphatase 1, EC 3.1.3.2 (Apase-1(1))
3	TRINITY_DN1979_c0_g1	32.13	43.51	47.01	0.21	3.16	0.33	6.702	5.45E-06	Predicted Protein
4	TRINITY_DN4373_c0_g1	35.55	56.98	13.12	0.54	0.5	0.36	6.595	8.44E-05	Predicted Protein
5	TRINITY_DN5_c0_g1	18.7	16.84	12.27	0.1	0.8	0.23	6.538	0.000106363	Pleiotropic drug resistance protein 1 (NtPDR1)
6	TRINITY_DN5_c0_g1	18.7	16.84	12.27	0.1	0.8	0.23	6.538	0.000106	ABC transporter G family member 32, ABC transporter ABCG.32, AtABCG32 (Pleiotropic drug resistance protein 4) (Protein PERMEABLE CUTICLE 1)
7	TRINITY_DN5_c0_g1	18.7	16.84	12.27	0.1	0.8	0.23	6.538	0.000106	ABC transporter G family member 36, OsABCG36 (Pleiotropic drug resistance protein 9, OsPDR9)
8	TRINITY_DN5_c0_g1	18.7	16.84	12.27	0.1	0.8	0.23	6.538	0.000106	Pleiotropic drug resistance protein 1 (NpPDR1)
9	TRINITY_DN5_c0_g1	18.7	16.84	12.27	0.1	0.8	0.23	6.538	0.000106	ABC transporter G family member 44, OsABCG44 (Pleiotropic drug resistance protein 17, OsPDR17)
10	TRINITY_DN5_c0_g1	18.7	16.84	12.27	0.1	0.8	0.23	6.538	0.000106	ABC transporter G family member 31, OsABCG31 (Pleiotropic drug resistance protein 6, OsPDR6)
11	TRINITY_DN5_c0_g1	18.7	16.84	12.27	0.1	0.8	0.23	6.538	0.000106	ABC transporter G family member 34, OsABCG34 (Pleiotropic drug resistance protein 10, OsPDR10)
12	TRINITY_DN5742_c0_g1	25.47	76.4	14.17	0.6	1.07	0.41	6.162	5.17E-05	Predicted Protein
13	TRINITY_DN59895_c0_g1	107.34	136.22	54.47	0.8	4.56	2.36	6.155	1.45E-06	Predicted Protein
14	TRINITY_DN51306_c0_g1	32.05	61.43	35.69	0.53	0.7	1.91	6.153	1.48E-06	Predicted Protein
15	TRINITY_DN8170_c0_g1	50.21	123.33	300.25	1.81	1.13	8.48	6.150	5.72E-05	Predicted Protein
16	TRINITY_DN19606_c0_g1	31.77	21.11	20.22	0.5	1.66	0.29	5.866	4.22E-06	Granule-bound starch synthase 1, chloroplastic/amyloplastic, EC 2.4.1.242 (Granule-bound starch synthase I, GBSS-I)
17	TRINITY_DN6541_c0_g1	251.91	62.08	39.98	3.22	2.8	1.68	5.744	4.07E-05	Heavy metal-associated isoprenylated plant protein 20, AtHIP20, AtHIPP20
18	TRINITY_DN6541_c0_g1	251.91	62.08	39.98	3.22	2.8	1.68	5.744	4.07E-05	Heavy metal-associated isoprenylated plant protein 26, AtHIP26, AtHIPP26 (Farnesylated protein 6, AtFP6)
19	TRINITY_DN47729_c0_g2	7.89	24.87	20.63	0.46	0.23	0.87	5.668	0.000146	Predicted Protein
20	TRINITY_DN430_c0_g1	123.72	65.04	52.06	1.02	8.95	2.15	5.441	8.58E-06	Cytochrome P450 86A22, EC 1.14.14.129 (Long-chain acyl-CoA omega-monooxygenase)
21	TRINITY_DN1790_c0_g1	9.55	18.4	18.66	0.23	2.08	0.31	5.440	4.00E-05	Receptor-like protein kinase HSL1, EC 2.7.11.1 (Protein HAESA-LIKE1)
22	TRINITY_DN1072_c0_g1	97.21	115.92	41.8	4.4	9.29	0.62	5.334	6.56E-05	Predicted Protein
23	TRINITY_DN6055_c0_g1	18.79	22.98	21.77	0.56	1.18	0.81	5.330	5.78E-07	Predicted Protein
24	TRINITY_DN6813_c0_g1	235.35	117.07	35.45	1.7	10.19	3.65	5.309	6.74E-05	Predicted Protein
25	TRINITY_DN11710_c0_g2	150.88	92.2	20.39	1.17	7.66	2.13	5.304	0.000137	Predicted Protein
26	TRINITY_DN63319_c0_g1	28.39	21.29	22.62	0.58	1.05	1.41	5.255	8.92E-07	Predicted Protein
27	TRINITY_DN82353_c0_g1	13.94	23.06	17.36	0.87	0.98	0.45	5.156	2.89E-06	Predicted Protein
28	TRINITY_DN48473_c0_g1	56.5	107.55	80.8	1.68	5.59	4.91	5.085	9.27E-07	Predicted Protein
29	TRINITY_DN2130_c0_g1	22.32	44.82	44.69	2.2	1.09	1.68	5.050	4.23E-06	Predicted Protein
30	TRINITY_DN1441_c0_g2	340.37	453.06	121.8	13.03	19.89	9.5	4.943	5.01E-06	Predicted Protein

**Table 4:** Top downregulated genes in copper resistant (RG) in comparison to nickel resistant (RG) in *Pinus banksiana*.

Rank	Gene ID	Cu Res1	Cu Res2	Cu Res3	Ni Res1	Ni Res2	Ni Res3	logFC	Adj. *p* Value	UniProt Description
0	TRINITY_DN43547_c0_g1	0	0	0	24.15	39.01	23.93	−10.382	6.42E-05	Predicted Protein
1	TRINITY_DN1456_c0_g1	1.37	0	1.34	306.48	253.1	155.47	−9.527	0.000128719	Predicted Protein
2	TRINITY_DN2126_c0_g1	1.31	0.24	0.4	954.55	193.03	448.85	−9.030	4.26E-05	UDP-glycosyltransferase 75C1, Abscisic acid beta-glucosyltransferase, Indole-3-acetate beta-glucosyltransferase, SlUGT75C1, EC 2.4.1.121, EC 2.4.1.263
3	TRINITY_DN5965_c0_g1	0.28	0.12	1.02	159.21	70.92	203.21	−8.429	5.08E-05	Predicted Protein
4	TRINITY_DN5240_c1_g1	0.06	0.2	0.38	75.64	57.88	47.63	−7.894	0.000131675	Predicted Protein
5	TRINITY_DN2786_c0_g1	7.97	1.28	0.09	767.81	197.57	545.86	−7.817	0.000147002	Predicted Protein
6	TRINITY_DN7685_c0_g1	0.35	0.17	0.18	69.15	75.78	88.19	−7.718	1.68E-05	Predicted Protein
7	TRINITY_DN3685_c0_g2	7.54	1.2	0.09	524.13	169.45	298.36	−7.315	0.000146393	Copia protein (Gag-int-pol protein) [Cleaved into: Copia VLP protein; Copia protease, EC 3.4.23.-]
8	TRINITY_DN2463_c0_g1	5.21	0.97	0.68	301.76	196.16	568.86	−7.021	8.44E-06	Predicted Protein
9	TRINITY_DN2040_c0_g1	28.51	6	12.87	3943.91	1043.09	3644.25	−6.950	1.04E-05	Predicted Protein
10	TRINITY_DN5795_c0_g1	13.6	5.09	0.4	753.52	420.03	412.9	−6.580	5.36E-05	Predicted Protein
11	TRINITY_DN735_c0_g1	5.88	1.92	0.26	401.38	124.53	198.21	−6.422	9.90E-05	Predicted Protein
12	TRINITY_DN41085_c0_g1	1.46	0.55	8.2	230.93	100.32	461.71	−6.396	8.67E-05	Predicted Protein
13	TRINITY_DN5240_c0_g1	0.4	0.48	1.48	86.62	78.96	59.64	−6.375	1.11E-06	Protein TIFY 10b, OsTIFY10b (Jasmonate ZIM domain-containing protein 7, OsJAZ7) (OsJAZ6)
14	TRINITY_DN17_c0_g2	0.65	0.15	0.58	50.63	58.32	26.97	−6.307	1.89E-05	RING-H2 finger protein ATL60, EC 2.3.2.27 (RING-type E3 ubiquitin transferase ATL60)
15	TRINITY_DN800_c1_g1	2.53	0.34	0.33	127.84	44.55	121.22	−6.264	0.000103509	Predicted Protein
16	TRINITY_DN1755_c0_g1	0.73	0.19	0.22	37.36	42.04	37.53	−6.143	1.54E-05	Predicted Protein
17	TRINITY_DN1067_c0_g1	0.21	0.63	2.66	74.75	51.13	62.5	−6.138	1.50E-05	Predicted Protein
18	TRINITY_DN4963_c0_g1	1.2	1.08	0.26	107.91	63.25	101.25	−6.114	6.05E-06	Predicted Protein
19	TRINITY_DN3889_c0_g1	8.77	0.85	2.51	414.11	167.54	300.23	−6.071	3.22E-05	Predicted Protein
20	TRINITY_DN6289_c0_g1	0.34	0.08	0.38	23.79	21.01	21.41	−5.988	0.000135072	Predicted Protein
21	TRINITY_DN6111_c0_g1	0.47	0.11	0.98	29.31	29.12	29.02	−5.932	1.49E-05	Predicted Protein
22	TRINITY_DN4477_c0_g1	1.32	1.08	0.77	67.92	163.48	92.08	−5.924	1.74E-07	Predicted Protein
23	TRINITY_DN71807_c0_g1	0.75	0.09	1.03	29.61	23.8	49.04	−5.903	8.02E-05	Predicted Protein
24	TRINITY_DN971_c0_g1	0.32	0.18	3.12	40.71	30.9	35.98	−5.891	3.15E-05	1-aminocyclopropane-1-carboxylate synthase 7, ACC synthase 7, EC 4.4.1.14 (S-adenosyl-L-methionine methylthioadenosine-lyase 7)
25	TRINITY_DN971_c0_g1	0.32	0.18	3.12	40.71	30.9	35.98	−5.891	3.15E-05	1-aminocyclopropane-1-carboxylate synthase, ACC synthase, EC 1.4.-.-, EC 4.4.1.14 (S-adenosyl-L-methionine methylthioadenosine-lyase)
26	TRINITY_DN971_c0_g1	0.32	0.18	3.12	40.71	30.9	35.98	−5.891	3.15E-05	1-aminocyclopropane-1-carboxylate synthase 6, ACC synthase 6, EC 4.4.1.14 (S-adenosyl-L-methionine methylthioadenosine-lyase 6)
27	TRINITY_DN971_c0_g1	0.32	0.18	3.12	40.71	30.9	35.98	−5.891	3.15E-05	1-aminocyclopropane-1-carboxylate synthase 3, ACC synthase 3, EC 4.4.1.14 (Le-ACS3, ACS-3) (S-adenosyl-L-methionine methylthioadenosine-lyase 3)
28	TRINITY_DN971_c0_g1	0.32	0.18	3.12	40.71	30.9	35.98	−5.891	3.15E-05	1-aminocyclopropane-1-carboxylate synthase 1, ACC synthase 1, EC 4.4.1.14 (S-adenosyl-L-methionine methylthioadenosine-lyase)
29	TRINITY_DN756_c0_g1	7.06	0.88	8.25	578.57	195.91	235.86	−5.769	7.82E-05	Dirigent protein 21, AtDIR21
30	TRINITY_DN6061_c0_g1	3.75	2.9	7.44	355.22	178.84	637.66	−5.730	1.01E-05	Predicted Protein

**Table 5:** Top upregulated genes in copper susceptible (SG) in comparison to nickel susceptible (SG) in *Pinus banksiana*.

Rank	Gene ID	Cu Sus 1	Cu Sus 2	Cu Sus 3	Ni Sus 1	Ni Sus 2	Ni Sus 3	logFC	Adj. *p* Value	UniProt Description
0	TRINITY_DN3441_c0_g1	161.51	141.19	99.42	14.73	4.54	4.16	4.327038	4.74E-06	Predicted Protein
1	TRINITY_DN29629_c0_g1	130.48	102.59	87.52	8.98	4.39	3.79	4.292084	1.72E-06	Predicted Protein
2	TRINITY_DN25822_c0_g1	52.29	67.97	55.74	7.15	2.61	2.46	3.959541	7.45E-06	Sugar transport protein MST3 (Monosaccharide transporter 3, OsMST3) (Sugar:proton symporter MST3)
3	TRINITY_DN324_c0_g1	122.98	261.98	186.02	18.74	13.27	19.44	3.330385	5.86E-06	Inositol oxygenase 1, EC 1.13.99.1 (Myo-inositol oxygenase 1, AtMIOX1, MI oxygenase 1)
4	TRINITY_DN324_c0_g1	122.98	261.98	186.02	18.74	13.27	19.44	3.330385	5.86E-06	Inositol oxygenase 5, EC 1.13.99.1 (Myo-inositol oxygenase 5, AtMIOX5, MI oxygenase 5)
5	TRINITY_DN324_c0_g1	122.98	261.98	186.02	18.74	13.27	19.44	3.330385	5.86E-06	Probable inositol oxygenase, EC 1.13.99.1 (Myo-inositol oxygenase, MI oxygenase)
6	TRINITY_DN324_c0_g1	122.98	261.98	186.02	18.74	13.27	19.44	3.330385	5.86E-06	Inositol oxygenase 4, EC 1.13.99.1 (Myo-inositol oxygenase 4, AtMIOX4, MI oxygenase 4)
7	TRINITY_DN13749_c0_g1	87.25	112.1	72.4	18.01	11.02	12.71	2.669987	5.62E-06	Predicted Protein
8	TRINITY_DN1743_c0_g1	398.17	530.25	356.13	126.44	202.71	141.62	1.416768	5.38E-06	G-type lectin S-receptor-like serine/threonine-protein kinase At5g24080, EC 2.7.11.1

**Table 6:** Top downregulated genes in copper susceptible (SG) in comparison to nickel susceptible (SG) in *Pinus banksiana*.

Rank	Gene ID	Cu Sus1	Cu Sus2	Cu Sus3	Ni Sus1	Ni Sus2	Ni Sus3	logFC	Adj. *p* Value	UniProt Description
0	TRINITY_DN343_c0_g1	1.96	1.42	0.92	54.19	45.61	75.54	−5.26455	7.48E-06	Probable disease resistance protein At4g33300
1	TRINITY_DN1105_c0_g1	3.27	4.87	2.07	76.5	72.45	109.99	−4.74021	8.13E-07	Probable calcium-binding protein CML24 (Calmodulin-like protein 24)
2	TRINITY_DN1140_c1_g1	4.64	8.22	3.13	95.64	64.13	116.13	−4.21256	7.05E-06	Heavy metal-associated isoprenylated plant protein 39, AtHIP39
3	TRINITY_DN9069_c0_g1	3.08	3.94	1.43	39	35.11	55.72	−4.0072	7.49E-06	Predicted Protein
4	TRINITY_DN26455_c0_g1	5.55	5.56	3.67	47.82	81.22	105.73	−3.96197	1.86E-06	Predicted Protein
5	TRINITY_DN709_c0_g1	3.48	4.83	1.97	36.9	41.46	63.36	−3.84112	4.28E-06	Predicted Protein
6	TRINITY_DN24318_c0_g1	4.75	2.79	4.67	40.3	50.05	80.73	−3.79817	8.01E-06	Predicted Protein
7	TRINITY_DN2118_c0_g1	5.31	19.73	5.94	91.04	111.37	142.16	−3.79689	2.20E-06	Predicted Protein
8	TRINITY_DN66111_c1_g1	14.78	24.19	24.59	172.94	234.1	371.3	−3.66779	1.60E-06	Predicted Protein
9	TRINITY_DN9317_c0_g1	9.85	29.45	5.37	142.89	125.63	137.64	−3.56924	7.93E-06	Predicted Protein
10	TRINITY_DN25789_c0_g1	5.35	15.72	3.93	67.56	76.93	92.73	−3.54421	3.52E-06	Predicted Protein
11	TRINITY_DN2751_c0_g1	12.73	8.43	4.46	77.66	76.97	116.69	−3.4682	4.60E-06	Predicted Protein
12	TRINITY_DN1481_c0_g1	31.61	46.43	12.53	248.24	432.11	226.95	−3.44461	5.91E-06	Predicted Protein
13	TRINITY_DN37281_c0_g1	6.17	13.07	6.21	74.35	73.03	101.82	−3.42972	1.93E-06	Predicted Protein
14	TRINITY_DN11462_c0_g1	15.98	31.57	16.82	122.24	278.4	208.28	−3.29128	1.68E-06	Predicted Protein
15	TRINITY_DN3306_c0_g2	18.25	34.56	13.19	154.79	169.1	263.27	−3.27608	1.59E-06	Calcium-binding protein KIC (KCBP-interacting calcium-binding protein)
16	TRINITY_DN4522_c0_g1	12.58	21.21	9.5	81.86	98.59	152.54	−3.01301	3.71E-06	Predicted Protein
17	TRINITY_DN729_c0_g1	85.61	37.04	35.87	381.71	394.83	348.76	−2.92258	4.38E-06	Alcohol dehydrogenase 2, EC 1.1.1.1
18	TRINITY_DN729_c0_g1	85.61	37.04	35.87	381.71	394.83	348.76	−2.92258	4.38E-06	Alcohol dehydrogenase 1, EC 1.1.1.1 (ADH slow-allele)
19	TRINITY_DN41085_c0_g1	50.47	50.02	52.9	257.45	297.21	485.74	−2.76064	2.45E-06	Predicted Protein
20	TRINITY_DN3066_c1_g1	13.73	15.11	13.46	55.99	116.42	118.98	−2.74976	6.97E-06	Predicted Protein
21	TRINITY_DN2515_c0_g1	42.66	64	22.61	203.34	269.87	276.58	−2.66249	6.45E-07	Probable disease resistance protein At5g04720
22	TRINITY_DN1690_c0_g1	82.49	125.29	74.33	334.26	557.98	584.67	−2.43231	2.73E-07	Predicted Protein
23	TRINITY_DN1454_c0_g1	36.11	69.11	23.97	190.96	205.3	220.09	−2.42405	3.20E-06	Probable disease resistance protein At4g33300
24	TRINITY_DN1454_c0_g1	36.11	69.11	23.97	190.96	205.3	220.09	−2.42405	3.20E-06	Disease resistance protein ADR1 (Activated disease resistance protein 1)
25	TRINITY_DN187_c0_g2	34.07	59.55	27.12	157.12	214.46	204.87	−2.36432	8.35E-07	Probable disease resistance protein At4g33300
26	TRINITY_DN187_c0_g2	34.07	59.55	27.12	157.12	214.46	204.87	−2.36432	8.35E-07	Probable RNA-binding protein 19 (RNA-binding motif protein 19)
27	TRINITY_DN2581_c0_g1	73.03	104.45	74.62	307.26	360.65	549.82	−2.30639	2.39E-06	Ubiquitin-40S ribosomal protein S27a-1 [Cleaved into: Ubiquitin; 40S ribosomal protein S27a-1 ]
28	TRINITY_DN829_c0_g1	36.79	52.72	34.25	136.33	187.57	265.96	−2.27764	4.73E-06	Predicted Protein
29	TRINITY_DN871_c0_g1	77.17	92.43	46.57	254.71	317.44	342.34	−2.14567	5.09E-07	Lipase-like PAD4, EC 2.3.1.- (Protein ENHANCED DISEASE SUSCEPTIBILITY 9) (Protein PHYTOALEXIN DEFICIENT 4, AtPAD4)
30	TRINITY_DN2157_c0_g1	68.38	76.54	36.2	215.2	233.04	270.04	−2.06397	3.08E-06	Predicted Protein

## Data Availability

We have deposited data in the following depository: Repository/DataBank Accession: NCBI GenBank; BioProject Accession Number PRJNA962116; Databank URL: http://www.ncbi.nlm.nih.gov/genbank. Repository/DataBank Accession: EmbL; BioProject Accession Number PRJNA962116; Databank URL: http://www.ebi.ac.uk/ena.

## Supplementary Materials

The following supporting information can be downloaded at: https://www.mdpi.com/article/10.3390/plants13071042/s1, Figure S1: Percent distribution of differentially expressed genes (DEGs) for cell compartment localization when copper resistant (RG) and nickel resistant (RG) were compared; Table S1: Damage rating scale and plant classification based on reaction to nickel and copper treatments; Table S2: Top 108 upregulated genes in copper RG in comparison to Nickel RG in Pinus banksiana; Table S3: Top 112 downregulated genes in copper RG in comparison to Nickel RG in *Pinus banksiana*; Table S4: Top 40 downregulated genes in copper SG in comparison to nickel SG in *Pinus banksiana*

## References

[1] Tchounwou P.B., Yedjou C.G., Patlolla A.K., Sutton D.J., Luch A. (2012). Heavy Metal Toxicity and the Environment. Molecular, Clinical and Environmental Toxicology.

[2] DeForest D.K., Brix K.V., Adams W.J. (2007). Assessing Metal Bioaccumulation in Aquatic Environments: The Inverse Relationship between Bioaccumulation Factors, Trophic Transfer Factors and Exposure Concentration. Aquat. Toxicol. Amst. Neth..

[3] Jara-Marini M.E., Soto-Jiménez M.F., Páez-Osuna F. (2009). Trophic Relationships and Transference of Cadmium, Copper, Lead and Zinc in a Subtropical Coastal Lagoon Food Web from SE Gulf of California. Chemosphere.

[4] Jewiss T. The Mining History of the Sudbury Area|Earth Sciences Museum. https://uwaterloo.ca/earth-sciences-museum/resources/mining-canada/mining-history-sudbury-area.

[5] Kramer D.M., Holness D.L., Haynes E., McMillan K., Berriault C., Kalenge S., Lightfoot N. (2017). From Awareness to Action: Sudbury, Mining and Occupational Disease in a Time of Change. Work.

[6] Nkongolo K.K., Spiers G., Beckett P., Narendrula R., Theriault G., Tran A., Kalubi K.N. (2013). Long-Term Effects of Liming on Soil Chemistry in Stable and Eroded Upland Areas in a Mining Region. Water. Air. Soil Pollut..

[7] Schindler M. (2014). A Mineralogical and Geochemical Study of Slag from the Historical O’donnell Roast Yards, Sudbury, Ontario, Canada. Can. Mineral..

[8] Andreazza R., Okeke B.C., Pieniz S., Bento F.M., Camargo F.A.O. (2013). Biosorption and Bioreduction of Copper from Different Copper Compounds in Aqueous Solution. Biol. Trace Elem. Res..

[9] Yusuf M., Fariduddin Q., Hayat S., Ahmad A. (2011). Nickel: An Overview of Uptake, Essentiality and Toxicity in Plants. Bull. Environ. Contam. Toxicol..

[10] Höhner R., Pribil M., Herbstová M., Lopez L.S., Kunz H.-H., Li M., Wood M., Svoboda V., Puthiyaveetil S., Leister D. (2020). Plastocyanin Is the Long-Range Electron Carrier between Photosystem II and Photosystem I in Plants. Proc. Natl. Acad. Sci. USA.

[11] Cao J., Li X., Lv Y., Ding L. (2015). Comparative Analysis of the Phytocyanin Gene Family in 10 Plant Species: A Focus on Zea Mays. Front. Plant Sci..

[12] Mansilla N., Welchen E., Gonzalez D.H. (2019). Arabidopsis SCO Proteins Oppositely Influence Cytochrome c Oxidase Levels and Gene Expression during Salinity Stress. Plant Cell Physiol..

[13] Barcelos J.P.Q., Reis H.P.G., Godoy C.V., Gratão P.L., Furlani Junior E., Putti F.F., Campos M., Reis A.R. (2018). Impact of Foliar Nickel Application on Urease Activity, Antioxidant Metabolism and Control of Powdery Mildew (*Microsphaera diffusa*) in Soybean Plants. Plant Pathol..

[14] Urra M., Buezo J., Royo B., Cornejo A., López-Gómez P., Cerdán D., Esteban R., Martínez-Merino V., Gogorcena Y., Tavladoraki P. (2022). The Importance of the Urea Cycle and Its Relationships to Polyamine Metabolism during Ammonium Stress in *Medicago truncatula*. J. Exp. Bot..

[15] Lin S.-L., Wu L. (1994). Effects of Copper Concentration on Mineral Nutrient Uptake and Copper Accumulation in Protein of Copper-Tolerant and Nontolerant *Lotus purshianus* L.. Ecotoxicol. Environ. Saf..

[16] Ivanov Y.V., Kartashov A.V., Ivanova A.I., Savochkin Y.V., Kuznetsov V.V. (2016). Effects of Copper Deficiency and Copper Toxicity on Organogenesis and Some Physiological and Biochemical Responses of Scots Pine (*Pinus Sylvestris* L.) Seedlings Grown in Hydroculture. Environ. Sci. Pollut. Res..

[17] Martins L.L., Mourato M.P. (2006). Effect of Excess Copper on Tomato Plants: Growth Parameters, Enzyme Activities, Chlorophyll, and Mineral Content. J. Plant Nutr..

[18] Ghasemi R., Ghaderian S.M., Krämer U. (2009). Interference of Nickel with Copper and Iron Homeostasis Contributes to Metal Toxicity Symptoms in the Nickel Hyperaccumulator Plant Alyssum Inflatum. New Phytol..

[19] Rubio M.I., Escrig I., Martínez-Cortina C., López-Benet F.J., Sanz A. (1994). Cadmium and Nickel Accumulation in Rice Plants. Effects on Mineral Nutrition and Possible Interactions of Abscisic and Gibberellic Acids. Plant Growth Regul..

[20] Yang X., Baligar V.C., Martens D.C., Clark R.B. (1996). Plant Tolerance to Nickel Toxicity: II Nickel Effects on Influx and Transport of Mineral Nutrients in Four Plant Species. J. Plant Nutr..

[21] Jegerschoeld C., Arellano J.B., Schroeder W.P., van Kan P.J.M., Baron M., Styring S. (1995). Copper(II) Inhibition of Electron Transfer through Photosystem II Studied by EPR Spectroscopy. Biochemistry.

[22] Mohanty N., Vass I., Demeter S. (1989). Copper Toxicity Affects Photosystem II Electron Transport at the Secondary Quinone Acceptor, QB1. Plant Physiol..

[23] Mohanty N., Vass I., Demeter S. (1989). Impairment of Photosystem 2 Activity at the Level of Secondary Quinone Electron Acceptor in Chloroplasts Treated with Cobalt, Nickel and Zinc Ions. Physiol. Plant..

[24] Boisvert S., Joly D., Leclerc S., Govindachary S., Harnois J., Carpentier R. (2007). Inhibition of the Oxygen-Evolving Complex of Photosystem II and Depletion of Extrinsic Polypeptides by Nickel. BioMetals.

[25] Küpper H., Küpper F., Spiller M. (1996). Environmental Relevance of Heavy Metal-Substituted Chlorophylls Using the Example of Water Plants. J. Exp. Bot..

[26] Batool S. (2018). Effect of Nickel Toxicity on Growth, Photosynthetic Pigments and Dry Matter Yield of *Cicer Arietinum* L. Varieties. Asian J. Agri. Biol..

[27] Baran U., Ekmekçi Y. (2021). Correction to: Physiological, Photochemical, and Antioxidant Responses of Wild and Cultivated Carthamus Species Exposed to Nickel Toxicity and Evaluation of Their Usage Potential in Phytoremediation. Environ. Sci. Pollut. Res..

[28] Küpper H., Küpper F., Spiller M. (1998). In Situ Detection of Heavy Metal Substituted Chlorophylls in Water Plants. Photosynth. Res..

[29] Opdenakker K., Remans T., Keunen E., Vangronsveld J., Cuypers A. (2012). Exposure of Arabidopsis Thaliana to Cd or Cu Excess Leads to Oxidative Stress Mediated Alterations in MAPKinase Transcript Levels. Environ. Exp. Bot..

[30] Wang L., Yang L., Yang F., Li X., Song Y., Wang X., Hu X. (2010). Involvements of H_2_O_2_ and Metallothionein in NO-Mediated Tomato Tolerance to Copper Toxicity. J. Plant Physiol..

[31] Kucková L., Jomová K., Švorcová A., Valko M., Segľa P., Moncoľ J., Kožíšek J. (2015). Synthesis, Crystal Structure, Spectroscopic Properties and Potential Biological Activities of Salicylate–Neocuproine Ternary Copper(II) Complexes. Molecules.

[32] Gajewska E., Skłodowska M. (2006). Effect of Nickel on ROS Content and Antioxidative Enzyme Activities in Wheat Leaves. BioMetals.

[33] Baccouch S., Chaoui A., El Ferjani E. (2001). Nickel Toxicity Induces Oxidative Damage in *Zea mays* Roots. J. Plant Nutr..

[34] Panou-Filotheou H. (2001). Effects of Copper Toxicity on Leaves of Oregano (*Origanum Vulgare* Subsp. Hirtum). Ann. Bot..

[35] Molas J. (1998). Changes in Morphological and Anatomical Structure of Cabbage (*Brassica Oleracea* L.) Outer Leaves and in Ultrastructure of Their Chloroplasts Caused by an in Vitro Excess of Nickel. Photosynthetica.

[36] Ahmad M.S.A., Riffat A., Hussain M., Hameed M., Alvi A.K. (2023). Toxicity and Tolerance of Nickel in Sunflower (*Helianthus Annuus* L.). Environ. Sci. Pollut. Res..

[37] Marques D.M., da Silva A.B., Mantovani J.R., Magalhães P.C., de Souza T.C. (2019). Root Morphology and Leaf Gas Exchange in *Peltophorum Dubium* (Spreng.) Taub. (Caesalpinioideae) Exposed to Copper-Induced Toxicity. S. Afr. J. Bot..

[38] Kulikova A.L., Kuznetsova N.A., Kholodova V.P. (2011). Effect of Copper Excess in Environment on Soybean Root Viability and Morphology. Russ. J. Plant Physiol..

[39] Sancenón V., Puig S., Mateu-Andrés I., Dorcey E., Thiele D.J., Peñarrubia L. (2004). The Arabidopsis Copper Transporter COPT1 Functions in Root Elongation and Pollen Development*. J. Biol. Chem..

[40] Liu X.S., Feng S.J., Zhang B.Q., Wang M.Q., Cao H.W., Rono J.K., Chen X., Yang Z.M. (2019). OsZIP1 Functions as a Metal Efflux Transporter Limiting Excess Zinc, Copper and Cadmium Accumulation in Rice. BMC Plant Biol..

[41] del Pozo T., Cambiazo V., González M. (2010). Gene Expression Profiling Analysis of Copper Homeostasis in *Arabidopsis Thaliana*. Biochem. Biophys. Res. Commun..

[42] Wintz H., Fox T., Wu Y.-Y., Feng V., Chen W., Chang H.-S., Zhu T., Vulpe C. (2003). Expression Profiles of *Arabidopsis Thaliana* in Mineral Deficiencies Reveal Novel Transporters Involved in Metal Homeostasis. J. Biol. Chem..

[43] Nishida S., Tanikawa R., Ishida S., Yoshida J., Mizuno T., Nakanishi H., Furuta N. (2020). Elevated Expression of Vacuolar Nickel Transporter Gene IREG2 Is Associated With Reduced Root-to-Shoot Nickel Translocation in *Noccaea Japonica*. Front. Plant Sci..

[44] Kozak K., Papierniak-Wygladala A., Palusińska M., Barabasz A., Antosiewicz D.M. (2022). Regulation and Function of Metal Uptake Transporter NtNRAMP3 in Tobacco. Front. Plant Sci..

[45] Shaheen S., Ahmad R., Mahmood Q., Pervez A., Maroof Shah M., Hafeez F. (2019). Gene Expression and Biochemical Response of Giant Reed under Ni and Cu Stress. Int. J. Phytoremediation.

[46] Andrés-Colás N., Sancenón V., Rodríguez-Navarro S., Mayo S., Thiele D.J., Ecker J.R., Puig S., Peñarrubia L. (2006). The Arabidopsis Heavy Metal P-Type ATPase HMA5 Interacts with Metallochaperones and Functions in Copper Detoxification of Roots. Plant J..

[47] Deng F., Yamaji N., Xia J., Ma J.F. (2013). A Member of the Heavy Metal P-Type ATPase OsHMA5 Is Involved in Xylem Loading of Copper in Rice. Plant Physiol..

[48] Lee S., Kim Y.-Y., Lee Y., An G. (2007). Rice P1B-Type Heavy-Metal ATPase, OsHMA9, Is a Metal Efflux Protein. Plant Physiol..

[49] Seigneurin-Berny D., Gravot A., Auroy P., Mazard C., Kraut A., Finazzi G., Grunwald D., Rappaport F., Vavasseur A., Joyard J. (2006). HMA1, a New Cu-ATPase of the Chloro Plast Envelope, Is Essential for Growth under Adverse Light Conditions. J. Biol. Chem..

[50] Boutigny S., Sautron E., Finazzi G., Rivasseau C., Frelet-Barrand A., Pilon M., Rolland N., Seigneurin-Berny D. (2014). HMA1 and PAA1, Two Chloroplast-Envelope PIB-ATPases, Play Distinct Roles in Chloroplast Copper Homeostasis. J. Exp. Bot..

[51] Sautron E., Mayerhofer H., Giustini C., Pro D., Crouzy S., Ravaud S., Pebay-Peyroula E., Rolland N., Catty P., Seigneurin-Berny D. (2015). HMA6 and HMA8 Are Two Chloroplast Cu+-ATPases with Different Enzymatic Properties. Biosci. Rep..

[52] Shikanai T., Müller-Moulé P., Munekage Y., Niyogi K.K., Pilon M. (2003). PAA1, a P-Type ATPase of Arabidopsis, Functions in Copper Transport in Chloroplasts. Plant Cell.

[53] Mayerhofer H., Sautron E., Rolland N., Catty P., Seigneurin-Berny D., Pebay-Peyroula E., Ravaud S. (2016). Structural Insights into the Nucleotide-Binding Domains of the P1B-Type ATPases HMA6 and HMA8 from Arabidopsis Thaliana. PLoS ONE.

[54] Tapken W., Ravet K., Pilon M. (2012). Plastocyanin Controls the Stabilization of the Thylakoid Cu-Transporting P-Type ATPase PAA2/HMA8 in Response to Low Copper in Arabidopsis. J. Biol. Chem..

[55] Tapken W., Ravet K., Shahbaz M., Pilon M. (2015). Regulation of Cu Delivery to Chloroplast Proteins. Plant Signal. Behav..

[56] Li W., Lacey R.F., Ye Y., Lu J., Yeh K.-C., Xiao Y., Li L., Wen C.-K., Binder B.M., Zhao Y. (2017). Triplin, a Small Molecule, Reveals Copper Ion Transport in Ethylene Signaling from ATX1 to RAN1. PLoS Genet..

[57] Mira H., Martínez-García F., Peñarrubia L. (2001). Evidence for the Plant-Specific Intercellular Transport of the Arabidopsis Copper Chaperone CCH. Plant J..

[58] Mari S., Gendre D., Pianelli K., Ouerdane L., Lobinski R., Briat J.-F., Lebrun M., Czernic P. (2006). Root-to-Shoot Long-Distance Circulation of Nicotianamine and Nicotianamine-Nickel Chelates in the Metal Hyperaccumulator *Thlaspi Caerulescens*. J. Exp. Bot..

[59] Ingle R.A., Mugford S.T., Rees J.D., Campbell M.M., Smith J.A.C. (2005). Constitutively High Expression of the Histidine Biosynthetic Pathway Contributes to Nickel Tolerance in Hyperaccumulator Plants. Plant Cell.

[60] Irtelli B., Petrucci W.A., Navari-Izzo F. (2008). Nicotianamine and Histidine/Proline Are, Respectively, the Most Important Copper Chelators in Xylem Sap of *Brassica Carinata* under Conditions of Copper Deficiency and Excess. J. Exp. Bot..

[61] Guo W.-J., Bundithya W., Goldsbrough P.B. (2003). Characterization of the Arabidopsis Metallothionein Gene Family: Tissue-Specific Expression and Induction during Senescence and in Response to Copper. New Phytol..

[62] Pich A., Scholz G. (1996). Translocation of Copper and Other Micronutrients in Tomato Plants (*Lycopersicon esculentum* Mill.): Nicotianamine-Stimulated Copper Transport in the Xylem. J. Exp. Bot..

[63] Curie C., Cassin G., Couch D., Divol F., Higuchi K., Le Jean M., Misson J., Schikora A., Czernic P., Mari S. (2009). Metal Movement within the Plant: Contribution of Nicotianamine and Yellow Stripe 1-like Transporters. Ann. Bot..

[64] Gendre D., Czernic P., Conéjéro G., Pianelli K., Briat J.-F., Lebrun M., Mari S. (2006). TcYSL3, a Member of the YSL Gene Family from the Hyper-Accumulator *Thlaspi Caerulescens*, Encodes a Nicotianamine-Ni/Fe Transporter. Plant J..

[65] Li L., Hou M., Cao L., Xia Y., Shen Z., Hu Z. (2018). Glutathione S-Transferases Modulate Cu Tolerance in Oryza Sativa. Environ. Exp. Bot..

[66] Li G.-Z., Zheng Y.-X., Chen S.-J., Liu J., Wang P.-F., Wang Y.-H., Guo T.-C., Kang G.-Z. (2021). TaWRKY74 Participates Copper Tolerance through Regulation of TaGST1 Expression and GSH Content in Wheat. Ecotoxicol. Environ. Saf..

[67] Arduini I., Godbold D.L., Onnis A. (1996). Cadmium and Copper Uptake and Distribution in Mediterranean Tree Seedlings. Physiol. Plant..

[68] Çomaklı E., Bingöl M.S. (2021). Heavy Metal Accumulation of Urban Scots Pine (*Pinus Sylvestris* L.) Plantation. Environ. Monit. Assess..

[69] Juranović Cindrić I., Zeiner M., Starčević A., Stingeder G. (2019). Metals in Pine Needles: Characterisation of Bio-Indicators Depending on Species. Int. J. Environ. Sci. Technol..

[70] Beckett P., Spiers G. Sudbury, Canada—40 Years of a Community Regreening on a Smelter-Impacted Landscape Before After. https://www.atlanticclra.ca/wp-content/uploads/2018/11/Beckett-Presentation.pdf.

[71] Moarefi N., Nkongolo K.K. (2022). Contrasting Tolerance and Gene Expression between White Pine (*Pinus strobus*) and Jack Pine (*P. banksiana*) Exposed to an Increasing Nickel Concentration. Ecol. Genet. Genomics.

[72] Mehes-Smith M., Nkongolo K., Cholewa E., Mehes-Smith M., Nkongolo K., Cholewa E. (2013). Coping Mechanisms of Plants to Metal Contaminated Soil. Environmental Change and Sustainability.

[73] Narendrula-Kotha R., Theriault G., Mehes-Smith M., Kalubi K., Nkongolo K., De Voogt P. (2019). Metal Toxicity and Resistance in Plants and Microorganisms in Terrestrial Ecosystems. Reviews of Environmental Contamination and Toxicology Volume 249.

[74] Park W., Ahn S.-J. (2017). HMA3 Is a Key Factor for Differences in Cd- and Zn-Related Phenotype between Arabidopsis Ws and Col-0 Ecotypes. Plant Biotechnol. Rep..

[75] Song X.-Q., Liu L.-F., Jiang Y.-J., Zhang B.-C., Gao Y.-P., Liu X.-L., Lin Q.-S., Ling H.-Q., Zhou Y.-H. (2013). Disruption of Secondary Wall Cellulose Biosynthesis Alters Cadmium Translocation and Tolerance in Rice Plants. Mol. Plant.

[76] Wu Y., Li X., Chen D., Han X., Li B., Yang Y., Yang Y. (2019). Comparative Expression Analysis of Heavy Metal ATPase Subfamily Genes between Cd-Tolerant and Cd-Sensitive Turnip Landraces. Plant Divers..

[77] Theriault G., Michael P., Nkongolo K. (2016). Comprehensive Transcriptome Analysis of Response to Nickel Stress in White Birch (*Betula papyrifera*). PLoS ONE.

[78] García de la Torre V.S., Coba de la Peña T., Pueyo J.J., Lucas M.M. (2021). Cadmium-Tolerant and -Sensitive Cultivars Identified by Screening of *Medicago Truncatula* Germplasm Display Contrasting Responses to Cadmium Stress. Front. Plant Sci..

[79] Repka V., Fiala R., Čiamporová M., Pavlovkin J. (2016). Effects of ZnCl2 on ROS Generation, Plasma Membrane Properties, and Changes in Protein Expression in Grapevine Root Explants. Biologia.

[80] Takács Z., Tuba Z., Smirnoff N. (2001). Exaggeration of Desiccation Stress by Heavy Metal Pollution in *Tortula Ruralis*: A Pilot Study. Plant Growth Regul..

[81] Gantayat S., Mania S., Pradhan C., Das A.B. (2017). Ionic Stress Induced Cytotoxic Effect of Cadmium and Nickel Ions on Roots of *Allium Cepa* L.. Cytologia.

[82] Hung W.-C., Huang D.-D., Chien P.-S., Yeh C.-M., Chen P.-Y., Chi W.-C., Huang H.-J. (2007). Protein Tyrosine Dephosphorylation during Copper-Induced Cell Death in Rice Roots. Chemosphere.

[83] Domash V.I., Sharpio T.P., Zabreiko S.A., Sosnovskaya T.F. (2008). Proteolytic Enzymes and Trypsin Inhibitors of Higher Plants under Stress Conditions. Russ. J. Bioorganic Chem..

[84] (2021). The Gene Ontology Consortium The Gene Ontology Resource: Enriching a GOld Mine. Nucleic Acids Res..

[85] Faè M., Balestrazzi A., Confalonieri M., Donà M., Macovei A., Valassi A., Giraffa G., Carbonera D. (2014). Copper-Mediated Genotoxic Stress Is Attenuated by the Overexpression of the DNA Repair Gene MtTdp2α (Tyrosyl-DNA Phosphodiesterase 2) in *Medicago truncatula* Plants. Plant Cell Rep..

[86] Macovei A., Balestrazzi A., Confalonieri M., Carbonera D. (2010). The Tyrosyl-DNA Phosphodiesterase Gene Family in *Medicago Truncatula* Gaertn.: Bioinformatic Investigation and Expression Profiles in Response to Copper- and PEG-Mediated Stress. Planta.

[87] El-Beltagi H.S., Sofy M.R., Aldaej M.I., Mohamed H.I. (2020). Silicon Alleviates Copper Toxicity in Flax Plants by Up-Regulating Antioxidant Defense and Secondary Metabolites and Decreasing Oxidative Damage. Sustainability.

[88] Li Z., Chen X., Li S., Wang Z. (2015). Effect of Nickel Chloride on Arabidopsis Genomic DNA and Methylation of 18S rDNA. Electron. J. Biotechnol..

[89] Manna I., Bandyopadhyay M. (2022). Differential Expression of Nickel Toxicity on *Allium Cepa* L. Seeds and Seedlings. Int. J. Environ. Sci. Technol..

[90] Wang J., Moeen-ud-din M., Yin R., Yang S. (2023). ROS Homeostasis Involved in Dose-Dependent Responses of Arabidopsis Seedlings to Copper Toxicity. Genes.

[91] Kim N.-S., Im M.-J., Nkongolo K. (2016). Determination of DNA Methylation Associated with *Acer Rubrum* (Red Maple) Adaptation to Metals: Analysis of Global DNA Modifications and Methylation-Sensitive Amplified Polymorphism. Ecol. Evol..

[92] Shi D., Zhuang K., Xia Y., Zhu C., Chen C., Hu Z., Shen Z. (2017). *Hydrilla Verticillata* Employs Two Different Ways to Affect DNA Methylation under Excess Copper Stress. Aquat. Toxicol..

[93] Zhou J.J., Ren J., Wang X.H., Liu T.K., Hou X.L., Li Y. (2017). Ascorbic Acid Alleviates Toxicity Induced by Excess Copper in *Brassica Campestris* Ssp. Chinensis Makino. Commun. Soil Sci. Plant Anal..

[94] Ince A.G., Karaca M. (2021). Tissue and/or Developmental Stage Specific Methylation of nrDNA in *Capsicum Annuum*. J. Plant Res..

[95] Pätsikkä E., Kairavuo M., Šeršen F., Aro E.-M., Tyystjärvi E. (2002). Excess Copper Predisposes Photosystem II to Photoinhibition in Vivo by Outcompeting Iron and Causing Decrease in Leaf Chlorophyll. Plant Physiol..

[96] Pető A., Lehotai N., Lozano-Juste J., León J., Tari I., Erdei L., Kolbert Z. (2011). Involvement of Nitric Oxide and Auxin in Signal Transduction of Copper-Induced Morphological Responses in Arabidopsis Seedlings. Ann. Bot..

[97] El-Sheekh M.M. (1993). Inhibition of Photosystem II in the Green Alga Scenedesmus Obliquus by Nickel. Biochem. Physiol. Pflanz..

[98] Lešková A., Zvarík M., Araya T., Giehl R.F.H. (2020). Nickel Toxicity Targets Cell Wall-Related Processes and PIN2-Mediated Auxin Transport to Inhibit Root Elongation and Gravitropic Responses in Arabidopsis. Plant Cell Physiol..

[99] Moy A., Czajka K., Michael P., Nkongolo K. (2021). Gene Expression Profiling of Jack Pine (*Pinus banksiana*) under Copper Stress: Identification of Genes Associated with Copper Resistance. PLoS ONE.

[100] Crouzet J., Roland J., Peeters E., Trombik T., Ducos E., Nader J., Boutry M. (2013). NtPDR1, a Plasma Membrane ABC Transporter from Nicotiana Tabacum, Is Involved in Diterpene Transport. Plant Mol. Biol..

[101] Gupta B.B., Selter L.L., Baranwal V.K., Arora D., Mishra S.K., Sirohi P., Poonia A.K., Chaudhary R., Kumar R., Krattinger S.G. (2019). Updated Inventory, Evolutionary and Expression Analyses of G (PDR) Type ABC Transporter Genes of Rice. Plant Physiol. Biochem..

[102] Fernandez L.R., Vandenbussche G., Roosens N., Govaerts C., Goormaghtigh E., Verbruggen N. (2012). Metal Binding Properties and Structure of a Type III Metallothionein from the Metal Hyperaccumulator Plant *Noccaea Caerulescens*. Biochim. Biophys. Acta BBA—Proteins Proteomics.

[103] Ogasawara F., Kodan A., Ueda K. (2020). ABC Proteins in Evolution. FEBS Lett..

[104] Moody J.E., Millen L., Binns D., Hunt J.F., Thomas P.J. (2002). Cooperative, ATP-Dependent Association of the Nucleotide Binding Cassettes during the Catalytic Cycle of ATP-Binding Cassette Transporters *. J. Biol. Chem..

[105] Kim D.-Y., Bovet L., Maeshima M., Martinoia E., Lee Y. (2007). The ABC Transporter AtPDR8 Is a Cadmium Extrusion Pump Conferring Heavy Metal Resistance. Plant J..

[106] Lee M., Lee K., Lee J., Noh E.W., Lee Y. (2005). AtPDR12 Contributes to Lead Resistance in Arabidopsis. Plant Physiol..

[107] Moons A. (2003). Ospdr9, Which Encodes a PDR-Type ABC Transporter, Is Induced by Heavy Metals, Hypoxic Stress and Redox Perturbations in Rice Roots 1. FEBS Lett..

[108] Zhang H., Jing W., Zheng J., Jin Y., Wu D., Cao C., Dong Y., Shi X., Zhang W. (2020). The ATP-Binding Cassette Transporter OsPDR1 Regulates Plant Growth and Pathogen Resistance by Affecting Jasmonates Biosynthesis in Rice. Plant Sci..

[109] Schaaf G., Honsbein A., Meda A.R., Kirchner S., Wipf D., von Wirén N. (2006). AtIREG2 Encodes a Tonoplast Transport Protein Involved in Iron-Dependent Nickel Detoxification in *Arabidopsis Thaliana* Roots. J. Biol. Chem..

[110] Sheng H., Jiang Y., Rahmati M., Chia J.-C., Dokuchayeva T., Kavulych Y., Zavodna T.-O., Mendoza P.N., Huang R., Smieshka L.M. (2021). YSL3-Mediated Copper Distribution Is Required for Fertility, Seed Size and Protein Accumulation in *Brachypodium*. Plant Physiol..

[111] Chowdhury R., Nallusamy S., Shanmugam V., Loganathan A., Muthurajan R., Sivathapandian S.K., Paramasivam J., Duraialagaraja S. (2022). Genome-Wide Understanding of Evolutionary and Functional Relationships of Rice Yellow Stripe-Like (YSL) Transporter Family in Comparison with Other Plant Species. Biologia.

[112] Inoue H., Kobayashi T., Nozoye T., Takahashi M., Kakei Y., Suzuki K., Nakazono M., Nakanishi H., Mori S., Nishizawa N.K. (2009). Rice OsYSL15 Is an Iron-Regulated Iron(III)-Deoxymugineic Acid Transporter Expressed in the Roots and Is Essential for Iron Uptake in Early Growth of the Seedlings *. J. Biol. Chem..

[113] Roberts L.A., Pierson A.J., Panaviene Z., Walker E.L. (2004). Yellow Stripe1. Expanded Roles for the Maize Iron-Phytosiderophore Transporter. Plant Physiol..

[114] Feng S., Tan J., Zhang Y., Liang S., Xiang S., Wang H., Chai T. (2017). Isolation and Characterization of a Novel Cadmium-Regulated Yellow Stripe-Like Transporter (SnYSL3) in *Solanum Nigrum*. Plant Cell Rep..

[115] Zheng L., Yamaji N., Yokosho K., Ma J.F. (2012). YSL16 Is a Phloem-Localized Transporter of the Copper-Nicotianamine Complex That Is Responsible for Copper Distribution in Rice. Plant Cell.

[116] Sasaki I., Nagayama H. (1997). Induction of β-Glucosidase in Botrytis Cinerea by Cell Wall Fractions of the Host Plant. Biosci. Biotechnol. Biochem..

[117] Wang J.-W., Li Y., Zhang Y.-X., Chai T.-Y. (2013). Molecular Cloning and Characterization of a Brassica Juncea Yellow Stripe-like Gene, BjYSL7, Whose Overexpression Increases Heavy Metal Tolerance of Tobacco. Plant Cell Rep..

[118] DiDonato Jr R.J., Roberts L.A., Sanderson T., Eisley R.B., Walker E.L. (2004). Arabidopsis Yellow Stripe-Like2 (YSL2): A Metal-Regulated Gene Encoding a Plasma Membrane Transporter of Nicotianamine–Metal Complexes. Plant J..

[119] Dai J., Wang N., Xiong H., Qiu W., Nakanishi H., Kobayashi T., Nishizawa N.K., Zuo Y. (2018). The Yellow Stripe-Like (YSL) Gene Functions in Internal Copper Transport in Peanut. Genes.

[120] Matijevic L., Romic D., Romic M. (2014). Soil Organic Matter and Salinity Affect Copper Bioavailability in Root Zone and Uptake by *Vicia faba* L. Plants. Environ. Geochem. Health.

[121] Czajka K.M., Michael P., Nkongolo K. (2019). Differential Effects of Nickel Dosages on in Vitro and in Vivo Seed Germination and Expression of a High Affinity Nickel-Transport Family Protein (AT2G16800) in Trembling Aspen (*Populus tremuloides*). Ecotoxicology.

[122] de Abreu-Neto J.B., Turchetto-Zolet A.C., de Oliveira L.F.V., Bodanese Zanettini M.H., Margis-Pinheiro M. (2013). Heavy Metal-Associated Isoprenylated Plant Protein (HIPP): Characterization of a Family of Proteins Exclusive to Plants. FEBS J..

[123] Crowell D.N. (2000). Functional Implications of Protein Isoprenylation in Plants. Prog. Lipid Res..

[124] Tehseen M., Cairns N., Sherson S., Cobbett C.S. (2010). Metallochaperone-like Genes in *Arabidopsis thaliana*. Metallomics.

[125] Barth O., Vogt S., Uhlemann R., Zschiesche W., Humbeck K. (2009). Stress Induced and Nuclear Localized HIPP26 from Arabidopsis Thaliana Interacts via Its Heavy Metal Associated Domain with the Drought Stress Related Zinc Finger Transcription Factor ATHB29. Plant Mol. Biol..

[126] Gao W., Xiao S., Li H.-Y., Tsao S.-W., Chye M.-L. (2009). Arabidopsis Thaliana Acyl-CoA-Binding Protein ACBP2 Interacts with Heavy-Metal-Binding Farnesylated Protein AtFP6. New Phytol..

[127] Dykema P.E., Sipes P.R., Marie A., Biermann B.J., Crowell D.N., Randall S.K. (1999). A New Class of Proteins Capable of Binding Transition Metals. Plant Mol. Biol..

[128] khan I.u., Rono J.K., Zhang B.Q., Liu X.S., Wang M.Q., Wang L.L., Wu X.C., Chen X., Cao H.W., Yang Z.M. (2019). Identification of Novel Rice (*Oryza sativa*) HPP and HIPP Genes Tolerant to Heavy Metal Toxicity. Ecotoxicol. Environ. Saf..

[129] Suzuki N., Yamaguchi Y., Koizumi N., Sano H. (2002). Functional Characterization of a Heavy Metal Binding Protein CdI19 from Arabidopsis. Plant J..

[130] Wei Y., Peng X., Wang X., Wang C. (2023). The Heavy Metal-Associated Isoprenylated Plant Protein (HIPP) Gene Family Plays a Crucial Role in Cadmium Resistance and Accumulation in the Tea Plant (*Camellia sinensis* L.). Ecotoxicol. Environ. Saf..

[131] Shi Y., Jiang N., Wang M., Du Z., Chen J., Huang Y., Li M., Jin Y., Li J., Wan J. (2023). OsHIPP17 Is Involved in Regulating the Tolerance of Rice to Copper Stress. Front. Plant Sci..

[132] Zhang X., Feng H., Feng C., Xu H., Huang X., Wang Q., Duan X., Wang X., Wei G., Huang L. (2015). Isolation and Characterisation of cDNA Encoding a Wheat Heavy Metal-Associated Isoprenylated Protein Involved in Stress Responses. Plant Biol..

[133] Buning C., Comba P. (2000). Protonation of the Copper(I) Form of the Blue Copper Proteins Plastocyanin and Amicyanin—A Molecular Dynamics Study. Eur. J. Inorg. Chem..

[134] De Rienzo F., Gabdoulline R.R., Menziani M.C., Wade R.C. (2000). Blue Copper Proteins: A Comparative Analysis of Their Molecular Interaction Properties. Protein Sci..

[135] Bernal M., Ramiro M.V., Cases R., Picorel R., Yruela I. (2006). Excess Copper Effect on Growth, Chloroplast Ultrastructure, Oxygen-Evolution Activity and Chlorophyll Fluorescence in Glycine Max Cell Suspensions. Physiol. Plant..

[136] Nersissian A.M., Valentine J.S., Mehrabian Z.B., Nalbandyan R.M., Hart P.J., Fraczkiewicz G., Czernuszewicz R.S., Bender C.J., Peisach J., Herrmann R.G. (1996). Cloning, Expression, and Spectroscopic Characterization of Cucumis Sativus Stellacyanin in Its Nonglycosylated Form. Protein Sci..

[137] Nersissian A.M., Valentine J.S., Immoos C., Hill M.G., Hart P.J., Williams G., Herrmann R.G. (1998). Uclacyanins, Stellacyanins, and Plantacyanins Are Distinct Subfamilies of Phytocyanins: Plant-Specific Mononuclear Blue Copper Proteins. Protein Sci..

[138] Chang S., Puryear J.D., Dias M.A.D.L., Funkhouser E.A., Newton R.J., Cairney J. (1996). Gene Expression under Water Deficit in Loblolly Pine (*Pinus taeda*): Isolation and Characterization of cDNA Clones. Physiol. Plant..

[139] Drew J.E., Gatehouse J.A. (1994). Isolation and Characterization of a Pea Pod cDNA Encoding a Putative Blue Copper Protein Correlated with Lignin Deposition. J. Exp. Bot..

[140] Zhu W., Gao E., Shaban M., Wang Y., Wang H., Nie X., Zhu L. (2018). GhUMC1, a Blue Copper-Binding Protein, Regulates Lignin Synthesis and Cotton Immune Response. Biochem. Biophys. Res. Commun..

[141] Li X.-P., Björkman O., Shih C., Grossman A.R., Rosenquist M., Jansson S., Niyogi K.K. (2000). A Pigment-Binding Protein Essential for Regulation of Photosynthetic Light Harvesting. Nature.

[142] Nield J., Funk C., Barber J. (2000). Supermolecular Structure of Photosystem II and Location of the PsbS Protein. Philos. Trans. R. Soc. Lond. B. Biol. Sci..

[143] Nicol L., Nawrocki W.J., Croce R. (2019). Disentangling the Sites of Non-Photochemical Quenching in Vascular Plants. Nat. Plants.

[144] Gruszecki W.I., Grudzinski W., Matula M., Kernen P., Krupa Z. (1999). Light-Induced Excitation Quenching and Structural Transition in Light-Harvesting Complex II. Photosynth. Res..

[145] Gilmore A.M., Hazlett T.L., Debrunner P.G. (1996). Govindjee Photosystem II Chlorophyll a Fluorescence Lifetimes and Intensity Are Independent of the Antenna Size Differences between Barley Wild-Type and Chlorina Mutants: Photochemical Quenching and Xanthophyll Cycle-Dependent Nonphotochemical Quenching of Fluorescence. Photosynth. Res..

[146] Dreuw A., Fleming G.R., Head-Gordon M. (2005). Role of Electron-Transfer Quenching of Chlorophyll Fluorescence by Carotenoids in Non-Photochemical Quenching of Green Plants. Biochem. Soc. Trans..

[147] Zhao X., Chen T., Feng B., Zhang C., Peng S., Zhang X., Fu G., Tao L. (2017). Non-Photochemical Quenching Plays a Key Role in Light Acclimation of Rice Plants Differing in Leaf Color. Front. Plant Sci..

[148] Eudes A., Pereira J.H., Yogiswara S., Wang G., Teixeira Benites V., Baidoo E.E.K., Lee T.S., Adams P.D., Keasling J.D., Loqué D. (2016). Exploiting the Substrate Promiscuity of Hydroxycinnamoyl-CoA:Shikimate Hydroxycinnamoyl Transferase to Reduce Lignin. Plant Cell Physiol..

[149] Cardenas C.L., Costa M.A., Laskar D.D., Moinuddin S.G.A., Lee C., Davin L.B., Lewis N.G. (2021). RNAi Modulation of Chlorogenic Acid and Lignin Deposition in Nicotiana Tabacum and Insufficient Compensatory Metabolic Cross-Talk. J. Nat. Prod..

[150] Kriegshauser L., Knosp S., Grienenberger E., Tatsumi K., Gütle D.D., Sørensen I., Herrgott L., Zumsteg J., Rose J.K.C., Reski R. (2021). Function of the HYDROXYCINNAMOYL-CoA:SHIKIMATE HYDROXYCINNAMOYL TRANSFERASE Is Evolutionarily Conserved in Embryophytes. Plant Cell.

[151] Dong D., Yang Z., Ma Y., Li S., Wang M., Li Y., Liu Z., Jia C., Han L., Chao Y. (2022). Expression of a Hydroxycinnamoyl-CoA Shikimate/Quinate Hydroxycinnamoyl Transferase 4 Gene from Zoysia Japonica (ZjHCT4) Causes Excessive Elongation and Lignin Composition Changes in Agrostis Stolonifera. Int. J. Mol. Sci..

[152] Chao N., Qi Q., Li S., Ruan B., Jiang X., Gai Y. (2021). Characterization and Functional Analysis of the Hydroxycinnamoyl-CoA: Shikimate Hydroxycinnamoyl Transferase (HCT) Gene Family in Poplar. PeerJ.

[153] Varbanova M., Porter K., Lu F., Ralph J., Hammerschmidt R., Jones A.D., Day B. (2011). Molecular and Biochemical Basis for Stress-Induced Accumulation of Free and Bound p-Coumaraldehyde in Cucumber. Plant Physiol..

[154] Hoffmann L., Besseau S., Geoffroy P., Ritzenthaler C., Meyer D., Lapierre C., Pollet B., Legrand M. (2004). Silencing of Hydroxycinnamoyl-Coenzyme A Shikimate/Quinate Hydroxycinnamoyltransferase Affects Phenylpropanoid Biosynthesis[W]. Plant Cell.

[155] Man Ha C., Fine D., Bhatia A., Rao X., Martin M.Z., Engle N.L., Wherritt D.J., Tschaplinski T.J., Sumner L.W., Dixon R.A. (2019). Ectopic Defense Gene Expression Is Associated with Growth Defects in Medicago Truncatula Lignin Pathway Mutants1 [OPEN]. Plant Physiol..

[156] Wagner A., Ralph J., Akiyama T., Flint H., Phillips L., Torr K., Nanayakkara B., Te Kiri L. (2007). Exploring Lignification in Conifers by Silencing Hydroxycinnamoyl-CoA:Shikimate Hydroxycinnamoyltransferase in Pinus Radiata. Proc. Natl. Acad. Sci. USA.

[157] Su M., Liu Y., Lyu J., Zhao S., Wang Y. (2022). Chemical and Structural Responses to Downregulated P-Hydroxycinnamoyl-Coenzyme A: Quinate/Shikimate p-Hydroxycinnamoyltransferase in Poplar Cell Walls. Front. Plant Sci..

[158] Zhou X., Yang S., Lu M., Zhao S., Cai L., Zhang Y., Zhao R., Lv J. (2020). Structure and Monomer Ratio of Lignin in C3H and HCT RNAi Transgenic Poplar Saplings. ChemistrySelect.

[159] Zhao D., Xu C., Luan Y., Shi W., Tang Y., Tao J. (2021). Silicon Enhances Stem Strength by Promoting Lignin Accumulation in Herbaceous Peony (*Paeonia lactiflora* Pall.). Int. J. Biol. Macromol..

[160] Marjamaa K., Kukkola E., Lundell T., Karhunen P., Saranpää P., Fagerstedt K.V. (2006). Monolignol Oxidation by Xylem Peroxidase Isoforms of Norway Spruce (*Picea abies*) and Silver Birch (*Betula pendula*). Tree Physiol..

[161] Kang X., Kirui A., Dickwella Widanage M.C., Mentink-Vigier F., Cosgrove D.J., Wang T. (2019). Lignin-Polysaccharide Interactions in Plant Secondary Cell Walls Revealed by Solid-State NMR. Nat. Commun..

[162] Feijao C., Morreel K., Anders N., Tryfona T., Busse-Wicher M., Kotake T., Boerjan W., Dupree P. (2022). Hydroxycinnamic Acid-Modified Xylan Side Chains and Their Cross-Linking Products in Rice Cell Walls Are Reduced in the Xylosyl Arabinosyl Substitution of Xylan 1 Mutant. Plant J..

[163] Sant’Anna C., Costa L.T., Abud Y., Biancatto L., Miguens F.C., de Souza W. (2013). Sugarcane Cell Wall Structure and Lignin Distribution Investigated by Confocal and Electron Microscopy. Microsc. Res. Tech..

[164] Bezrukova M.V., Fatkhutdinova R.A., Lubyanova A.R., Murzabaev A.R., Fedyaev V.V., Shakirova F.M. (2011). Lectin Involvement in the Development of Wheat Tolerance to Cadmium Toxicity. Russ. J. Plant Physiol..

[165] Ren C., Qi Y., Huang G., Yao S., You J., Hu H. (2020). Contributions of Root Cell Wall Polysaccharides to Cu Sequestration in Castor (*Ricinus communis* L.) Exposed to Different Cu Stresses. J. Environ. Sci..

[166] Yang J., Bak G., Burgin T., Barnes W.J., Mayes H.B., Peña M.J., Urbanowicz B.R., Nielsen E. (2020). Biochemical and Genetic Analysis Identify CSLD3 as a Beta-1,4-Glucan Synthase That Functions during Plant Cell Wall Synthesis[OPEN]. Plant Cell.

[167] Davis J., Brandizzi F., Liepman A.H., Keegstra K. (2010). Arabidopsis Mannan Synthase CSLA9 and Glucan Synthase CSLC4 Have Opposite Orientations in the Golgi Membrane. Plant J..

[168] Suzuki S., Li L., Sun Y.-H., Chiang V.L. (2006). The Cellulose Synthase Gene Superfamily and Biochemical Functions of Xylem-Specific Cellulose Synthase-Like Genes in *Populus trichocarpa*. Plant Physiol..

[169] Little A., Lahnstein J., Jeffery D.W., Khor S.F., Schwerdt J.G., Shirley N.J., Hooi M., Xing X., Burton R.A., Bulone V. (2019). A Novel (1,4)-β-Linked Glucoxylan Is Synthesized by Members of the Cellulose Synthase-Like F Gene Family in Land Plants. ACS Cent. Sci..

[170] Cocuron J.-C., Lerouxel O., Drakakaki G., Alonso A.P., Liepman A.H., Keegstra K., Raikhel N., Wilkerson C.G. (2007). A Gene from the Cellulose Synthase-like C Family Encodes a β-1,4 Glucan Synthase. Proc. Natl. Acad. Sci. USA.

[171] Li M., Xiong G., Li R., Cui J., Tang D., Zhang B., Pauly M., Cheng Z., Zhou Y. (2009). Rice Cellulose Synthase-like D4 Is Essential for Normal Cell-Wall Biosynthesis and Plant Growth. Plant J..

[172] Zeng Y., Himmel M.E., Ding S.-Y. (2017). Visualizing Chemical Functionality in Plant Cell Walls. Biotechnol. Biofuels.

[173] Salmén L., Bergström E. (2009). Cellulose Structural Arrangement in Relation to Spectral Changes in Tensile Loading FTIR. Cellulose.

[174] McCann M.C., Roberts K. (1994). Changes in Cell Wall Architecture during Cell Elongation. J. Exp. Bot..

[175] Zhang Y., Yu J., Wang X., Durachko D.M., Zhang S., Cosgrove D.J. (2021). Molecular Insights into the Complex Mechanics of Plant Epidermal Cell Walls. Science.

[176] Taylor-Teeples M., Lin L., de Lucas M., Turco G., Toal T.W., Gaudinier A., Young N.F., Trabucco G.M., Veling M.T., Lamothe R. (2015). An Arabidopsis Gene Regulatory Network for Secondary Cell Wall Synthesis. Nature.

[177] Zhang N., Li S., Xiong L., Hong Y., Chen Y. (2015). Cellulose-Hemicellulose Interaction in Wood Secondary Cell-Wall. Model. Simul. Mater. Sci. Eng..

[178] Xiao Y., Wu X., Liu D., Yao J., Liang G., Song H., Ismail A.M., Luo J.-S., Zhang Z. (2020). Cell Wall Polysaccharide-Mediated Cadmium Tolerance Between Two Arabidopsis Thaliana Ecotypes. Front. Plant Sci..

[179] Hunter C.T., Kirienko D.H., Sylvester A.W., Peter G.F., McCarty D.R., Koch K.E. (2012). Cellulose Synthase-Like D1 Is Integral to Normal Cell Division, Expansion, and Leaf Development in Maize. Plant Physiol..

[180] Hu H., Zhang R., Tang Y., Peng C., Wu L., Feng S., Chen P., Wang Y., Du X., Peng L. (2019). Cotton CSLD3 Restores Cell Elongation and Cell Wall Integrity Mainly by Enhancing Primary Cellulose Production in the Arabidopsis Cesa6 Mutant. Plant Mol. Biol..

[181] Hu H., Zhang R., Feng S., Wang Y., Wang Y., Fan C., Li Y., Liu Z., Schneider R., Xia T. (2018). Three AtCesA6-like Members Enhance Biomass Production by Distinctively Promoting Cell Growth in Arabidopsis. Plant Biotechnol. J..

[182] Lukowitz W., Nickle T.C., Meinke D.W., Last R.L., Conklin P.L., Somerville C.R. (2001). *Arabidopsis Cyt1* Mutants Are Deficient in a Mannose-1-Phosphate Guanylyltransferase and Point to a Requirement of N-Linked Glycosylation for Cellulose Biosynthesis. Proc. Natl. Acad. Sci. USA.

[183] Strasser R., Seifert G., Doblin M.S., Johnson K.L., Ruprecht C., Pfrengle F., Bacic A., Estevez J.M. (2021). Cracking the “Sugar Code”: A Snapshot of N- and O-Glycosylation Pathways and Functions in Plants Cells. Front. Plant Sci..

[184] Łuczak M., Bugajewska A., Wojtaszek P. (2008). Inhibitors of Protein Glycosylation or Secretion Change the Pattern of Extracellular Proteins in Suspension-Cultured Cells of Arabidopsis Thaliana. Plant Physiol. Biochem..

[185] Lerouxel O., Mouille G., Andème-Onzighi C., Bruyant M.-P., Séveno M., Loutelier-Bourhis C., Driouich A., Höfte H., Lerouge P. (2005). Mutants in DEFECTIVE GLYCOSYLATION, an Arabidopsis Homolog of an Oligosaccharyltransferase Complex Subunit, Show Protein Underglycosylation and Defects in Cell Differentiation and Growth. Plant J..

[186] Lige B., Ma S., van Huystee R.B. (2001). The Effects of the Site-Directed Removal of N-Glycosylation from Cationic Peanut Peroxidase on Its Function. Arch. Biochem. Biophys..

[187] Pauly M., Eberhard S., Albersheim P., Darvill A., York W.S. (2001). Effects of the Mur1 Mutation on Xyloglucans Produced by Suspension-Cultured Arabidopsis Thaliana Cells. Planta.

[188] Lamanchai K., Salmon D.L., Smirnoff N., Sutthinon P., Roytrakul S., Leetanasaksakul K., Kittisenachai S., Jantasuriyarat C. (2022). OsVTC1-1 RNAi Mutant with Reduction of Ascorbic Acid Synthesis Alters Cell Wall Sugar Composition and Cell Wall-Associated Proteins. Agronomy.

[189] Voxeur A., Soubigou-Taconnat L., Legée F., Sakai K., Antelme S., Durand-Tardif M., Lapierre C., Sibout R. (2017). Altered Lignification in Mur1-1 a Mutant Deficient in GDP-L-Fucose Synthesis with Reduced RG-II Cross Linking. PLoS ONE.

[190] Rocha J., Cicéron F., Lerouxel O., Breton C., de Sanctis D. (2016). The Galactoside 2-α-l-Fucosyltransferase FUT1 from Arabidopsis Thaliana: Crystallization and Experimental MAD Phasing. Acta Crystallogr. Sect. F Struct. Biol. Commun..

[191] Ko J.-H., Yang S.H., Han K.-H. (2006). Upregulation of an Arabidopsis RING-H2 Gene, XERICO, Confers Drought Tolerance through Increased Abscisic Acid Biosynthesis. Plant J..

[192] Zeng D.-E., Hou P., Xiao F., Liu Y. (2014). Overexpressing a Novel RING-H2 Finger Protein Gene, OsRHP1, Enhances Drought and Salt Tolerance in Rice (*Oryza sativa* L.). J. Plant Biol..

[193] Atkinson R.G., Sutherland P.W., Johnston S.L., Gunaseelan K., Hallett I.C., Mitra D., Brummell D.A., Schröder R., Johnston J.W., Schaffer R.J. (2012). Down-Regulation of *POLYGALACTURONASE1* Alters Firmness, Tensile Strength and Water Loss in Apple (*Malus* x *domestica*) Fruit. BMC Plant Biol..

[194] Rowell R.M., Pettersen R., Tshabalala M.A. (2012). Cell Wall Chemistry.

[195] Cao C., Yang Z., Han L., Jiang X., Ji G. (2015). Study on in Situ Analysis of Cellulose, Hemicelluloses and Lignin Distribution Linked to Tissue Structure of Crop Stalk Internodal Transverse Section Based on FTIR Microspectroscopic Imaging. Cellulose.

[196] Orfila C., Huisman M.M., Willats W.G., van Alebeek G.-J.W., Schols H.A., Seymour G.B., Knox P.J. (2002). Altered Cell Wall Disassembly during Ripening of Cnr Tomato Fruit: Implications for Cell Adhesion and Fruit Softening. Planta.

[197] Mohapatra S., Halder S., Chaudhari S.R., Netz R.R., Mogurampelly S. (2023). Insights into the Structure and Ion Transport of Pectin-[BMIM][PF6] Electrolytes. J. Chem. Phys..

[198] Rondeau-Mouro C., Defer D., Leboeuf E., Lahaye M. (2008). Assessment of Cell Wall Porosity in *Arabidopsis Thaliana* by NMR Spectroscopy. Int. J. Biol. Macromol..

[199] Hwang J., Pyun Y.R., Kokini J.L. (1993). Sidechains of Pectins: Some Thoughts on Their Role in Plant Cell Walls and Foods. Food Hydrocoll..

[200] Forand A.D., Finfrock Y.Z., Lavier M., Stobbs J., Qin L., Wang S., Karunakaran C., Wei Y., Ghosh S., Tanino K.K. (2022). With a Little Help from My Cell Wall: Structural Modifications in Pectin May Play a Role to Overcome Both Dehydration Stress and Fungal Pathogens. Plants.

[201] Paynel F., Schaumann A., Arkoun M., Douchiche O., Morvan C. (2009). Temporal Regulation of Cell-Wall Pectin Methylesterase and Peroxidase Isoforms in Cadmium-Treated Flax Hypocotyl. Ann. Bot..

[202] Sharma S., Uttam K.N. (2018). Early Stage Detection of Stress Due to Copper on Maize (*Zea mays* L.) by Laser-Induced Fluorescence and Infrared Spectroscopy. J. Appl. Spectrosc..

[203] Liu H., Ma Y., Chen N., Guo S., Liu H., Guo X., Chong K., Xu Y. (2014). Overexpression of Stress-Inducible OsBURP16, the β Subunit of Polygalacturonase 1, Decreases Pectin Content and Cell Adhesion and Increases Abiotic Stress Sensitivity in Rice. Plant Cell Environ..

[204] Ohara T., Takeuchi H., Sato J., Nakamura A., Ichikawa H., Yokoyama R., Nishitani K., Minami E., Satoh S., Iwai H. (2021). Structural Alteration of Rice Pectin Affects Cell Wall Mechanical Strength and Pathogenicity of the Rice Blast Fungus Under Weak Light Conditions. Plant Cell Physiol..

[205] Khan S., Zafar A., Naseem I. (2018). Copper-Redox Cycling by Coumarin-Di(2-Picolyl)Amine Hybrid Molecule Leads to ROS-Mediated DNA Damage and Apoptosis: A Mechanism for Cancer Chemoprevention. Chem. Biol. Interact..

[206] Sun Y., Ji K., Liang B., Du Y., Jiang L., Wang J., Kai W., Zhang Y., Zhai X., Chen P. (2017). Suppressing ABA Uridine Diphosphate Glucosyltransferase (SlUGT75C1) Alters Fruit Ripening and the Stress Response in Tomato. Plant J..

[207] Chen T.-T., Liu F.-F., Xiao D.-W., Jiang X.-Y., Li P., Zhao S.-M., Hou B., Li Y. (2020). The Arabidopsis UDP-glycosyltransferase75B1, Conjugates Abscisic Acid and Affects Plant Response to Abiotic Stresses. Plant Mol. Biol..

[208] Song J., Shang L., Wang X., Xing Y., Xu W., Zhang Y., Wang T., Li H., Zhang J., Ye Z. (2021). MAPK11 Regulates Seed Germination and ABA Signaling in Tomato by Phosphorylating SnRKs. J. Exp. Bot..

[209] Sun M., Tuan P.A., Izydorczyk M.S., Ayele B.T. (2020). Ethylene Regulates Post-Germination Seedling Growth in Wheat through Spatial and Temporal Modulation of ABA/GA Balance. J. Exp. Bot..

[210] Tao Q., Jupa R., Dong Q., Yang X., Liu Y., Li B., Yuan S., Yin J., Xu Q., Li T. (2021). Abscisic Acid-Mediated Modifications in Water Transport Continuum Are Involved in Cadmium Hyperaccumulation in Sedum Alfredii. Chemosphere.

[211] Hsu Y.T., Kao C.H. (2003). Role of Abscisic Acid in Cadmium Tolerance of Rice (*Oryza sativa* L.) Seedlings. Plant Cell Environ..

[212] Deng B., Zhang W., Yang H. (2022). Abscisic Acid Decreases Cell Death in Malus Hupehensis Rehd. Under Cd Stress by Reducing Root Cd2+ Influx and Leaf Transpiration. J. Plant Growth Regul..

[213] Estrada-Melo A.C., Chao C., Reid M.S., Jiang C.-Z. (2015). Overexpression of an ABA Biosynthesis Gene Using a Stress-Inducible Promoter Enhances Drought Resistance in Petunia. Hortic. Res..

[214] Wang M., Zhang Y., Zhu C., Yao X., Zheng Z., Tian Z., Cai X. (2021). EkFLS Overexpression Promotes Flavonoid Accumulation and Abiotic Stress Tolerance in Plant. Physiol. Plant..

[215] Lee H.Y., Jang G., Um T., Kim J.-K., Lee J.S., Do Choi Y. (2015). The Soluble ABA Receptor PYL8 Regulates Drought Resistance by Controlling ABA Signaling in Arabidopsis. Plant Biotechnol. Rep..

[216] Tung S.A., Smeeton R., White C.A., Black C.R., Taylor I.B., Hilton H.W., Thompson A.J. (2008). Over-Expression of LeNCED1 in Tomato (*Solanum lycopersicum* L.) with the rbcS3C Promoter Allows Recovery of Lines That Accumulate Very High Levels of Abscisic Acid and Exhibit Severe Phenotypes. Plant Cell Environ..

[217] Johannesson H., Wang Y., Hanson J., Engström P. (2003). The Arabidopsis Thaliana Homeobox Gene ATHB5 Is a Potential Regulator of Abscisic Acid Responsiveness in Developing Seedlings. Plant Mol. Biol..

[218] Fan S.K., Fang X.Z., Guan M.Y., Ye Y.Q., Lin X.Y., Du S.T., Jin C.W. (2014). Exogenous Abscisic Acid Application Decreases Cadmium Accumulation in Arabidopsis Plants, Which Is Associated with the Inhibition of IRT1-Mediated Cadmium Uptake. Front. Plant Sci..

[219] Zhao F.J., Jiang R.F., Dunham S.J., McGrath S.P. (2006). Cadmium Uptake, Translocation and Tolerance in the Hyperaccumulator Arabidopsis Halleri. New Phytol..

[220] Wang Z., Li H., Wei Z., Sun H., He Y., Gao J., Yang Z., You J. (2021). Overexpression of UDP-Glycosyltransferase Genes Enhanced Aluminum Tolerance through Disrupting Cell Wall Polysaccharide Components in Soybean. Plant Soil.

[221] Hu H., Qian P., Ye M., Mu K., Wang S., Chen M., Ma H. (2022). GmUGT73F4 Plays Important Roles in Enhancing Seed Vitality and Tolerance to Abiotic Stresses in Transgenic Arabidopsis. Plant Cell Tissue Organ Cult. PCTOC.

[222] Wang T., Li P., Mu T., Dong G., Zheng C., Jin S., Chen T., Hou B., Li Y. (2020). Overexpression of UGT74E2, an Arabidopsis IBA Glycosyltransferase, Enhances Seed Germination and Modulates Stress Tolerance via ABA Signaling in Rice. Int. J. Mol. Sci..

[223] Lu M., Guo J., Dong D., Zhang M., Li Q., Cao Y., Dong Y., Chen C., Jin X. (2023). UDP-Glycosyltransferase Gene SlUGT73C1 from Solanum Lycopersicum Regulates Salt and Drought Tolerance in *Arabidopsis thaliana* L.. Funct. Integr. Genom..

[224] Zhang K., Sun Y., Li M., Long R. (2021). CrUGT87A1, a UDP-Sugar Glycosyltransferases (UGTs) Gene from Carex Rigescens, Increases Salt Tolerance by Accumulating Flavonoids for Antioxidation in *Arabidopsis thaliana*. Plant Physiol. Biochem..

[225] Li P., Li Y., Wang B., Yu H., Li Q., Hou B. (2017). The Arabidopsis UGT87A2, a Stress-Inducible Family 1 Glycosyltransferase, Is Involved in the Plant Adaptation to Abiotic Stresses. Physiol. Plant..

[226] Chung H.S., Howe G.A. (2009). A Critical Role for the TIFY Motif in Repression of Jasmonate Signaling by a Stabilized Splice Variant of the JASMONATE ZIM-Domain Protein JAZ10 in Arabidopsis. Plant Cell.

[227] Hakata M., Muramatsu M., Nakamura H., Hara N., Kishimoto M., Iida-Okada K., Kajikawa M., Imai-Toki N., Toki S., Nagamura Y. (2017). Overexpression of TIFY Genes Promotes Plant Growth in Rice through Jasmonate Signaling. Biosci. Biotechnol. Biochem..

[228] Liu X., Yu F., Yang G., Liu X., Peng S. (2022). Identification of TIFY Gene Family in Walnut and Analysis of Its Expression under Abiotic Stresses. BMC Genom..

[229] Zhang L., You J., Chan Z. (2015). Identification and Characterization of TIFY Family Genes in Brachypodium Distachyon. J. Plant Res..

[230] Liu Y.-L., Zheng L., Jin L.-G., Liu Y.-X., Kong Y.-N., Wang Y.-X., Yu T.-F., Chen J., Zhou Y.-B., Chen M. (2022). Genome-Wide Analysis of the Soybean TIFY Family and Identification of GmTIFY10e and GmTIFY10g Response to Salt Stress. Front. Plant Sci..

[231] Ye H., Du H., Tang N., Li X., Xiong L. (2009). Identification and Expression Profiling Analysis of TIFY Family Genes Involved in Stress and Phytohormone Responses in Rice. Plant Mol. Biol..

[232] Zhang C., Yang R., Zhang T., Zheng D., Li X., Zhang Z.B., Li L.G., Wu Z.Y. (2023). ZmTIFY16, a Novel Maize TIFY Transcription Factor Gene, Promotes Root Growth and Development and Enhances Drought and Salt Tolerance in Arabidopsis and *Zea mays*. Plant Growth Regul..

[233] Zhu D., Li R., Liu X., Sun M., Wu J., Zhang N., Zhu Y. (2014). The Positive Regulatory Roles of the TIFY10 Proteins in Plant Responses to Alkaline Stress. PLoS ONE.

[234] Maksymiec W., Krupa Z. (2006). The Effects of Short-Term Exposition to Cd, Excess Cu Ions and Jasmonate on Oxidative Stress Appearing in Arabidopsis Thaliana. Environ. Exp. Bot..

[235] Brioudes F., Joly C., Szécsi J., Varaud E., Leroux J., Bellvert F., Bertrand C., Bendahmane M. (2009). Jasmonate Controls Late Development Stages of Petal Growth in Arabidopsis Thaliana. Plant J..

[236] Noir S., Bömer M., Takahashi N., Ishida T., Tsui T.-L., Balbi V., Shanahan H., Sugimoto K., Devoto A. (2013). Jasmonate Controls Leaf Growth by Repressing Cell Proliferation and the Onset of Endoreduplication While Maintaining a Potential Stand-By Mode. Plant Physiol..

[237] Attaran E., Major I.T., Cruz J.A., Rosa B.A., Koo A.J.K., Chen J., Kramer D.M., He S.Y., Howe G.A. (2014). Temporal Dynamics of Growth and Photosynthesis Suppression in Response to Jasmonate Signaling. Plant Physiol..

[238] Sirhindi G., Mushtaq R., Gill S.S., Sharma P., Abd_Allah E.F., Ahmad P. (2020). Jasmonic Acid and Methyl Jasmonate Modulate Growth, Photosynthetic Activity and Expression of Photosystem II Subunit Genes in *Brassica oleracea* L.. Sci. Rep..

[239] Keramat B., Kalantari K.M., Arvin M.J. (2010). Effects of Methyl Jasmonate Treatment on Alleviation of Cadmium Damages in Soybean. J. Plant Nutr..

[240] Held M., Baldwin I.T. (2005). Soil Degradation Slows Growth and Inhibits Jasmonate-Induced Resistance in Artemisia Vulgaris. Ecol. Appl..

[241] Abdollahi A., Farsad-Akhtar N., Mohajel Kazemi E., Kolahi M. (2023). Investigation of the Combined Effects of Cadmium Chloride, Silver Nitrate, Lead Nitrate, Methyl Jasmonate, and Salicylic Acid on Morphometric and Biochemical Characteristics of St. John’s Wort. Physiol. Mol. Biol. Plants.

[242] Yan Z., Li X., Chen J., Tam N.F.-Y. (2015). Combined Toxicity of Cadmium and Copper in Avicennia Marina Seedlings and the Regulation of Exogenous Jasmonic Acid. Ecotoxicol. Environ. Saf..

[243] Dai H., Wei S., Pogrzeba M., Rusinowski S., Krzyżak J., Jia G. (2020). Exogenous Jasmonic Acid Decreased Cu Accumulation by Alfalfa and Improved Its Photosynthetic Pigments and Antioxidant System. Ecotoxicol. Environ. Saf..

[244] Barnes J.R., Lorenz W.W., Dean J.F.D. (2008). Characterization of a 1-Aminocyclopropane-1-Carboxylate Synthase Gene from Loblolly Pine (*Pinus taeda* L.). Gene.

[245] Stearns J.C., Shah S., Greenberg B.M., Dixon D.G., Glick B.R. (2005). Tolerance of Transgenic Canola Expressing 1-Aminocyclopropane-1-Carboxylic Acid Deaminase to Growth Inhibition by Nickel. Plant Physiol. Biochem..

[246] Kolbert Z., Oláh D., Molnár Á., Szőllősi R., Erdei L., Ördög A. (2020). Distinct Redox Signalling and Nickel Tolerance in Brassica Juncea and Arabidopsis Thaliana. Ecotoxicol. Environ. Saf..

[247] Kong X., Li C., Zhang F., Yu Q., Gao S., Zhang M., Tian H., Zhang J., Yuan X., Ding Z. (2018). Ethylene Promotes Cadmium-Induced Root Growth Inhibition through EIN3 Controlled XTH33 and LSU1 Expression in Arabidopsis. Plant Cell Environ..

[248] Wakeel A., Gan Y. (2018). A Model for the Ethylene-Mediated Auxin Distribution under Cr(VI) Stress in Arabidopsis Thaliana. Plant Signal. Behav..

[249] Mir I.R., Rather B.A., Sehar Z., Masood A., Khan N.A. (2023). Nitric Oxide in Co-Ordination with Nitrogen Reverses Cadmium-Inhibited Photosynthetic Activity by Interacting with Ethylene Synthesis, Strengthening the Antioxidant System, and Nitrogen and Sulfur Assimilation in Mustard (*Brassica juncea* L.). Sci. Hortic..

[250] Lorenzo O., Piqueras R., Sánchez-Serrano J.J., Solano R. (2003). ETHYLENE RESPONSE FACTOR1 Integrates Signals from Ethylene and Jasmonate Pathways in Plant Defense[W]. Plant Cell.

[251] Lee H.Y., Chen Y.-C., Kieber J.J., Yoon G.M. (2017). Regulation of the Turnover of ACC Synthases by Phytohormones and Heterodimerization in Arabidopsis. Plant J..

[252] Alves L.R., Rodrigues dos Reis A., Prado E.R., Lavres J., Pompeu G.B., Azevedo R.A., Gratão P.L. (2019). New Insights into Cadmium Stressful-Conditions: Role of Ethylene on Selenium-Mediated Antioxidant Enzymes. Ecotoxicol. Environ. Saf..

[253] Singh N., Gaddam S.R., Singh D., Trivedi P.K. (2021). Regulation of Arsenic Stress Response by Ethylene Biosynthesis and Signaling in Arabidopsis Thaliana. Environ. Exp. Bot..

[254] Wang Y., Yuan M., Li Z., Niu Y., Jin Q., Zhu B., Xu Y. (2020). Effects of Ethylene Biosynthesis and Signaling on Oxidative Stress and Antioxidant Defense System in Nelumbo Nucifera G. under Cadmium Exposure. Environ. Sci. Pollut. Res..

[255] Zhang B., Shang S., Jabeen Z., Zhang G. (2014). Involvement of Ethylene in Alleviation of Cd Toxicity by NaCl in Tobacco Plants. Ecotoxicol. Environ. Saf..

[256] Song W.Y., Peng S.P., Shao C.Y., Shao H.B., Yang H.C. (2014). Ethylene Glycol Tetra-Acetic Acid and Salicylic Acid Improve Anti-Oxidative Ability of Maize Seedling Leaves under Heavy-Metal and Polyethylene Glycol 6000-Simulated Drought Stress. Plant Biosyst.—Int. J. Deal. Asp. Plant Biol..

[257] Li Y., Li D., E L., Yang J., Liu W., Xu M., Ye J. (2023). ZmDRR206 Regulates Nutrient Accumulation in Endosperm through Its Role in Cell Wall Biogenesis during Maize Kernel Development. Int. J. Mol. Sci..

[258] Gang D.R., Costa M.A., Fujita M., Dinkova-Kostova A.T., Wang H.-B., Burlat V., Martin W., Sarkanen S., Davin L.B., Lewis N.G. (1999). Regiochemical Control of Monolignol Radical Coupling: A New Paradigm for Lignin and Lignan Biosynthesis. Chem. Biol..

[259] Li L., Sun W., Zhou P., Wei H., Wang P., Li H., Rehman S., Li D., Zhuge Q. (2021). Genome-Wide Characterization of Dirigent Proteins in Populus: Gene Expression Variation and Expression Pattern in Response to Marssonina Brunnea and Phytohormones. Forests.

[260] Xia Y., Liu J., Wang Y., Zhang X., Shen Z., Hu Z. (2018). Ectopic Expression of Vicia Sativa Caffeoyl-CoA O-Methyltransferase (VsCCoAOMT) Increases the Uptake and Tolerance of Cadmium in Arabidopsis. Environ. Exp. Bot..

[261] Gao W., Wang X.J., Yu C.C., Feng W.J., Hua D.L., Kang G.Z., Zhao P. (2021). Comparative Morpho-Physiological Analyses Revealed H2O2-Induced Different Cadmium Accumulation in Two Wheat Cultivars (*Triticum aestivum* L.). Environ. Exp. Bot..

[262] MacFarlane G.R., Burchett M.D. (2000). Cellular Distribution of Copper, Lead and Zinc in the Grey Mangrove, Avicennia Marina (Forsk.) Vierh. Aquat. Bot..

[263] Lewis N.G., Davin L.B. (1994). Evolution of Lignan and Neolignan Biochemical Pathways. Isopentenoids and Other Natural Products.

[264] Wei M., Yu C., Ge B., Liu Y., Zhang H., Duan C., Zhang J., Mao T., Huang H., Xie Y. (2022). The Phenylcoumaran Benzylic Ether Reductase Gene PtPCBER Improves the Salt Tolerance of Transgenic Poplar through Lignan-Mediated Reactive Oxygen Species Scavenging. Environ. Exp. Bot..

[265] Li L., Sun W., Wang P., Li H., Rehman S., Li D., Zhuge Q. (2022). Characterization, Expression, and Functional Analysis of the Pathogenesis-Related Gene PtDIR11 in Transgenic Poplar. Int. J. Biol. Macromol..

[266] Niculaes C., Morreel K., Kim H., Lu F., McKee L.S., Ivens B., Haustraete J., Vanholme B., Rycke R.D., Hertzberg M. (2014). Phenylcoumaran Benzylic Ether Reductase Prevents Accumulation of Compounds Formed under Oxidative Conditions in Poplar Xylem. Plant Cell.

[267] Bollella P., Medici L., Tessema M., Poloznikov A.A., Hushpulian D.M., Tishkov V.I., Andreu R., Leech D., Megersa N., Marcaccio M. (2018). Highly Sensitive, Stable and Selective Hydrogen Peroxide Amperometric Biosensors Based on Peroxidases from Different Sources Wired by Os-Polymer: A Comparative Study. Solid State Ion..

[268] Ji Y., Wu P., Zhang J., Zhang J., Zhou Y., Peng Y., Zhang S., Cai G., Gao G. (2018). Heavy Metal Accumulation, Risk Assessment and Integrated Biomarker Responses of Local Vegetables: A Case Study along the Le’an River. Chemosphere.

[269] Kawano T. (2003). Roles of the Reactive Oxygen Species-Generating Peroxidase Reactions in Plant Defense and Growth Induction. Plant Cell Rep..

[270] Tugbaeva A., Wuriyanghan H., Maleva M. (2022). Copper Stress Enhances the Lignification of Axial Organs in Zinnia Elegans. Horticulturae.

[271] Zhang A., Lu F., Sun R., Ralph J. (2009). Ferulate–Coniferyl Alcohol Cross-Coupled Products Formed by Radical Coupling Reactions. Planta.

[272] Laitinen T., Morreel K., Delhomme N., Gauthier A., Schiffthaler B., Nickolov K., Brader G., Lim K.-J., Teeri T.H., Street N.R. (2017). A Key Role for Apoplastic H2O2 in Norway Spruce Phenolic Metabolism. Plant Physiol..

[273] Kidwai M., Dhar Y.V., Gautam N., Tiwari M., Ahmad I.Z., Asif M.H., Chakrabarty D. (2019). Oryza Sativa Class III Peroxidase (OsPRX38) Overexpression in Arabidopsis Thaliana Reduces Arsenic Accumulation Due to Apoplastic Lignification. J. Hazard. Mater..

[274] Zhang S., Wu J., Yuan D., Zhang D., Huang Z., Xiao L., Yang C. (2014). Perturbation of Auxin Homeostasis Caused by Mitochondrial FtSH4 Gene-Mediated Peroxidase Accumulation Regulates Arabidopsis Architecture. Mol. Plant.

[275] Yuan H.-M., Xu H.-H., Liu W.-C., Lu Y.-T. (2013). Copper Regulates Primary Root Elongation Through PIN1-Mediated Auxin Redistribution. Plant Cell Physiol..

[276] Wang H.-Q., Xuan W., Huang X.-Y., Mao C., Zhao F.-J. (2020). Cadmium Inhibits Lateral Root Emergence in Rice by Disrupting OsPIN-Mediated Auxin Distribution and the Protective Effect of OsHMA3. Plant Cell Physiol..

[277] Sofo A., Khan N.A., D’Ippolito I., Reyes F. (2022). Subtoxic Levels of Some Heavy Metals Cause Differential Root-Shoot Structure, Morphology and Auxins Levels in *Arabidopsis thaliana*. Plant Physiol. Biochem..

[278] Mustafiz A., Singh A.K., Pareek A., Sopory S.K., Singla-Pareek S.L. (2011). Genome-Wide Analysis of Rice and Arabidopsis Identifies Two Glyoxalase Genes That Are Highly Expressed in Abiotic Stresses. Funct. Integr. Genomics.

[279] Mustafiz A., Ghosh A., Tripathi A.K., Kaur C., Ganguly A.K., Bhavesh N.S., Tripathi J.K., Pareek A., Sopory S.K., Singla-Pareek S.L. (2014). A Unique Ni2+ -Dependent and Methylglyoxal-Inducible Rice Glyoxalase I Possesses a Single Active Site and Functions in Abiotic Stress Response. Plant J..

[280] Welchen E., Schmitz J., Fuchs P., García L., Wagner S., Wienstroer J., Schertl P., Braun H.-P., Schwarzländer M., Gonzalez D.H. (2016). D-Lactate Dehydrogenase Links Methylglyoxal Degradation and Electron Transport through Cytochrome c. Plant Physiol..

[281] Wang Y., Ye X.-Y., Qiu X.-M., Li Z.-G. (2019). Methylglyoxal Triggers the Heat Tolerance in Maize Seedlings by Driving AsA-GSH Cycle and Reactive Oxygen Species-/Methylglyoxal-Scavenging System. Plant Physiol. Biochem..

[282] Hoque T.S., Uraji M., Ye W., Hossain M.A., Nakamura Y., Murata Y. (2012). Methylglyoxal-Induced Stomatal Closure Accompanied by Peroxidase-Mediated ROS Production in Arabidopsis. J. Plant Physiol..

[283] Hasanuzzaman M., Alam M.M., Nahar K., Mohsin S.M., Bhuyan M.H.M.B., Parvin K., Hawrylak-Nowak B., Fujita M. (2019). Silicon-Induced Antioxidant Defense and Methylglyoxal Detoxification Works Coordinately in Alleviating Nickel Toxicity in *Oryza sativa* L.. Ecotoxicology.

[284] Mano J., Miyatake F., Hiraoka E., Tamoi M. (2009). Evaluation of the Toxicity of Stress-Related Aldehydes to Photosynthesis in Chloroplasts. Planta.

[285] Hoque T.S., Uraji M., Tuya A., Nakamura Y., Murata Y. (2012). Methylglyoxal Inhibits Seed Germination and Root Elongation and Up-Regulates Transcription of Stress-Responsive Genes in ABA-Dependent Pathway in Arabidopsis. Plant Biol..

[286] Yiğit İ., Atici Ö. (2022). Seed Priming with Nitric Oxide Mitigates Exogenous Methylglyoxal Toxicity by Restoring Glyoxalase and Antioxidant Systems in Germinating Maize (*Zea mays* L.) Seeds. Cereal Res. Commun..

[287] Gambhir P., Raghuvanshi U., Parida A.P., Kujur S., Sharma S., Sopory S.K., Kumar R., Sharma A.K. (2023). Elevated Methylglyoxal Levels Inhibit Tomato Fruit Ripening by Preventing Ethylene Biosynthesis. Plant Physiol..

[288] Chen M., Thelen J.J. (2010). The Plastid Isoform of Triose Phosphate Isomerase Is Required for the Postgerminative Transition from Heterotrophic to Autotrophic Growth in Arabidopsis. Plant Cell.

[289] Mostofa M.G., Hossain M.A., Siddiqui M.N., Fujita M., Tran L.-S.P. (2017). Phenotypical, Physiological and Biochemical Analyses Provide Insight into Selenium-Induced Phytotoxicity in Rice Plants. Chemosphere.

[290] Shumilina J., Kusnetsova A., Tsarev A., Janse van Rensburg H.C., Medvedev S., Demidchik V., Van den Ende W., Frolov A. (2019). Glycation of Plant Proteins: Regulatory Roles and Interplay with Sugar Signalling?. Int. J. Mol. Sci..

[291] Adak S., Agarwal T., Das P., Ray S., Lahiri Majumder A. (2023). Characterization of Myo-Inositol Oxygenase from Rice (OsMIOX): Influence of Salinity Stress in Different Indica Rice Cultivars. Physiol. Mol. Biol. Plants.

[292] Lyczakowski J.J., Wicher K.B., Terrett O.M., Faria-Blanc N., Yu X., Brown D., Krogh K.B.R.M., Dupree P., Busse-Wicher M. (2017). Removal of Glucuronic Acid from Xylan Is a Strategy to Improve the Conversion of Plant Biomass to Sugars for Bioenergy. Biotechnol. Biofuels.

[293] Lyczakowski J.J., Yu L., Terrett O.M., Fleischmann C., Temple H., Thorlby G., Sorieul M., Dupree P. (2021). Two Conifer GUX Clades Are Responsible for Distinct Glucuronic Acid Patterns on Xylan. New Phytol..

[294] Silveira R.L., Stoyanov S.R., Gusarov S., Skaf M.S., Kovalenko A. (2013). Plant Biomass Recalcitrance: Effect of Hemicellulose Composition on Nanoscale Forces That Control Cell Wall Strength. J. Am. Chem. Soc..

[295] Kanter U., Usadel B., Guerineau F., Li Y., Pauly M., Tenhaken R. (2005). The Inositol Oxygenase Gene Family of Arabidopsis Is Involved in the Biosynthesis of Nucleotide Sugar Precursors for Cell-Wall Matrix Polysaccharides. Planta.

[296] Zhu X.F., Wan J.X., Wu Q., Zhao X.S., Zheng S.J., Shen R.F. (2017). PARVUS Affects Aluminium Sensitivity by Modulating the Structure of Glucuronoxylan in *Arabidopsis thaliana*. Plant Cell Environ..

[297] Yang J., Yang J., Zhao L., Gu L., Wu F., Tian W., Sun Y., Zhang S., Su H., Wang L. (2021). Ectopic Expression of a Malus Hupehensis Rehd. Myo- Inositol Oxygenase Gene (MhMIOX2) Enhances Tolerance to Salt Stress. Sci. Hortic..

[298] Duan J., Zhang M., Zhang H., Xiong H., Liu P., Ali J., Li J., Li Z. (2012). OsMIOX, a Myo-Inositol Oxygenase Gene, Improves Drought Tolerance through Scavenging of Reactive Oxygen Species in Rice (*Oryza sativa* L.). Plant Sci..

[299] Toyofuku K., Kasahara M., Yamaguchi J. (2000). Characterization and Expression of Monosaccharide Transporters (OsMSTs) in Rice. Plant Cell Physiol..

[300] Sherson S.M., Alford H.L., Forbes S.M., Wallace G., Smith S.M. (2003). Roles of Cell-wall Invertases and Monosaccharide Transporters in the Growth and Development of Arabidopsis. J. Exp. Bot..

[301] Aslam M., Greaves J.G., Jakada B.H., Fakher B., Wang X., Qin Y. (2022). AcCIPK5, a Pineapple CBL-Interacting Protein Kinase, Confers Salt, Osmotic and Cold Stress Tolerance in Transgenic Arabidopsis. Plant Sci. Int. J. Exp. Plant Biol..

[302] Wingenter K., Schulz A., Wormit A., Wic S., Trentmann O., Hoermiller I.I., Heyer A.G., Marten I., Hedrich R., Neuhaus H.E. (2010). Increased Activity of the Vacuolar Monosaccharide Transporter TMT1 Alters Cellular Sugar Partitioning, Sugar Signaling, and Seed Yield in Arabidopsis. Plant Physiol..

[303] Chigri F., Flosdorff S., Pilz S., Kölle E., Dolze E., Gietl C., Vothknecht U.C. (2012). The Arabidopsis Calmodulin-like Proteins AtCML30 and AtCML3 Are Targeted to Mitochondria and Peroxisomes, Respectively. Plant Mol. Biol..

[304] Scholz S.S., Vadassery J., Heyer M., Reichelt M., Bender K.W., Snedden W.A., Boland W., Mithöfer A. (2014). Mutation of the Arabidopsis Calmodulin-Like Protein CML37 Deregulates the Jasmonate Pathway and Enhances Susceptibility to Herbivory. Mol. Plant.

[305] Fang H., Liu Z., Long Y., Liang Y., Jin Z., Zhang L., Liu D., Li H., Zhai J., Pei Y. (2017). The Ca2+/Calmodulin2-Binding Transcription Factor TGA3 Elevates LCD Expression and H2S Production to Bolster Cr6+ Tolerance in Arabidopsis. Plant J..

[306] Rezayian M., Zarinkamar F. (2023). Nitric Oxide, Calmodulin and Calcium Protein Kinase Interactions in the Response of Brassica Napus to Salinity Stress. Plant Biol..

[307] Tian W., Hou C., Ren Z., Wang C., Zhao F., Dahlbeck D., Hu S., Zhang L., Niu Q., Li L. (2019). A Calmodulin-Gated Calcium Channel Links Pathogen Patterns to Plant Immunity. Nature.

[308] Yin X.M., Huang L.F., Zhang X., Wang M.L., Xu G.Y., Xia X.J. (2015). OsCML4 Improves Drought Tolerance through Scavenging of Reactive Oxygen Species in Rice. J. Plant Biol..

[309] Yoo J.H., Cheong M.S., Park C.Y., Moon B.C., Kim M.C., Kang Y.H., Park H.C., Choi M.S., Lee J.H., Jung W.Y. (2004). Regulation of the Dual Specificity Protein Phosphatase, DsPTP1, through Interactions with Calmodulin*. J. Biol. Chem..

[310] Delumeau O., Paven M.-C.M.-L., Montrichard F., Laval-Martin D.L. (2000). Effects of Short-Term NaCl Stress on Calmodulin Transcript Levels and Calmodulin-Dependent NAD Kinase Activity in Two Species of Tomato. Plant Cell Environ..

[311] Ghorbel M., Zaidi I., Robe E., Ranty B., Mazars C., Galaud J.-P., Hanin M. (2015). The Activity of the Wheat MAP Kinase Phosphatase 1 Is Regulated by Manganese and by Calmodulin. Biochimie.

[312] Borsics T., Webb D., Andeme-Ondzighi C., Staehelin L.A., Christopher D.A. (2007). The Cyclic Nucleotide-Gated Calmodulin-Binding Channel AtCNGC10 Localizes to the Plasma Membrane and Influences Numerous Growth Responses and Starch Accumulation in Arabidopsis Thaliana. Planta.

[313] Yang J., Ji L., Liu S., Jing P., Hu J., Jin D., Wang L., Xie G. (2021). The CaM1-Associated CCaMK–MKK1/6 Cascade Positively Affects Lateral Root Growth via Auxin Signaling under Salt Stress in Rice. J. Exp. Bot..

[314] Shin D., Koo Y.D., Lee J., Lee H., Baek D., Lee S., Cheon C.-I., Kwak S.-S., Lee S.Y., Yun D.-J. (2004). Athb-12, a Homeobox-Leucine Zipper Domain Protein from Arabidopsis Thaliana, Increases Salt Tolerance in Yeast by Regulating Sodium Exclusion. Biochem. Biophys. Res. Commun..

[315] Arazi T., Sunkar R., Kaplan B., Fromm H. (1999). A Tobacco Plasma Membrane Calmodulin-Binding Transporter Confers Ni^2+^ Tolerance and Pb^2+^ Hypersensitivity in Transgenic Plants. Plant J..

[316] Moy A., Czajka K., Michael P., Nkongolo K. (2023). Transcriptome Analysis Reveals Changes in Whole Gene Expression, Biological Process, and Molecular Functions Induced by Nickel in Jack Pine (*Pinus banksiana*). Plants.

