# Supplementary material for: Decrypting Molecular Mechanisms Involved in Counteracting Copper and Nickel Toxicity in Jack Pine (Pinus banksiana) Based on Transcriptomic Analysis
Source: Plants (Basel). 2024 Apr 8;13(7):1042. doi: 10.3390/plants13071042 (PMC11013723; doi:10.3390/plants13071042)
Supplement: Supplementary file 1 [file plants-13-01042-s001.zip › plants-2900603-supplementary.pdf]

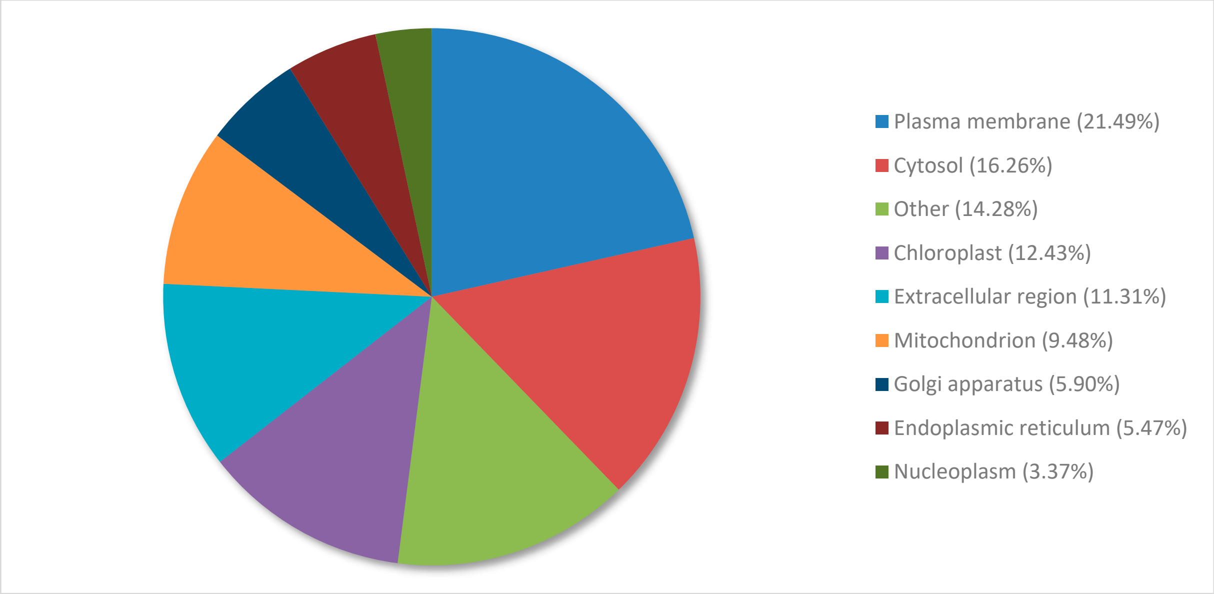

Figure S1. Percent distribution of differentially expressed genes (DEGs) for cell compartment localization when copper resistant (RG) and nickel resistant (RG) were compared. DEGs from copper RG compared to nickel RG were annotated and allocated to terms within the cellular compartment category using Omicsbox/Blast2GO. Terms that had a total percentage of expressed genes lower than 2% were collectively categorized into the term "other."

Table S1. Damage rating scale and plant classification based on reaction to nickel and copper treatments.

% of Leaf area with chlorosis/necrosis	Damage Rating	Genotype Classification
0-10	1	
10-20	2	Resistant (RG)
20-30	3	
30-40	4	
40-50	5	Moderately Susceptible (MSG)
50-60	6	
60-70	7	
70-80	8	Susceptible (SG)
> 80	9	

Table S2. Top 108 upregulated genes in copper RG in comparison to Nickel RG in *Pinus banksiana*

Rank	Gene ID	Cu Res1	Cu Res2	Cu Res3	Ni Res1	Ni Res2	Ni Res3	logFC	Adj. P. value	UniProt Description
**0**	TRINITY_DN2085_c0_g1	29.21	82.28	46.17	0.34	0.57	0.14	7.793	7.10E-05	Predicted Protein
**1**	TRINITY_DN1891_c0_g2	51.01	52.45	17.61	0.04	2.06	0.7	7.247	0.000114	Predicted Protein
**2**	TRINITY_DN8469_c0_g1	43.02	275.4	201.9	1.33	8.3	0.46	6.863	6.98E-05	Acid phosphatase 1, EC 3.1.3.2 (Apase-1(1))
**3**	TRINITY_DN1979_c0_g1	32.13	43.51	47.01	0.21	3.16	0.33	6.702	5.45E-06	Predicted Protein
**4**	TRINITY_DN4373_c0_g1	35.55	56.98	13.12	0.54	0.5	0.36	6.595	8.44E-05	Predicted Protein
**5**	TRINITY_DN5_c0_g1	18.7	16.84	12.27	0.1	0.8	0.23	6.538	0.000106 363	Pleiotropic drug resistance protein 1 (NtPDR1)
**6**	TRINITY_DN5_c0_g1	18.7	16.84	12.27	0.1	0.8	0.23	6.538	0.000106	ABC transporter G family member 32, ABC transporter ABCG.32, AtABCG32 (Pleiotropic drug resistance protein 4) (Protein PERMEABLE CUTICLE 1)
**7**	TRINITY_DN5_c0_g1	18.7	16.84	12.27	0.1	0.8	0.23	6.538	0.000106	ABC transporter G family member 36, OsABCG36 (Pleiotropic drug resistance protein 9, OsPDR9)
**8**	TRINITY_DN5_c0_g1	18.7	16.84	12.27	0.1	0.8	0.23	6.538	0.000106	Pleiotropic drug resistance protein 1 (NpPDR1)
**9**	TRINITY_DN5_c0_g1	18.7	16.84	12.27	0.1	0.8	0.23	6.538	0.000106	ABC transporter G family member 44, OsABCG44 (Pleiotropic drug resistance protein 17, OsPDR17)
**10**	TRINITY_DN5_c0_g1	18.7	16.84	12.27	0.1	0.8	0.23	6.538	0.000106	ABC transporter G family member 31, OsABCG31 (Pleiotropic drug resistance protein 6, OsPDR6)
**11**	TRINITY_DN5_c0_g1	18.7	16.84	12.27	0.1	0.8	0.23	6.538	0.000106	ABC transporter G family member 34, OsABCG34 (Pleiotropic drug resistance protein 10, OsPDR10)
**12**	TRINITY_DN5742_c0_g1	25.47	76.4	14.17	0.6	1.07	0.41	6.162	5.17E-05	Predicted Protein
**13**	TRINITY_DN59895_c0_g1	107.34	136.22	54.47	0.8	4.56	2.36	6.155	1.45E-06	Predicted Protein
**14**	TRINITY_DN51306_c0_g1	32.05	61.43	35.69	0.53	0.7	1.91	6.153	1.48E-06	Predicted Protein
**15**	TRINITY_DN8170_c0_g1	50.21	123.33	300.25	1.81	1.13	8.48	6.150	5.72E-05	Predicted Protein
**16**	TRINITY_DN19606_c0_g1	31.77	21.11	20.22	0.5	1.66	0.29	5.866	4.22E-06	Granule-bound starch synthase 1, chloroplastic/amyloplastic, EC 2.4.1.242 (Granule-bound starch synthase I, GBSS-I)

**17**	TRINITY_DN6541_c0_g1	251.91	62.08	39.98	3.22	2.8	1.68	5.744	4.07E-05	Heavy metal-associated isoprenylated plant protein 20, AtHIP20, AtHIPP20
**18**	TRINITY_DN6541_c0_g1	251.91	62.08	39.98	3.22	2.8	1.68	5.744	4.07E-05	Heavy metal-associated isoprenylated plant protein 26, AtHIP26, AtHIPP26 (Farnesylated protein 6, AtFP6)
**19**	TRINITY_DN47729_c0_g2	7.89	24.87	20.63	0.46	0.23	0.87	5.668	0.000146	Predicted Protein
**20**	TRINITY_DN430_c0_g1	123.72	65.04	52.06	1.02	8.95	2.15	5.441	8.58E-06	Cytochrome P450 86A22, EC 1.14.14.129 (Long-chain acyl-CoA omega-monooxygenase)
**21**	TRINITY_DN1790_c0_g1	9.55	18.4	18.66	0.23	2.08	0.31	5.440	4.00E-05	Receptor-like protein kinase HSL1, EC 2.7.11.1 (Protein HAESA-LIKE1)
**22**	TRINITY_DN1072_c0_g1	97.21	115.92	41.8	4.4	9.29	0.62	5.334	6.56E-05	Predicted Protein
**23**	TRINITY_DN6055_c0_g1	18.79	22.98	21.77	0.56	1.18	0.81	5.330	5.78E-07	Predicted Protein
**24**	TRINITY_DN6813_c0_g1	235.35	117.07	35.45	1.7	10.19	3.65	5.309	6.74E-05	Predicted Protein
**25**	TRINITY_DN11710_c0_g2	150.88	92.2	20.39	1.17	7.66	2.13	5.304	0.000137	Predicted Protein
**26**	TRINITY_DN63319_c0_g1	28.39	21.29	22.62	0.58	1.05	1.41	5.255	8.92E-07	Predicted Protein
**27**	TRINITY_DN82353_c0_g1	13.94	23.06	17.36	0.87	0.98	0.45	5.156	2.89E-06	Predicted Protein
**28**	TRINITY_DN48473_c0_g1	56.5	107.55	80.8	1.68	5.59	4.91	5.085	9.27E-07	Predicted Protein
**29**	TRINITY_DN2130_c0_g1	22.32	44.82	44.69	2.2	1.09	1.68	5.050	4.23E-06	Predicted Protein
**30**	TRINITY_DN1441_c0_g2	340.37	453.06	121.8	13.03	19.89	9.5	4.943	5.01E-06	Predicted Protein
**31**	TRINITY_DN18305_c0_g1	12.58	17.34	7.24	0.21	1.24	0.93	4.934	4.54E-05	Predicted Protein
**32**	TRINITY_DN13702_c0_g1	199.68	359.26	315.24	12.82	34.72	6.66	4.907	2.16E-06	Predicted Protein
**33**	TRINITY_DN21077_c0_g1	104.2	111.9	29.03	1.46	13.71	2.65	4.883	8.29E-05	Predicted Protein
**34**	TRINITY_DN6218_c0_g2	633.56	1516.57	853.55	35.82	29.45	111.35	4.880	6.20E-06	Predicted Protein
**35**	TRINITY_DN234085_c0_g1	76.04	120.68	54.54	4.46	3.78	4.27	4.879	9.57E-07	Predicted Protein
**36**	TRINITY_DN590_c0_g1	200.71	160.06	49.6	4.56	6.07	9.65	4.833	1.59E-05	Predicted Protein
**37**	TRINITY_DN255785_c0_g2	10	9.31	5.83	0.27	0.7	0.49	4.799	4.24E-05	Predicted Protein
**38**	TRINITY_DN107212_c0_g1	73.86	56.2	27.06	2.12	3.39	2.87	4.777	3.19E-06	Predicted Protein
**39**	TRINITY_DN1091_c0_g1	107.98	82.18	33.01	3	8.72	2.4	4.706	1.41E-05	Predicted Protein
**40**	TRINITY_DN4262_c0_g2	6.71	7.64	4.96	0.32	0.6	0.28	4.652	0.000126	Predicted Protein
**41**	TRINITY_DN10240_c0_g1	9.94	8.21	10.71	0.46	0.21	1.82	4.629	8.89E-05	Blue copper protein
**42**	TRINITY_DN103915_c0_g1	7.46	29.74	24.59	0.55	2.53	0.98	4.595	0.000104	Predicted Protein
**43**	TRINITY_DN63319_c0_g2	57.41	39.86	23.94	2.04	2.44	2.92	4.591	3.30E-06	Predicted Protein

**44**	TRINITY_DN5693_c0_g1	315.06	441.27	270.61	9.95	42.87	23.88	4.584	8.61E-07	Predicted Protein
**45**	TRINITY_DN583_c0_g1	533.19	429.99	237.56	15.87	46.05	23.18	4.522	1.05E-06	Photosystem II 22 kDa protein, chloroplastic (CP22)
**46**	TRINITY_DN1967_c0_g1	16.28	29.89	19.85	1.39	0.84	2.46	4.504	1.16E-05	Shikimate O-hydroxycinnamoyltransferase, EC 2.3.1.133 (Hydroxycinnamoyl transferase) (Hydroxycinnamoyl-Coenzyme A shikimate/quinate hydroxycinnamoyltransferase)
**47**	TRINITY_DN13260_c0_g1	11	18.27	23.07	0.85	0.78	1.93	4.502	1.44E-05	Predicted Protein
**48**	TRINITY_DN5923_c0_g1	5.55	9.36	11.52	0.49	1.08	0.31	4.481	5.26E-05	Predicted Protein
**49**	TRINITY_DN1544_c0_g1	13.52	20.43	14.75	0.59	2.03	1.18	4.444	4.04E-06	Cellulose synthase-like protein E6, EC 2.4.1.- (OsCslE6)
**50**	TRINITY_DN5006_c0_g1	8.07	9.8	7.28	0.24	1.58	0.58	4.404	3.12E-05	Cytochrome P450 750A1, EC 1.14.-.- (Cytochrome P450 CYPC)
**51**	TRINITY_DN2833_c0_g1	8.64	16.5	9.06	0.6	0.73	1.13	4.401	1.47E-05	Predicted Protein
**52**	TRINITY_DN139958_c0_g1	17.76	14.28	10.19	1.67	1.29	0.42	4.399	2.89E-05	Predicted Protein
**53**	TRINITY_DN5325_c0_g1	7.04	6.54	4.79	0.32	0.54	0.49	4.393	6.94E-05	Predicted Protein
**54**	TRINITY_DN2317_c0_g1	51.56	73.8	37.5	3.73	3.38	4.66	4.376	1.82E-06	Predicted Protein
**55**	TRINITY_DN4110_c0_g1	10.72	17.48	18.17	0.75	0.9	2	4.356	1.17E-05	Predicted Protein
**56**	TRINITY_DN792_c0_g2	397.04	322.71	190.6	14.08	73.46	10.46	4.349	2.23E-05	Granule-bound starch synthase 1, chloroplastic/amyloplastic, EC 2.4.1.242 (Granule-bound starch synthase I, GBSS-I)
**57**	TRINITY_DN3113_c0_g1	19.55	36.23	25.85	3.29	2.48	1.02	4.291	1.41E-05	Predicted Protein
**58**	TRINITY_DN62635_c0_g1	11.04	20.42	16.53	1.01	1.74	1.02	4.276	4.59E-06	Predicted Protein
**59**	TRINITY_DN20024_c0_g1	1387.36	3245.98	1849.75	109	181.45	219.45	4.259	8.19E-07	Predicted Protein
**60**	TRINITY_DN51306_c2_g1	456.91	1001.17	567.06	33.89	34.09	122.11	4.241	1.65E-05	Predicted Protein
**61**	TRINITY_DN452_c1_g1	7.49	12.56	6.92	0.27	1.63	0.82	4.237	4.90E-05	Receptor protein-tyrosine kinase CEPR1, EC 2.7.10.1 (Protein C-TERMINALLY ENCODED PEPTIDE RECEPTOR 1) (Protein XYLEM INTERMIXED WITH PHLOEM 1)
**62**	TRINITY_DN452_c1_g1	7.49	12.56	6.92	0.27	1.63	0.82	4.237	4.90E-05	Leucine-rich repeat receptor-like kinase protein SUNN, EC 2.7.11.1 (Protein SUPER NUMERIC NODULES)

**63**	TRINITY_DN13950_c0_g1	8.91	12.29	10.7	0.56	2.45	0.46	4.217	2.52E-05	Predicted Protein
**64**	TRINITY_DN8956_c0_g1	22.9	32.53	59.54	3.34	3.17	2.24	4.206	1.00E-05	Predicted Protein
**65**	TRINITY_DN557_c0_g1	351.25	695.93	342.88	24.36	46.66	44.49	4.195	8.54E-07	Fructose-bisphosphate aldolase 2, chloroplastic, AtFBA2, EC 4.1.2.13
**66**	TRINITY_DN557_c0_g1	351.25	695.93	342.88	24.36	46.66	44.49	4.195	8.54E-07	Fructose-bisphosphate aldolase, chloroplastic, EC 4.1.2.13
**67**	TRINITY_DN557_c0_g1	351.25	695.93	342.88	24.36	46.66	44.49	4.195	8.54E-07	Fructose-bisphosphate aldolase 3, chloroplastic, AtFBA3, EC 4.1.2.13 (Protein PIGMENT DEFECTIVE 345)
**68**	TRINITY_DN557_c0_g1	351.25	695.93	342.88	24.36	46.66	44.49	4.195	8.54E-07	Fructose-bisphosphate aldolase, chloroplastic, EC 4.1.2.13 (Chloroplastic aldolase, AldP)
**69**	TRINITY_DN13626_c0_g1	57.47	45.59	25.35	1.36	6.65	4.7	4.191	1.95E-05	Predicted Protein
**70**	TRINITY_DN5309_c0_g1	7.16	12.51	6.03	0.32	0.99	1.13	4.179	4.72E-05	Probable E3 ubiquitin-protein ligase RHY1A, EC 2.3.2.27 (RING-H2 finger Y1a) (RING-H2 zinc finger protein RHY1a) (RING-type E3 ubiquitin transferase RHY1A)
**71**	TRINITY_DN125498_c1_g1	121.27	373.21	247.23	9.74	17.65	44.82	4.123	4.22E-05	Predicted Protein
**72**	TRINITY_DN963_c0_g2	69.63	60.55	89.57	3.21	19.61	4.19	4.110	1.57E-05	Metal-nicotianamine transporter YSL3 (Protein YELLOW STRIPE LIKE 3, AtYSL3)
**73**	TRINITY_DN963_c0_g2	69.63	60.55	89.57	3.21	19.61	4.19	4.110	1.57E-05	Probable metal-nicotianamine transporter YSL7 (Protein YELLOW STRIPE LIKE 7, AtYSL7)
**74**	TRINITY_DN963_c0_g2	69.63	60.55	89.57	3.21	19.61	4.19	4.110	1.57E-05	Probable metal-nicotianamine transporter YSL12 (Protein YELLOW STRIPE LIKE 12, OsYSL12)
**75**	TRINITY_DN963_c0_g2	69.63	60.55	89.57	3.21	19.61	4.19	4.110	1.57E-05	Probable metal-nicotianamine transporter YSL14 (Protein YELLOW STRIPE LIKE 14, OsYSL14)
**76**	TRINITY_DN963_c0_g2	69.63	60.55	89.57	3.21	19.61	4.19	4.110	1.57E-05	Probable metal-nicotianamine transporter YSL11 (Protein YELLOW STRIPE LIKE 11, OsYSL11)
**77**	TRINITY_DN963_c0_g2	69.63	60.55	89.57	3.21	19.61	4.19	4.110	1.57E-05	Probable metal-nicotianamine transporter YSL9 (Protein YELLOW STRIPE LIKE 9, OsYSL9)

**78**	TRINITY_DN963_c0_g2	69.63	60.55	89.57	3.21	19.61	4.19	4.110	1.57E-05	Probable metal-nicotianamine transporter YSL16 (Protein YELLOW STRIPE LIKE 16, OsYSL16)
**79**	TRINITY_DN52249_c0_g3	7.28	5.78	4.03	0.21	1.21	0.51	4.088	0.000135	Predicted Protein
**80**	TRINITY_DN3604_c0_g1	5.48	6.5	4.61	0.29	1.04	0.41	4.075	6.63E-05	Predicted Protein
**81**	TRINITY_DN1217_c0_g1	48.16	71.15	146	2.86	8.85	14.44	4.074	5.54E-05	Putative anthocyanidin reductase, GbANR, EC 1.3.1.-
**82**	TRINITY_DN6386_c0_g1	11.97	34.03	18.9	1.71	0.92	3.86	4.059	0.000125	Predicted Protein
**83**	TRINITY_DN26976_c0_g1	82.95	53.31	76.71	1.91	16.63	8.7	4.054	4.59E-05	Aspartyl protease family protein At5g10770, EC 3.4.23.-
**84**	TRINITY_DN26976_c0_g1	82.95	53.31	76.71	1.91	16.63	8.7	4.054	4.59E-05	Protein ASPARTIC PROTEASE IN GUARD CELL 1, AtASPG1, EC 3.4.23.-
**85**	TRINITY_DN10188_c0_g1	7.31	6.61	3.43	0.4	0.82	0.4	4.048	0.000139	Cytochrome P450 750A1, EC 1.14.-.- (Cytochrome P450 CYPC)
**86**	TRINITY_DN5205_c0_g1	48.67	71.45	57.26	2.98	7.51	7.24	4.046	1.56E-06	Predicted Protein
**87**	TRINITY_DN224_c0_g1	15.09	15.45	6.33	0.48	1.72	1.63	4.031	6.75E-05	Subtilisin-like serine-protease S, SbtS, Subtilase S, EC 3.4.21.-
**88**	TRINITY_DN224_c0_g1	15.09	15.45	6.33	0.48	1.72	1.63	4.031	6.75E-05	Subtilisin-like protease SBT3.3, EC 3.4.21.- (Subtilase subfamily 3 member 3, AtSBT3.3)
**89**	TRINITY_DN224_c0_g1	15.09	15.45	6.33	0.48	1.72	1.63	4.031	6.75E-05	Subtilisin-like protease SBT3.5, EC 3.4.21.- (Subtilase subfamily 3 member 5, AtSBT3.5)
**90**	TRINITY_DN224_c0_g1	15.09	15.45	6.33	0.48	1.72	1.63	4.031	6.75E-05	Subtilisin-like protease SBT3.15, EC 3.4.21.- (Subtilase subfamily 3 member 15, AtSBT3.15)
**91**	TRINITY_DN1243_c0_g2	21.49	20.9	7.13	1.45	1.46	1.3	4.020	5.63E-05	Predicted Protein
**92**	TRINITY_DN3029_c0_g1	35.14	40.46	12.44	1.59	2.74	3.99	3.988	5.31E-05	Predicted Protein
**93**	TRINITY_DN54045_c0_g2	8.44	9.51	14.88	1.03	0.93	1.06	3.973	1.50E-05	Predicted Protein
**94**	TRINITY_DN5990_c0_g1	5.66	6.63	3.4	0.43	0.59	0.47	3.958	0.000133	Predicted Protein
**95**	TRINITY_DN2642_c0_g1	4.84	6.48	6.06	0.38	1.22	0.39	3.949	5.65E-05	Predicted Protein
**96**	TRINITY_DN3360_c0_g2	7.55	14.02	7.56	0.37	2.13	1.09	3.931	6.33E-05	Predicted Protein
**97**	TRINITY_DN28667_c0_g1	10.25	13.09	5.92	0.45	3.23	0.64	3.900	0.000142	Predicted Protein
**98**	TRINITY_DN1344_c0_g1	13.88	10.7	7.37	0.84	2.55	0.55	3.899	4.57E-05	Mannose-1-phosphate guanylyltransferase 1, EC 2.7.7.13 (GDP-mannose pyrophosphorylase 1)

										(Protein CYTOKINESIS DEFECTIVE 1) (Protein EMBRYO DEFECTIVE 101) (Protein HYPERSENSITIVE TO AMMONIUM ION 1) (Protein SENSITIVE TO OZONE 1) (Protein VITAMIN C DEFECTIVE 1)
**99**	TRINITY_DN7300_c0_g1	10.65	10.03	24.85	1.47	0.91	2.05	3.878	8.78E-05	Predicted Protein
**100**	TRINITY_DN4544_c0_g1	32.45	29.29	20.57	1.27	2.8	6.6	3.873	3.56E-05	Predicted Protein
**101**	TRINITY_DN3984_c0_g2	6.33	5.62	5.47	0.29	2.02	0.38	3.872	0.000121	Predicted Protein
**102**	TRINITY_DN3497_c0_g1	6.98	11.31	5.89	1.03	0.97	0.49	3.871	4.60E-05	Predicted Protein
**103**	TRINITY_DN35587_c0_g1	4.4	6.71	6.78	0.5	0.74	0.63	3.825	4.04E-05	Glyoxylase I 4, AtGLXI-like;4, AtGLYI4
**104**	TRINITY_DN26392_c0_g2	28.62	15.31	12.83	1.28	5.23	1.1	3.814	6.93E-05	Predicted Protein
**105**	TRINITY_DN57465_c0_g1	6.53	8.2	4.79	0.31	1.82	0.64	3.813	9.59E-05	Predicted Protein
**106**	TRINITY_DN262_c0_g2	8.8	20.03	10.55	1.16	1.57	1.39	3.788	2.18E-05	Polygalacturonase ADPG2, AtADPG2, PG ADPG2, EC 3.2.1.15 (Pectinase ADPG2) (Protein ARABIDOPSIS DEHISCENCE ZONE POLYGALACTURONASE 2)
**107**	TRINITY_DN262_c0_g2	8.8	20.03	10.55	1.16	1.57	1.39	3.788	2.18E-05	Probable polygalacturonase At1g80170, PG, EC 3.2.1.15 (Pectinase At1g80170)
**108**	TRINITY_DN262_c0_g2	8.8	20.03	10.55	1.16	1.57	1.39	3.788	2.18E-05	Polygalacturonase, PG, EC 3.2.1.15 (Pectinase)

Table S3. Top 112 downregulated genes in copper RG in comparison to Nickel RG in *Pinus banksiana*

Rank	Gene ID	Cu Res1	Cu Res2	Cu Res3	Ni Res1	Ni Res2	Ni Res3	logFC	Adj. P. value	UniProt Description
**0**	TRINITY_DN43547_c0_g1	0	0	0	24.15	39.01	23.93	-10.382	6.42E-05	Predicted Protein
**1**	TRINITY_DN1456_c0_g1	1.37	0	1.34	306.48	253.1	155.47	-9.527	0.000128719	Predicted Protein
**2**	TRINITY_DN2126_c0_g1	1.31	0.24	0.4	954.55	193.03	448.85	-9.030	4.26E-05	UDP-glycosyltransferase 75C1, Abscisic acid beta-glucosyltransferase, Indole-3-acetate beta-glucosyltransferase, SlUGT75C1, EC 2.4.1.121, EC 2.4.1.263
**3**	TRINITY_DN5965_c0_g1	0.28	0.12	1.02	159.21	70.92	203.21	-8.429	5.08E-05	Predicted Protein
**4**	TRINITY_DN5240_c1_g1	0.06	0.2	0.38	75.64	57.88	47.63	-7.894	0.000131675	Predicted Protein
**5**	TRINITY_DN2786_c0_g1	7.97	1.28	0.09	767.81	197.57	545.86	-7.817	0.000147002	Predicted Protein
**6**	TRINITY_DN7685_c0_g1	0.35	0.17	0.18	69.15	75.78	88.19	-7.718	1.68E-05	Predicted Protein
**7**	TRINITY_DN3685_c0_g2	7.54	1.2	0.09	524.13	169.45	298.36	-7.315	0.000146393	Copia protein (Gag-int-pol protein) [Cleaved into: Copia VLP protein; Copia protease, EC 3.4.23.-]
**8**	TRINITY_DN2463_c0_g1	5.21	0.97	0.68	301.76	196.16	568.86	-7.021	8.44E-06	Predicted Protein
**9**	TRINITY_DN2040_c0_g1	28.51	6	12.87	3943.91	1043.09	3644.25	-6.950	1.04E-05	Predicted Protein
**10**	TRINITY_DN5795_c0_g1	13.6	5.09	0.4	753.52	420.03	412.9	-6.580	5.36E-05	Predicted Protein
**11**	TRINITY_DN735_c0_g1	5.88	1.92	0.26	401.38	124.53	198.21	-6.422	9.90E-05	Predicted Protein
**12**	TRINITY_DN41085_c0_g1	1.46	0.55	8.2	230.93	100.32	461.71	-6.396	8.67E-05	Predicted Protein
**13**	TRINITY_DN5240_c0_g1	0.4	0.48	1.48	86.62	78.96	59.64	-6.375	1.11E-06	Protein TIFY 10b, OsTIFY10b (Jasmonate ZIM domain-containing protein 7, OsJAZ7) (OsJAZ6)
**14**	TRINITY_DN17_c0_g2	0.65	0.15	0.58	50.63	58.32	26.97	-6.307	1.89E-05	RING-H2 finger protein ATL60, EC 2.3.2.27 (RING-type E3 ubiquitin transferase ATL60)
**15**	TRINITY_DN800_c1_g1	2.53	0.34	0.33	127.84	44.55	121.22	-6.264	0.000103509	Predicted Protein
**16**	TRINITY_DN1755_c0_g1	0.73	0.19	0.22	37.36	42.04	37.53	-6.143	1.54E-05	Predicted Protein
**17**	TRINITY_DN1067_c0_g1	0.21	0.63	2.66	74.75	51.13	62.5	-6.138	1.50E-05	Predicted Protein

**18**	TRINITY_DN4963_c0_g1	1.2	1.08	0.26	107.91	63.25	101.25	-6.114	6.05E-06	Predicted Protein
**19**	TRINITY_DN3889_c0_g1	8.77	0.85	2.51	414.11	167.54	300.23	-6.071	3.22E-05	Predicted Protein
**20**	TRINITY_DN6289_c0_g1	0.34	0.08	0.38	23.79	21.01	21.41	-5.988	0.000135072	Predicted Protein
**21**	TRINITY_DN6111_c0_g1	0.47	0.11	0.98	29.31	29.12	29.02	-5.932	1.49E-05	Predicted Protein
**22**	TRINITY_DN4477_c0_g1	1.32	1.08	0.77	67.92	163.48	92.08	-5.924	1.74E-07	Predicted Protein
**23**	TRINITY_DN71807_c0_g1	0.75	0.09	1.03	29.61	23.8	49.04	-5.903	8.02E-05	Predicted Protein
**24**	TRINITY_DN971_c0_g1	0.32	0.18	3.12	40.71	30.9	35.98	-5.891	3.15E-05	1-aminocyclopropane-1-carboxylate synthase 7, ACC synthase 7, EC 4.4.1.14 (S-adenosyl-L-methionine methylthioadenosine-lyase 7)
**25**	TRINITY_DN971_c0_g1	0.32	0.18	3.12	40.71	30.9	35.98	-5.891	3.15E-05	1-aminocyclopropane-1-carboxylate synthase, ACC synthase, EC 1.4.-.-, EC 4.4.1.14 (S-adenosyl-L-methionine methylthioadenosine-lyase)
**26**	TRINITY_DN971_c0_g1	0.32	0.18	3.12	40.71	30.9	35.98	-5.891	3.15E-05	1-aminocyclopropane-1-carboxylate synthase 6, ACC synthase 6, EC 4.4.1.14 (S-adenosyl-L-methionine methylthioadenosine-lyase 6)
**27**	TRINITY_DN971_c0_g1	0.32	0.18	3.12	40.71	30.9	35.98	-5.891	3.15E-05	1-aminocyclopropane-1-carboxylate synthase 3, ACC synthase 3, EC 4.4.1.14 (Le-ACS3, ACS-3) (S-adenosyl-L-methionine methylthioadenosine-lyase 3)
**28**	TRINITY_DN971_c0_g1	0.32	0.18	3.12	40.71	30.9	35.98	-5.891	3.15E-05	1-aminocyclopropane-1-carboxylate synthase 1, ACC synthase 1, EC 4.4.1.14 (S-adenosyl-L-methionine methylthioadenosine-lyase)
**29**	TRINITY_DN756_c0_g1	7.06	0.88	8.25	578.57	195.91	235.86	-5.769	7.82E-05	Dirigent protein 21, AtDIR21
**30**	TRINITY_DN6061_c0_g1	3.75	2.9	7.44	355.22	178.84	637.66	-5.730	1.01E-05	Predicted Protein
**31**	TRINITY_DN1472_c0_g1	12.73	1.55	0.53	212.32	210.54	174.12	-5.729	5.80E-05	Triacylglycerol lipase OBL1, EC 3.1.1.- (Oil body lipase 1, NtOBL1)
**32**	TRINITY_DN6866_c0_g1	0.23	0.29	1.89	32.98	20.01	40.66	-5.649	4.37E-05	Predicted Protein
**33**	TRINITY_DN5723_c0_g1	5.47	0.91	0.48	147.25	109.75	89.9	-5.649	2.65E-05	Predicted Protein
**34**	TRINITY_DN7784_c0_g1	7.88	7.39	4.37	322.86	341.39	1073.69	-5.627	4.26E-06	Predicted Protein
**35**	TRINITY_DN5965_c1_g1	53.22	6.07	6.47	1173.34	760.7	1106.06	-5.627	2.34E-05	Predicted Protein

**36**	TRINITY_DN9734_c0_g1	0.08	0.49	1.59	23.73	24.47	17.56	-5.551	5.09E-05	Predicted Protein
**37**	TRINITY_DN565_c0_g1	2.22	1.22	1.18	93.13	68.83	155.62	-5.423	3.99E-06	Peroxidase 12, Atperox P12, EC 1.11.1.7 (ATP4a) (PRXR6)
**38**	TRINITY_DN565_c0_g1	2.22	1.22	1.18	93.13	68.83	155.62	-5.423	3.99E-06	Cationic peroxidase SPC4, EC 1.11.1.7
**39**	TRINITY_DN3459_c0_g1	2.36	0.56	3.89	101.88	77.2	153.38	-5.410	2.03E-05	Predicted Protein
**40**	TRINITY_DN2419_c0_g1	0.45	0.26	1.32	30.69	23.95	31.41	-5.361	1.25E-05	Predicted Protein
**41**	TRINITY_DN350_c0_g2	1.67	1.44	1.31	130.63	158.57	38.54	-5.351	1.57E-05	Benzyl alcohol O-benzoyltransferase, EC 2.3.1.196 (Benzoyl coenzyme A:benzyl alcohol benzoyl transferase)
**42**	TRINITY_DN1483_c0_g1	2.06	0.14	1.59	46.18	45.16	46.95	-5.347	8.11E-05	Predicted Protein
**43**	TRINITY_DN3428_c0_g2	11.13	4.13	5.23	540.1	213.89	516.93	-5.345	1.14E-05	Predicted Protein
**44**	TRINITY_DN38763_c0_g1	1.24	0.74	2.14	88.25	171.45	27.6	-5.333	5.55E-05	Glyoxylase I 4, AtGLXI-like;4, AtGLYI4
**45**	TRINITY_DN4971_c0_g2	11.63	2.63	2.86	386.29	249.26	229.67	-5.330	7.46E-06	Predicted Protein
**46**	TRINITY_DN1869_c0_g1	0.2	0.42	0.39	17.82	15.6	24.1	-5.277	6.23E-05	Predicted Protein
**47**	TRINITY_DN6746_c0_g1	0.98	1.33	0.37	50.95	34.06	77.5	-5.265	1.96E-05	Predicted Protein
**48**	TRINITY_DN3022_c0_g2	0.68	0.44	0.73	35.78	28.02	41.18	-5.261	5.46E-06	Predicted Protein
**49**	TRINITY_DN709_c0_g1	0.43	0.17	0.44	22.94	16.65	13.69	-5.231	0.000125	Predicted Protein
**50**	TRINITY_DN14557_c0_g1	13.84	7.83	6.14	671.5	289.97	665.67	-5.219	6.57E-06	Linoleate 9S-lipoxygenase A, EC 1.13.11.58 (Lipoxygenase A)
**51**	TRINITY_DN703_c0_g1	0.68	0.39	0.13	26.88	23.74	22.23	-5.206	6.39E-05	Auxin-responsive protein SAUR32 (Protein ABOLISHED APICAL HOOK MAINTENANCE 1) (Protein SMALL AUXIN UP RNA 32)
**52**	TRINITY_DN9217_c0_g2	0.28	0.3	0.4	16.06	31.56	10.18	-5.167	0.000108	Predicted Protein
**53**	TRINITY_DN3069_c0_g1	6.25	3.02	1.51	310.77	133.21	112.57	-5.095	2.97E-05	Predicted Protein
**54**	TRINITY_DN37281_c0_g1	0.4	0.73	0.64	46.45	19.21	27.73	-5.062	0.000061	Predicted Protein
**55**	TRINITY_DN2454_c0_g2	0.58	0.38	0.49	24.25	22.33	25.85	-5.023	7.54E-06	Predicted Protein
**56**	TRINITY_DN883_c0_g1	3.03	2.1	3.18	207.9	49.7	228.58	-5.004	0.000138	Class V chitinase, AtChiC, EC 3.2.1.14, EC 3.2.1.200
**57**	TRINITY_DN883_c0_g1	3.03	2.1	3.18	207.9	49.7	228.58	-5.004	0.000138	Class V chitinase CHIT5b, MtCHIT5b, EC 3.2.1.14
**58**	TRINITY_DN9201_c0_g1	6.29	2.46	13.2	471.59	149.72	307.61	-5.002	6.59E-05	Predicted Protein

**59**	TRINITY_DN3152_c0_g1	8.05	2.08	3.45	254.41	122.04	221.09	-4.989	1.71E-05	Predicted Protein
**60**	TRINITY_DN481_c0_g1	1.02	0.41	3.08	55.39	66.11	31.34	-4.983	2.66E-05	RING-H2 finger protein ATL2, EC 2.3.2.27 (Protein ARABIDOPSIS TOXICOS EN LEVADURA 2, Protein ATL2) (RING-type E3 ubiquitin transferase ATL2)
**61**	TRINITY_DN481_c0_g1	1.02	0.41	3.08	55.39	66.11	31.34	-4.983	2.66E-05	RING-H2 finger protein ATL60, EC 2.3.2.27 (RING-type E3 ubiquitin transferase ATL60)
**62**	TRINITY_DN12730_c0_g1	3.1	1.53	0.39	49.37	106.18	56.02	-4.966	1.36E-05	Predicted Protein
**63**	TRINITY_DN3537_c0_g1	0.97	0.36	0.32	29.67	31.28	18.67	-4.945	2.13E-05	Predicted Protein
**64**	TRINITY_DN36982_c0_g1	0.96	1.94	2.77	31.59	189.32	83.11	-4.937	2.58E-05	Predicted Protein
**65**	TRINITY_DN350_c0_g3	0.68	1.13	0.56	49.85	47.92	20.65	-4.911	1.63E-05	Benzyl alcohol O-benzoyltransferase, EC 2.3.1.196 (Benzoyl coenzyme A:benzyl alcohol benzoyl transferase)
**66**	TRINITY_DN7061_c1_g1	12.73	4.47	0.85	158.35	218.82	172.81	-4.899	5.55E-05	Predicted Protein
**67**	TRINITY_DN34079_c0_g1	2.25	1.33	1.66	139.04	63.67	49.4	-4.844	0.000024	Predicted Protein
**68**	TRINITY_DN2420_c0_g1	2.42	0.46	0.45	35.9	23.83	61.64	-4.820	0.000136	Predicted Protein
**69**	TRINITY_DN2309_c2_g1	3.77	4	3.12	266.25	96.82	148.11	-4.808	0.000015	Predicted Protein
**70**	TRINITY_DN104547_c0_g 1	7.3	2.88	3.17	238.4	83.01	249.46	-4.753	5.79E-05	Predicted Protein
**71**	TRINITY_DN2423_c0_g1	4.25	0.64	2.98	104.81	46.82	107.91	-4.737	0.000133	Protein TIFY 9 (Jasmonate ZIM domain-containing protein 10) (Protein JASMONATE-ASSOCIATED 1) (Protein JAZ10)
**72**	TRINITY_DN2936_c0_g1	4.11	1.09	0.71	80.77	49.47	64.72	-4.714	3.89E-05	F-box protein At1g67340
**73**	TRINITY_DN3428_c0_g1	8.15	5.52	8.95	405.93	189.91	322.41	-4.701	5.99E-06	Probable linoleate 9S-lipoxygenase 4, EC 1.13.11.58 (Root lipoxygenase)
**74**	TRINITY_DN3428_c0_g1	8.15	5.52	8.95	405.93	189.91	322.41	-4.701	5.99E-06	Linoleate 9S-lipoxygenase A, EC 1.13.11.58 (Lipoxygenase A)
**75**	TRINITY_DN2679_c0_g1	0.64	0.51	0.33	25.2	13.44	26.94	-4.690	0.000109	Predicted Protein
**76**	TRINITY_DN1630_c0_g1	1.33	0.31	0.8	30.88	27.11	23.21	-4.666	0.000025	Predicted Protein
**77**	TRINITY_DN662_c0_g1	18.61	3.39	4.79	297.28	215.67	292.16	-4.661	2.17E-05	Potassium transporter 5 (OsHAK5)
**78**	TRINITY_DN662_c0_g1	18.61	3.39	4.79	297.28	215.67	292.16	-4.661	2.17E-05	Potassium transporter 1 (OsHAK1)

**79**	TRINITY_DN11443_c0_g1	1.61	1.82	0.7	65.72	28.76	74.44	-4.651	5.76E-05	Predicted Protein
**80**	TRINITY_DN528_c0_g1	0.38	0.52	0.46	11.9	28.25	15.99	-4.628	1.17E-05	Phospholipase A1-Igamma1, chloroplastic, EC 3.1.1.- (DAD1-like lipase 4)
**81**	TRINITY_DN528_c0_g1	0.38	0.52	0.46	11.9	28.25	15.99	-4.628	1.17E-05	Phospholipase A1-Ialpha2, chloroplastic, EC 3.1.1.- (DAD1-like lipase 5)
**82**	TRINITY_DN528_c0_g1	0.38	0.52	0.46	11.9	28.25	15.99	-4.628	1.17E-05	Phospholipase A1-Igamma3, chloroplastic, EC 3.1.1.- (DAD1-like lipase 2)
**83**	TRINITY_DN9407_c0_g1	5.03	1.34	1.61	57.25	111.91	98.46	-4.605	5.9E-06	Predicted Protein
**84**	TRINITY_DN2515_c0_g1	8.97	2.8	1.1	140.76	123.23	98.31	-4.585	3.63E-05	Probable disease resistance protein At5g04720
**85**	TRINITY_DN5337_c0_g1	0.18	0.61	1.85	15.76	16.44	20.55	-4.551	8.04E-05	Predicted Protein
**86**	TRINITY_DN5340_c0_g1	3.12	0.37	0.34	27.6	47.12	22.31	-4.546	0.000131	Predicted Protein
**87**	TRINITY_DN16888_c0_g1	1.21	0.47	0.16	20.12	30.42	16.05	-4.523	5.85E-05	Predicted Protein
**88**	TRINITY_DN16040_c0_g1	2.49	0.68	0.76	39.04	44.31	36.54	-4.500	1.05E-05	Predicted Protein
**89**	TRINITY_DN345_c1_g1	22.4	4.62	15.75	490.53	255.64	488.01	-4.464	4.82E-05	Predicted Protein
**90**	TRINITY_DN7436_c0_g2	24.8	30.11	10.46	481.81	599.66	1125.65	-4.460	4.88E-06	Predicted Protein
**91**	TRINITY_DN22831_c0_g2	5.06	2.53	5.2	150.2	59.44	260.42	-4.428	0.000135	Predicted Protein
**92**	TRINITY_DN3878_c1_g1	1.79	0.87	1.35	39.5	57.78	30.4	-4.396	3.72E-06	Predicted Protein
**93**	TRINITY_DN9660_c0_g1	0.46	0.24	1.22	14.82	18.95	9.21	-4.364	6.54E-05	Predicted Protein
**94**	TRINITY_DN2724_c0_g1	3.12	1.88	2.84	84.24	77.44	75.63	-4.332	1.53E-06	Predicted Protein
**95**	TRINITY_DN750_c0_g1	15.85	2.67	2.14	135.54	192.95	114.14	-4.325	6.22E-05	Probable disease resistance protein At5g04720
**96**	TRINITY_DN2509_c0_g1	0.27	0.73	1.18	12.41	14.6	25.02	-4.270	0.000075	Predicted Protein
**97**	TRINITY_DN8616_c0_g1	1.97	1.48	1.13	56	34.08	47.26	-4.260	1.28E-05	Predicted Protein
**98**	TRINITY_DN7743_c0_g2	1.28	0.39	0.61	25.31	13.79	23.91	-4.258	0.000129	Probable phospholipid hydroperoxide glutathione peroxidase, PHGPx, EC 1.11.1.12
**99**	TRINITY_DN399_c0_g1	15.58	5.85	7.45	337.3	126.19	404.82	-4.250	9.96E-05	Predicted Protein
**100**	TRINITY_DN2157_c0_g1	5.52	6.21	4.61	301.86	102.86	127.49	-4.250	4.57E-05	Predicted Protein
**101**	TRINITY_DN9843_c0_g1	2.1	1.4	4.76	70.86	38.88	96.24	-4.181	7.29E-05	Predicted Protein
**102**	TRINITY_DN102451_c0_g 1	0.86	0.49	0.5	10.88	15.85	28.2	-4.144	6.49E-05	Predicted Protein

**103**	TRINITY_DN1644_c0_g3	0.77	0.66	1.22	25.94	13.95	32.04	-4.139	8.63E-05	NAC transcription factor 56, AtNAC056 (NAC domain-containing protein 2, AtNAC2) (Protein NAC-REGULATED SEED MORPHOLOGY 1)
**104**	TRINITY_DN3401_c0_g1	2.04	0.88	1.07	37.44	30.11	31.61	-4.072	1.42E-05	Disease resistance protein L6 (NAD(+) hydrolase L6, EC 3.2.2.6) (NADP(+) hydrolase L6, EC 3.2.2.-)
**105**	TRINITY_DN3401_c0_g1	2.04	0.88	1.07	37.44	30.11	31.61	-4.072	1.42E-05	Disease resistance protein Roq1 (NAD(+) hydrolase RPV1, EC 3.2.2.6) (Recognition of XopQ 1 protein)
**106**	TRINITY_DN1002_c0_g1	1.9	2.6	5.47	69.19	51.5	114.83	-4.055	0.000034	Probable phospholipid hydroperoxide glutathione peroxidase, PHGPx, EC 1.11.1.12 (Salt-associated protein)
**107**	TRINITY_DN1002_c0_g1	1.9	2.6	5.47	69.19	51.5	114.83	-4.055	0.000034	Probable phospholipid hydroperoxide glutathione peroxidase, PHGPx, EC 1.11.1.12
**108**	TRINITY_DN34284_c1_g1	0.82	2.53	4.23	57.32	47.31	45.66	-4.044	4.39E-05	Probable phospholipid hydroperoxide glutathione peroxidase, PHGPx, EC 1.11.1.12
**109**	TRINITY_DN13745_c0_g1	14.93	3.08	2.83	115.9	156.73	125.21	-4.027	4.08E-05	Predicted Protein
**110**	TRINITY_DN36614_c0_g1	2.31	0.47	0.58	16.13	30.43	23.06	-4.002	6.45E-05	Predicted Protein
**111**	TRINITY_DN76829_c0_g1	0.6	0.75	2.06	16.59	19.19	35.57	-4.001	6.46E-05	Predicted Protein
**112**	TRINITY_DN162544_c0_g1	7.49	3.93	1.44	58.14	156.51	81.13	-3.986	3.16E-05	Probable phospholipid hydroperoxide glutathione peroxidase, PHGPx, EC 1.11.1.12

Table S4. Top 40 downregulated genes in copper SG in comparison to nickel SG in *Pinus banksiana*

Rank	Gene ID	Cu Sus1	Cu Sus2	Cu Sus3	Ni Sus1	Ni Sus2	Ni Sus3	logFC	Adj. P. value	UniProt Description
**0**	TRINITY_DN343_c0_g1	1.96	1.42	0.92	54.19	45.61	75.54	-5.26455	7.48E-06	Probable disease resistance protein At4g33300
**1**	TRINITY_DN1105_c0_g1	3.27	4.87	2.07	76.5	72.45	109.99	-4.74021	8.13E-07	Probable calcium-binding protein CML24 (Calmodulin-like protein 24)
**2**	TRINITY_DN1140_c1_g1	4.64	8.22	3.13	95.64	64.13	116.13	-4.21256	7.05E-06	Heavy metal-associated isoprenylated plant protein 39, AtHIP39
**3**	TRINITY_DN9069_c0_g1	3.08	3.94	1.43	39	35.11	55.72	-4.0072	7.49E-06	Predicted Protein
**4**	TRINITY_DN26455_c0_g1	5.55	5.56	3.67	47.82	81.22	105.73	-3.96197	1.86E-06	Predicted Protein
**5**	TRINITY_DN709_c0_g1	3.48	4.83	1.97	36.9	41.46	63.36	-3.84112	4.28E-06	Predicted Protein
**6**	TRINITY_DN24318_c0_g1	4.75	2.79	4.67	40.3	50.05	80.73	-3.79817	8.01E-06	Predicted Protein
**7**	TRINITY_DN2118_c0_g1	5.31	19.73	5.94	91.04	111.37	142.16	-3.79689	2.20E-06	Predicted Protein
**8**	TRINITY_DN66111_c1_g1	14.78	24.19	24.59	172.94	234.1	371.3	-3.66779	1.60E-06	Predicted Protein
**9**	TRINITY_DN9317_c0_g1	9.85	29.45	5.37	142.89	125.63	137.64	-3.56924	7.93E-06	Predicted Protein
**10**	TRINITY_DN25789_c0_g1	5.35	15.72	3.93	67.56	76.93	92.73	-3.54421	3.52E-06	Predicted Protein
**11**	TRINITY_DN2751_c0_g1	12.73	8.43	4.46	77.66	76.97	116.69	-3.4682	4.60E-06	Predicted Protein
**12**	TRINITY_DN1481_c0_g1	31.61	46.43	12.53	248.24	432.11	226.95	-3.44461	5.91E-06	Predicted Protein
**13**	TRINITY_DN37281_c0_g1	6.17	13.07	6.21	74.35	73.03	101.82	-3.42972	1.93E-06	Predicted Protein
**14**	TRINITY_DN11462_c0_g1	15.98	31.57	16.82	122.24	278.4	208.28	-3.29128	1.68E-06	Predicted Protein
**15**	TRINITY_DN3306_c0_g2	18.25	34.56	13.19	154.79	169.1	263.27	-3.27608	1.59E-06	Calcium-binding protein KIC (KCBP-interacting calcium-binding protein)
**16**	TRINITY_DN4522_c0_g1	12.58	21.21	9.5	81.86	98.59	152.54	-3.01301	3.71E-06	Predicted Protein
**17**	TRINITY_DN729_c0_g1	85.61	37.04	35.87	381.71	394.83	348.76	-2.92258	4.38E-06	Alcohol dehydrogenase 2, EC 1.1.1.1
**18**	TRINITY_DN729_c0_g1	85.61	37.04	35.87	381.71	394.83	348.76	-2.92258	4.38E-06	Alcohol dehydrogenase 1, EC 1.1.1.1 (ADH slow-allele)
**19**	TRINITY_DN41085_c0_g1	50.47	50.02	52.9	257.45	297.21	485.74	-2.76064	2.45E-06	Predicted Protein
**20**	TRINITY_DN3066_c1_g1	13.73	15.11	13.46	55.99	116.42	118.98	-2.74976	6.97E-06	Predicted Protein

**21**	TRINITY_DN2515_c0_g1	42.66	64	22.61	203.34	269.87	276.58	-2.66249	6.45E-07	Probable disease resistance protein At5g04720
**22**	TRINITY_DN1690_c0_g1	82.49	125.29	74.33	334.26	557.98	584.67	-2.43231	2.73E-07	Predicted Protein
**23**	TRINITY_DN1454_c0_g1	36.11	69.11	23.97	190.96	205.3	220.09	-2.42405	3.20E-06	Probable disease resistance protein At4g33300
**24**	TRINITY_DN1454_c0_g1	36.11	69.11	23.97	190.96	205.3	220.09	-2.42405	3.20E-06	Disease resistance protein ADR1 (Activated disease resistance protein 1)
**25**	TRINITY_DN187_c0_g2	34.07	59.55	27.12	157.12	214.46	204.87	-2.36432	8.35E-07	Probable disease resistance protein At4g33300
**26**	TRINITY_DN187_c0_g2	34.07	59.55	27.12	157.12	214.46	204.87	-2.36432	8.35E-07	Probable RNA-binding protein 19 (RNA-binding motif protein 19)
**27**	TRINITY_DN2581_c0_g1	73.03	104.45	74.62	307.26	360.65	549.82	-2.30639	2.39E-06	Ubiquitin-40S ribosomal protein S27a-1 [Cleaved into: Ubiquitin; 40S ribosomal protein S27a-1]
**28**	TRINITY_DN829_c0_g1	36.79	52.72	34.25	136.33	187.57	265.96	-2.27764	4.73E-06	Predicted Protein
**29**	TRINITY_DN871_c0_g1	77.17	92.43	46.57	254.71	317.44	342.34	-2.14567	5.09E-07	Lipase-like PAD4, EC 2.3.1.- (Protein ENHANCED DISEASE SUSCEPTIBILITY 9) (Protein PHYTOALEXIN DEFICIENT 4, AtPAD4)
**30**	TRINITY_DN2157_c0_g1	68.38	76.54	36.2	215.2	233.04	270.04	-2.06397	3.08E-06	Predicted Protein
**31**	TRINITY_DN13745_c0_g1	54.22	67.9	45.99	155.83	243.23	290.83	-2.04992	2.93E-06	Predicted Protein
**32**	TRINITY_DN750_c0_g1	52.81	76.65	41.32	153.78	225.7	259.13	-1.95617	3.05E-06	Probable disease resistance protein At5g04720
**33**	TRINITY_DN3793_c0_g1	366.62	499.73	371.86	1261.79	1399.25	1863.43	-1.92323	3.49E-07	Ubiquitin-40S ribosomal protein S27a-1 [Cleaved into: Ubiquitin; 40S ribosomal protein S27a-1]
**34**	TRINITY_DN3793_c0_g1	366.62	499.73	371.86	1261.79	1399.25	1863.43	-1.92323	3.49E-07	Polyubiquitin [Cleaved into: Ubiquitin]
**35**	TRINITY_DN1147_c0_g1	434.27	562.5	522.82	1377.47	1708.44	2534.14	-1.92099	3.94E-06	Polyubiquitin [Cleaved into: Ubiquitin]
**36**	TRINITY_DN1147_c0_g1	434.27	562.5	522.82	1377.47	1708.44	2534.14	-1.92099	3.94E-06	Ubiquitin-60S ribosomal protein L40 [Cleaved into: Ubiquitin; 60S ribosomal protein L40 (CEP52)]
**37**	TRINITY_DN1147_c0_g1	434.27	562.5	522.82	1377.47	1708.44	2534.14	-1.92099	3.94E-06	Ubiquitin-40S ribosomal protein S27a-1 [Cleaved into: Ubiquitin; 40S ribosomal protein S27a-1]

**38**	TRINITY_DN343_c0_g2	41.37	51.5	29.81	106.48	143.08	171.71	-1.81963	7.75E-06	Disease resistance protein ADR1 (Activated disease resistance protein 1)
**39**	TRINITY_DN3050_c0_g1	94.65	79.26	53.16	190.01	257.33	260.83	-1.67312	6.44E-06	Predicted Protein
**40**	TRINITY_DN5095_c0_g1	139.06	125.09	124.73	305.36	385.7	349.35	-1.44871	6.98E-06	Predicted Protein
